# No association of genetic variants in *TLR4*, *TNF-α*, *IL10*, *IFN-γ*, and *IL37* in cytomegalovirus-positive renal allograft recipients with active CMV infection—Subanalysis of the prospective randomised VIPP study

**DOI:** 10.1371/journal.pone.0246118

**Published:** 2021-04-16

**Authors:** Pascale Mazzola, Elke Schaeffeler, Oliver Witzke, Martin Nitschke, Volker Kliem, Max Zortel, Eva-Maria Wagner, Matthias Schwab, Ingeborg A. Hauser

**Affiliations:** 1 Dr. Margarete Fischer-Bosch-Institute of Clinical Pharmacology (IKP), Stuttgart, Germany; 2 University of Tuebingen, Tuebingen, Germany; 3 Department of Infectious Diseases, West German Centre of Infectious Diseases, Universitätsmedizin Essen, University Duisburg-Essen, Germany; 4 Transplantation Center, Medical Clinic I, University Hospital Schleswig-Holstein, Luebeck, Germany; 5 Department of Internal Medicine and Nephrology, Nephrology Center of Lower Saxony, Klinikum Hann. Muenden, Hann. Muenden, Germany; 6 Roche Pharma AG, Grenzach-Wyhlen, Germany; 7 Departments of Clinical Pharmacology, Pharmacy and Biochemistry, University Tuebingen, Tuebingen, Germany; 8 Department of Nephrology, University Clinic Frankfurt, Goethe University Frankfurt, Frankfurt, Germany; University of St Andrews, UNITED KINGDOM

## Abstract

**Background:**

Cytomegalovirus (CMV) infection is amongst the most important factors complicating solid organ transplantation. In a large prospective randomized clinical trial, valganciclovir prophylaxis reduced the occurrence of CMV infection and disease compared with preemptive therapy in CMV-positive renal allograft recipients (VIPP study; NCT00372229). Here, we present a subanalysis of the VIPP study, investigating single nucleotide polymorphisms (SNPs) in immune-response-related genes and their association with active CMV infection, CMV disease, graft loss or death, rejection, infections, and leukopenia.

**Methods:**

Based on literature research ten SNPs were analyzed for *TLR4*, three for *IFN-γ*, six for *IL10*, nine for *IL37*, and two for *TNF-α*. An asymptotic independence test (Cochran-Armitage trend test) was used to examine associations between SNPs and the occurrence of CMV infection or other negative outcomes. Statistical significance was defined as p<0.05 and Bonferroni correction for multiple testing was performed.

**Results:**

SNPs were analyzed on 116 blood samples. No associations were found between the analyzed SNPs and the occurrence of CMV infection, rejection and leukopenia in all patients. For *IL37* rs2723186, an association with CMV disease (p = 0.0499), for *IL10* rs1800872, with graft loss or death (p = 0.0207) and for *IL10* rs3024496, with infections (p = 0.0258) was observed in all patients, however did not hold true after correction for multiple testing.

**Conclusion:**

The study did not reveal significant associations between the analyzed SNPs and the occurrence of negative outcomes in CMV-positive renal transplant recipients after correction for multiple testing. The results of this association analysis may be of use in guiding future research efforts.

## Introduction

Cytomegalovirus (CMV) infection is the most important serious viral infection that complicates solid organ transplantation [1]. CMV infection can result in CMV disease, which, in severe cases, may lead to hospitalization, morbidity and death [2]. In addition, CMV infection has been associated with secondary effects on the immune system, predisposing patients to opportunistic infections [3, 4], diabetes mellitus, cardiovascular disease and graft loss [5, 6]. Renal transplant recipients without immunity against CMV (R−), who receive an organ from a CMV-positive donor (D+), are at the highest risk of developing CMV disease. Renal transplant recipients with immunity against CMV (R+) are at intermediate risk of active CMV infection [1]. Furthermore, severe organ-invasive CMV disease can also occur in R+ patients and be fatal [7].

Valganciclovir, a valyl ester prodrug of ganciclovir, is currently licensed for the prevention of CMV disease in high-risk (D+/R-) patients receiving a solid organ transplantation [8]. In the largest and longest valganciclovir randomized clinical trial reported to date (VIPP study), prophylaxis was compared with preemptive therapy in CMV R+ renal allograft recipients. The one-year and seven-year results showed that oral valganciclovir prophylaxis significantly reduced CMV infection and disease in intermediate-risk patients compared with preemptive therapy, particularly for CMV-positive donor and recipient (D+/R+) [9, 10]. However, incidences of long-term graft loss and death were similar in the valganciclovir prophylaxis and preemptive treatment groups [10].

Genetic variations in drug targets or in other points of targeted biosynthetic pathways may influence the drug response [11–14]. In renal transplant recipients, polymorphisms in genes whose products are implicated in modulating the human immune response, such as toll-like receptor 4 (TLR4) (Asp299Gly, Tyr399Ile) have been reported to be associated with more frequent serious infectious complications, including severe bacterial infections, CMV disease, and opportunistic infections [15]. Polymorphisms in interleukin (*IL)* 10 can contribute to CMV reactivation and disease after allogeneic stem cell transplantation [16]. Interferon (IFN)-γ plays an important role in the immune response. A statistically significant correlation was found between the *IFN-γ* +874 A>T polymorphism and the risk of CMV infection among 247 Hispanic renal transplant recipients [17]. On the other hand, a protective role against CMV infection has been reported for tumor necrosis factor (*TNF)-α* polymorphisms that are associated with a strong inflammatory response [18]. Anti-inflammatory effects of IL37, a newer member of the IL1 family, were described in a mouse model: Transgenic mice expressing IL37 were protected from colitis [19].

As several studies indicated an association with the occurrence of infection or reported contradictory information about such an association with polymorphisms in *TLR4*, *IFN-γ*, *IL10*, *IL37* or *TNF-α*, we analyzed, as part of the prospective VIPP study mentioned above, single nucleotide polymorphisms (SNPs) in CMV-positive renal allograft recipients.

## Methods

### Design

This project was conducted as a retrospective analysis of the data of the VIPP study, a randomized multicenter trial comparing CMV prophylaxis vs. preemptive therapy with valganciclovir in CMV-positive renal allograft recipients compared in 22 centers in Germany and in 2 centers in Austria. The study included a recruitment period of 29 months, a study phase of 12 months after transplantation, and a follow-up period of 6 years.

(NCT00372229) [9, 10]. Ten SNPs were selected for *TLR4*, three SNPs for *IFN-γ*, six SNPs for *IL10*, nine SNPs for *IL37* and two SNPs for *TNF-α*. Associations between these genetic variants and the occurrence of CMV infection, CMV disease, graft loss or death, rejection, infections, and leukopenia were analyzed.

According to the VIPP study protocol, CMV Disease was defined as either biopsy or clinically proven tissue invasive disease or CMV syndrome with viremia >400 copies/ml with at least one of the following signs: fever of ≥ 38°C, severe malaise (toxicity grading ≥ 3), leucopenia on 2 successive measurements separated by at least 24 hours defined as (1) a white blood cell (WBC) count of <3,500/μL or (2) a WBC decrease of >20% if the WBC count prior to development of viremia is <4,000/μL, atypical lymphocytosis of ≥5%, thrombocytopenia defined as (1) a platelet count of <100,000/μL or (2) a decrease of >20% if the platelet count prior to development of viremia is <115,000/μL, elevation of hepatic transaminases (alanine aminotransferase or aspartate aminotransferase to at least 2x ULN.

### Population

All patients of the VIPP study were eligible for enrollment in this substudy. For inclusion, patients needed to fulfill the following criteria: written informed consent previously obtained for the VIPP study, enrollment in the VIPP main protocol, and written informed consent for the SNP analyses. There were no exclusion criteria. The protocol was approved by the ethics committee of the Hannover Medical School, Nr. 4116M (Hanover, Germany).

### Selection of genetic variants

Various criteria for the selection of SNPs which we considered in the substudy, were used: allele frequencies (>1%) extracted from the Genome Aggregation Database [gnomAD v2.1, http://gnomad.broadinstitute.org/], the location in the coding regions of genes, their clinical relevance [Online Mendelian Inheritance in Man/OMIM, www.omim.org/; ClinVar Short Variants, www.ncbi.nlm.nih.gov/clinvar/; flagged by the SNP database, dbSNP, www.ncbi.nlm.nih.gov/projects/SNP/] as clinically associated), and PubMed records. S1 Table summarizes in detail the selection criteria for the 30 SNPs.

### Blood sampling, DNA extraction, and genotyping

Blood samples could be obtained at any time during the VIPP study, including at scheduled blood sampling timepoints. The samples were frozen and stored at -20°C. The SNP analysis was performed in the Dr. Margarete Fischer-Bosch-Institut für Klinische Pharmakologie (Stuttgart, Germany). Genomic DNA was isolated from whole blood using the QIAamp DNA Blood BioRobot MDx Kit and the QIAamp DSP DNA Blood Mini Kit (Qiagen, Hilden, Germany) according to the manufacturer’s protocol. DNA quality and concentration were assessed using the NanoDrop 1000 Spectrophotometer or the Qubit™ dsDNA BR Assay Kit and a Qubit Fluorometer (Thermo Fisher Scientific, Wilmington, DE, United States). Genetic variants were genotyped by TaqMan technology using predesigned SNP Genotyping assays (Thermo Fisher Scientific) and the Applied Biosystems 7900HT Fast Real-Time PCR System (Thermo Fisher Scientific) according to the manufacturers’ protocols. SNPs were evaluated with Sequence Detection Software version 2.4 (SDS 2.4).

### Statistical analysis

The study was planned for 200 patients. Exploratory analysis was done for all patients and for subgroups of patients receiving valganciclovir prophylaxis or preemptive treatment. Statistical analyses were performed using R version 3.3.2 [20] or SAS 9.4. Tests were two-sided and statistical significance was defined as p<0.05, but results should be interpreted descriptively. Pearson’s Chi-squared test was used to analyze associations between valganciclovir prophylaxis or preemptive treatment and the occurrence of CMV infection, CMV disease, graft loss or death, rejection, infections (viral, bacterial, fungal and other non-CMV infections), and leukopenia. CMV infection was defined as CMV-PCR ≥400 CMV copies/mL from visit 2 (day 7) until follow-up visit 27, excluding unscheduled visits.

Associations between SNPs and the occurrence of CMV infection, CMV disease, graft loss or death, rejection, infections, and leukopenia were examined using an asymptotic independence test (Cochran-Armitage trend test). The trend tests were envisaged for n = 30 genetic variants and 6 endpoints in the overall cohort and two subgroups (prophylaxis and preemptive treatment). For two variants (IFN-γ rs2069723 and IL10 rs3024489), no nucleotide changes were observed in our cohort. Only two events occurred for CMV disease in the prophylaxis group. The effective number of tests was therefore determined as n = 476 (= 28*17), and the corresponding Bonferroni corrected significance level consequently as α* = 0.05/476 ≈ 0.00011. Odds-Ratios (OR) and 95% confidence intervals (95% CI) were calculated using a logistic regression model as an association between genotypes and endpoints. Patients with missing data were excluded. Lower OR correspond to lower odds of seeing an event in the largest genotype group.

Haplotype analyses were performed with R-library haplo.stats [21]. Haplotypes were estimated separately for each gene, using all studied variants. Associations between haplotypes and the occurrence of CMV infection, CMV disease, graft loss or death, rejection, infections, and leukopenia were investigated using logistic regression and an additive genetic model. The minimal haplotype frequency for a haplotype to be included as a separate term in the regression model was set to 5%. Results with unusually large coefficients from the logistic regression fit (occurring for dependent variables with less than 15 events or haplotype frequencies close to 5% in treatment subgroup analyses) were considered as unreliable and therefore disregarded.

## Results

### Patients

**Fig 1:**
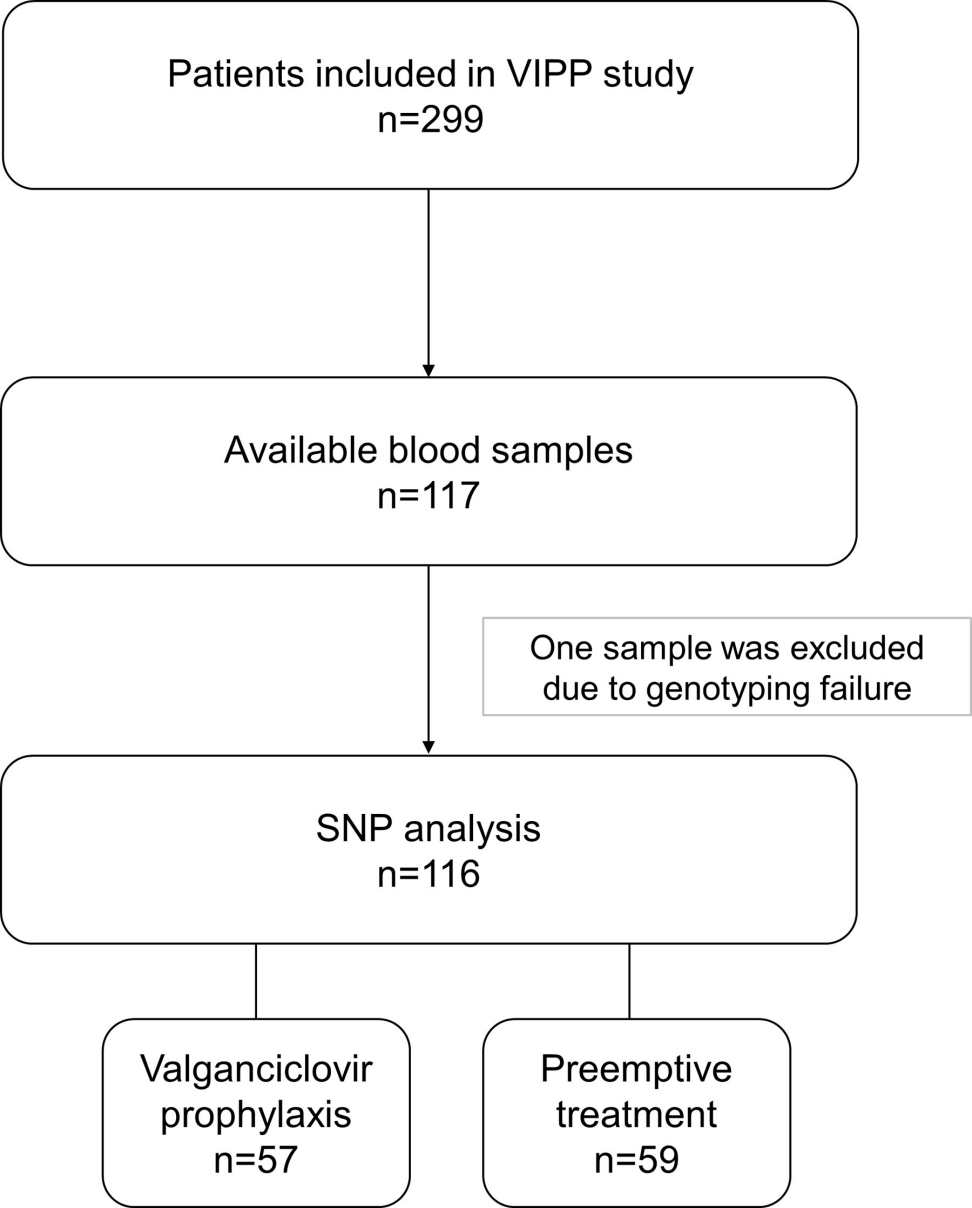
Disposition of patients and samples analyzed. SNP, single nucleotide polymorphism; VIPP, ValgancIclovir Prophylaxis versus Preemptive therapy in cytomegalovirus-positive renal allograft recipients.

Blood samples were collected between March 2009 and January 2014 within the VIPP study. In total, 117 of 299 patients provided blood samples for the analysis. The SNP analysis could be performed on 116 of these samples (**Fig 1**).

The patient characteristics are presented in **Table 1**. The samples were equally distributed between the prophylaxis and the preemptive treatment group (57 *vs*. 59 patients), with generally balanced demographic and clinical baseline characteristics. The mean age for all patients was 51.0 years, and more total male than female patients were included in the SNP analysis.

**Table 1:** Demographic and clinical characteristics at baseline.

Characteristic	All (n = 116)	Prophylaxis (n = 57)	Preemptive (n = 59)
Recipient age (years)	51.0 ± 12.4	49.5 ± 13.2	52.5 ± 11.5
Donor age (years)^a^	52.7 ± 14.7	52.3 ± 15.6	53.1 ± 13.9
Living donor, n (%)	12 (10.3)	8 (14.0)	4 (6.8)
Male sex, n (%)	76 (65.5)	41 (71.9)	35 (59.3)
Caucasian, n (%)	110 (94.8)	53 (93.0)	57 (96.6)
Body weight (kg)^b^	75.7 ± 14.6	77.0 ± 16.0	74.4 ± 13.2
Sensitization of recipients against donor antigens^c^ (%)	1.0 ± 8.6	0.1 ± 0.6	1.9 ± 12.0
Hypertension^d^, n (%)	87 (75.0)	46 (80.7)	41 (69.5)
Diabetes mellitus^d^, n (%)	13 (11.2)	7 (12.3)	6 (10.2)
Stratification for ATG/ALG, n (%)	3 (2.6)	2 (3.5)	1 (1.7)
Cold ischemia time (hours)	12.2 ± 6.8	11.9 ± 7.3	12.5 ± 6.3
HLA-A + B + DR mismatch	2.6 ± 1.5	2.6 ± 1.5	2.6 ± 1.6
Previous transplants, n (%)			
0	97 (83.6)	49 (86.0)	48 (81.4)
1	19 (16.4)	8 (14.0)	11 (18.6)
Most frequent underlying kidney diseases^e^, n (%)			
Glomerulonephritis	24 (20.7)	7 (12.3)	17 (28.8)
Adult polycystic kidney disease	14 (12.1)	9 (15.8)	5 (8.5)
IgA nephropathy	19 (16.4)	9 (15.8)	10 (16.9)
Diabetic glomerulosclerosis/nephropathy	6 (5.2)	3 (5.3)	3 (5.1)
Others	33 (28.4)	16 (28.1)	17 (28.8)
CMV status donor/recipient, n (%)			
D+/R+	65 (56.0)	37 (64.9)	28 (47.5)
D-/R+	51 (44.0)	20 (35.1)	31 (52.5)

Values are means ± standard deviation unless otherwise indicated.

^a^All: n = 115, prophylaxis: n = 56, preemptive: n = 59, due to missing value

^b^All: n = 115, prophylaxis: n = 57, preemptive: n = 58, due to missing value.

^c^Estimated by the actual panel-reactive antibody test. All: n = 83, prophylaxis: n = 41, preemptive: n = 42.

^d^Previous or concomitant disease.

^e^Occurrence in ≥3% of all patients.

ALG, antilymphocyte globulin; ATG, antithymocyte globulin; CMV, cytomegalovirus; D/R, donor/recipient; HLA, human leukocyte antigen; IgA, immunoglobulin A.

### Occurrence of CMV infection, CMV disease, graft loss or death, rejection, infections, and leukopenia in the prophylaxis versus preemptive treatment group

The proportion of patients with CMV infection and CMV disease was higher in the preemptive treatment group than in the valganciclovir prophylaxis group (CMV infection: 35.6% vs. 14.0%, p = 0.014, 29 events in total; CMV disease: 7.8% vs. 1.7%, p = 0.031, 11 events in total). For occurrence of graft loss or death (15 events), rejection (93 events), infections (85 events), and leukopenia (32 events), no remarkable differences were observed between both groups.

### Frequencies of genetic variants in all patients

Ten SNPs were analyzed for *TLR4*, three SNPs for *IFN-γ*, six SNPs for *IL10*, nine SNPs for *IL37* and two SNPs for *TNF-α*. Selected SNPs, genotype and allele frequencies for all patients are provided in the **S2 Table**. Genotype frequencies of all SNPs are in Hardy-Weinberg equilibrium.

### Association of genetic variants with CMV infection, CMV disease, graft loss or death, rejection, infections, and leukopenia in all patients

P-values were >0.05 for all analyzed SNPs and the occurrence of CMV infection, rejection and leukopenia (**Tables 2 and 3**). For *IL37* 2723186, an association with CMV disease was observed (p = 0.0499, OR 3.59, 95% CI 0.36–35.82). *IL10* rs1800872 was associated with graft loss or death (p = 0.0207, OR 3.04, 95% CI 1.08–8.61). For *IL10* rs3024496, an association with infections was observed (p = 0.0258, OR 1.63, 95% CI 0.725–3.64). All significant results did not hold true after Bonferroni correction.

**Table 2:** Statistical analysis of the association between genetic variants and the clinical events CMV infection, CMV disease and infections in all patients, and in the prophylaxis and preemptive treatment group.

		CMV infection			CMV disease			Infections		
SNP	Nucleotide change*	All p-value	Proph. p-value	Preem. p-value	All p-value	Proph. p-value^‡^	Preem. p-value	All p-value	Proph. p-value	Preem. p-value
		n/n^†^	n/n^†^	n/n^†^	n/n^†^	n/n^†^	n/n^†^	n/n^†^	n/n^†^	n/n^†^
*TLR4*_rs4986790	g.13843A>G	0.5118	0.3325	0.9157	0.2348	0.6330	0.2556	0.2838	0.8604	0.1022
		29/114	8/55	21/59	11/114	2/55	9/59	84/114	41/55	43/59
*TLR4*_rs4986791	g.14143C>T	0.2925	0.3325	0.4407	0.2479	0.6330	0.2729	0.3801	0.8604	0.1149
		29/114	8/55	21/59	11/114	2/55	9/59	84/114	41/55	43/59
*TLR4*_rs5030710	g.13262T>C	0.3102	NA^§^	0.1901	0.5681	NA^§^	0.4507	0.7721	NA^§^	0.8037
		29/115	8/56	21/59	11/115	2/56	9/59	85/115	42/56	43/59
*TLR4*_rs7869402	g.16573C>T	0.2587	0.6799	0.2638	0.5037	0.8446	0.4459	0.2795	0.5554	0.1199
		29/113	8/55	21/58	11/113	2/55	9/58	83/113	41/55	42/58
*TLR4*_rs7873784	g.17477G>C	0.8143	0.5697	0.8880	0.7593	0.2799	0.7945	0.7328	0.7510	0.4875
		28/112	8/55	20/57	11/112	2/55	9/57	83/112	42/55	41/57
*TLR4*_rs11536871	g.9039A>C	0.1123	0.3958	0.1901	0.3721	0.6866	0.4507	0.0559	0.2419	0.1138
		29/114	8/55	21/59	11/114	2/55	9/59	84/114	41/55	43/59
*TLR4*_rs11536887	g.16215A>G	0.5545	0.6767	NA^§^	0.7403	0.8431	NA^§^	0.5527	0.5698	NA^§^
		29/112	8/54	21/58	11/112	2/54	9/58	83/112	41/54	42/58
*TLR4*_rs11536889	g.16672G>C	0.3219	0.4372	0.8338	0.3320	0.3095	0.8106	0.0664	0.7817	**0.0082**^¶^
		29/115	8/54	21/59	11/115	2/56	9/59	85/115	42/56	43/59
*TLR4*_rs11536891	g.17878T>C	0.7480	0.4831	0.9590	0.8002	0.4043	0.8706	0.7038	0.2430	0.5593
		29/113	8/56	21/57	11/113	2/56	9/57	85/113	42/56	43/57
*TLR4*_rs11536892	g.18023G>A	0.5614	NA^§^	0.4572	0.7439	NA^§^	0.6687	0.0909	NA^§^	0.0982
		29/115	8/56	21/59	11/115	2/56	9/59	85/115	42/56	43/59
*IFN-γ*_rs2069707	g.4234C>G	0.8586	0.4224	0.6716	0.1676	1.0000	0.2966	0.8769	1.0000	0.9266
		29/114	8/55	21/59	11/114	2/55	9/59	85/114	42/55	43/59
*IFN-γ*_rs2069723	g.9928A>G	NA^§^	NA^§^	NA^§^	NA^§^	NA^§^	NA^§^	NA^§^	NA^§^	NA^§^
		29/114	8/55	21/59	11/114	2/55	9/59	84/114	41/55	43/59
*IFN-γ*_rs2430561	g.6000A>T	0.2821	0.1729	0.4540	0.2060	0.1610	0.3220	0.9635	0.2212	0.2839
		29/114	8/57	21/57	11/114	2/57	9/57	83/114	42/57	41/57
*IL10*_rs1800871	g.4206T>C	0.2093	0.5349	0.1975	0.1460	0.2333	0.3130	0.1584	0.0771	0.8477
		28/114	7/55	21/59	11/114	2/55	9/59	84/114	41/55	43/59
*IL10*_rs1800872	g.4433A>C	0.3458	0.6463	0.3710	0.2412	0.8784	0.2821	0.9756	0.9269	0.9377
		29/115	8/56	21/59	11/115	2/56	9/59	85/115	42/**56**	43/59
*IL10*_rs1800894	g.4174G>A	0.8808	0.5288	0.7218	0.9591	0.6896	0.9474	0.1604	1.0000	0.0981
		29/115	8/56	21/59	11/115	2/56	9/59	85/115	42/56	43/59
*IL10*_rs3024489	g.4596G>T	NA^§^	NA^§^	NA^§^	NA^§^	NA^§^	NA^§^	NA^§^	NA^§^	NA^§^
		29/113	8/54	21/59	11/113	2/54	9/59	84/113	41/54	43/59
*IL10*_rs3024496	g.8976T>C	0.2235	0.5353	0.3041	0.3293	0.7507	0.1778	**0.0258**^ǁ^	0.0111**	0.4730
		28/112	7/53	21/59	11/112	2/53	9/59	84/112	53	43/59
*IL10*_rs3024498	g.9311A>G	0.7548	0.5481	0.8859	0.1761	0.3247	0.2349	0.8437	0.2454	0.1842
		28/112	8/54	20/58	11/112	2/54	9/58	82/112	40/54	42/58
*IL37*_rs2708943	g.9162C>G	0.9678	0.5417	0.4388	0.6971	**0.0178**^††^	0.4969	0.4886	0.2249	0.8664
		29/114	8/55	21/59	11/114	2/55	9/59	84/114	41/55	43/59
*IL37*_rs2708947	g.10672T>C	0.8902	0.7045	0.6878	0.5946	**0.0381**^‡‡^	0.6121	0.9563	0.7867	0.8221
		29/115	8/56	21/59	11/115	2/56	9/59	85/115	42/56	43/59
*IL37*_rs2723171	g.3256G>C	0.9829	0.7045	0.5264	0.6671	**0.0381**^§§^	0.5285	0.9150	0.7867	0.9898
		29/115	8/56	21/59	11/115	2/56	9/59	85/115	42/56	43/59
*IL37*_rs2723183	g.9174A>G	0.9243	0.7192	0.6563	0.6136	**0.0404**^¶¶^	0.5963	0.9911	0.7690	0.8511
		29/113	8/55	21/58	11/113	2/55	9/58	83/113	41/55	42/58
*IL37*_rs2723186	g.9533A>G	0.5303	0.1003	0.4572	**0.0499**^ǁǁ^	0.00004***	0.6687	0.5285	0.9711	0.0982
		29/113	8/54	21/59	11/113	2/54	9/59	84/113	41/54	43/59
*IL37*_rs2723187	g.9722C>T	0.8549	0.7192	0.4388	0.7640	**0.0404**^†††^	0.4969	0.7909	0.7690	0.8664
		29/114	8/55	21/49	11/114	2/55	9/59	84/114	41/55	43/59
*IL37*_rs2723192	g.10834G>A	0.9071	0.7192	0.6878	0.6041	**0.0404**^‡‡‡^	0.6121	0.9736	0.7690	0.8221
		29/114	8/55	21/59	11/114	2/55	9/59	84/114	41/55	43/59
*IL37*_rs3811046	g.5831G>T	0.5652	0.0651	0.4636	0.7663	0.0573	0.4726	0.9074	1.0000	0.8627
		29/114	8/56	21/58	11/114	2/56	9/58	84/114	42/56	42/58
*IL37*_rs3811047	g.5863A>G	0.5859	0.1348	0.5817	0.6982	**0.0431**^§§§^	0.4726	0.9680	0.8275	0.8627
		29/112	8/54	21/58	11/112	2/54	9/58	82/112	40/54	42/58
*TNF-α*_rs1800629	g.4682G>A	0.3230	0.8424	0.2298	0.4078	0.6703	0.4270	0.4372	0.1185	0.6212
		29/115	8/56	21/59	11/115	2/56	9/59	85/115	42/56	43/59
*TNF-α*_rs3093665	g.7042A>C	0.4821	0.3910	0.8793	0.8220	0.7183	0.7869	0.3160	0.2721	0.6741
		29/108	8/51	21/57	11/108	2/51	9/57	78/108	37/51	41/57

NA, not applicable; preem., preemptive; proph., prophylaxis; SNP, single nucleotide polymorphism, Odds ratio (OR), 95% Confidence interval (CI). Asymptotic Cochran-Armitage trend test was used.

P-values <0.05 in bold. All p-values were >0.00011 (Bonferroni correction for multiple testing).

*Nucleotide change refers to the coding reference sequence.

^†^Number of patients with an event and total number of patients included for the statistical analysis.

^‡^Due to the low numbers of CMV disease events in the prophylaxis group (only two events) Bonferroni correction was not performed.

^§^NA since all patients of this study-group carrying the same genotype.

^¶^OR 0.194, 95% CI 0.055–0.69.

^ǁ^OR 1.625, 95% CI 0.725–3.64.

**OR 6.73, 95% CI 1.61–28.20.

^††^OR 16.67, 95% CI 0.824–337.01.

^‡‡^OR 12.50, 95% CI 0.652–239.54.

^§§^OR 12.50, 95% CI 0.652–239.54.

^¶¶^OR 12.25, 95% CI 0.639–234.82.

^ǁǁ^OR 3.591, 95% CI 0.36–35.823.

***OR 50.99, 95% CI 2.40-Inf.

^†††^OR 12.25, 95% CI 0.639–234.82.

^‡‡‡^OR 12.25, 95% CI 0.639–234.82.

^§§§^OR 12.23, 95% CI 0.728–205.42.

^¶¶¶^OR 12.25, 95% CI 0.639–234.82.

**Table 3:** Statistical analysis of the association between genetic variants and the clinical events graft loss or death, rejection and leukopenia in all patients, and in the prophylaxis and preemptive treatment group.

		Graft loss or death			Rejection			Leukopenia		
SNP	Nucleotide change*	All p-value	Proph. p-value	Preem. p-value	All p-value	Proph. p-value	Preem. p-value	All p-value	Proph. p-value	Preem. p-value
		n/n^†^	n/n^†^	n/n^†^	n/n^†^	n/n^†^	n/n^†^	n/n^†^	n/n^†^	n/n^†^
*TLR4*_rs4986790	g.13843A>G	0.1571	0.2769	0.3673	0.5827	0.3479	0.9792	0.5624	0.2009	0.6018
		15/114	9/55	6/59	92/114	48/55	44/59	30/114	18/55	12/59
*TLR4*_rs4986791	g.14143C>T	0.1858	0.3105	0.3845	0.7259	0.3479	0.6387	0.3705	0.2427	0.8136
		14/114	8/55	6/59	92/114	48/55	44/59	31/114	19/55	12/59
*TLR4*_rs5030710	g.13262T>C	0.4967	NA^§^	0.5497	0.3933	NA^§^	0.2993	0.2863	NA^§^	0.3690
		15/115	9/56	6/59	93/115	49/56	44/59	31/115	19/56	12/59
*TLR4*_rs7869402	g.16573C>T	0.4816	**0.0225**^¶^	0.5457	0.3167	0.6999	0.2935	0.2315	0.4815	0.3895
		15/113	9/55	6/58	91/113	48/55	43/58	29/113	18/55	11/58
*TLR4*_rs7873784	g.17477G>C	0.8773	0.7021	0.6268	0.6470	0.8288	0.5792	0.1445	0.2019	0.4641
		14/112	8/55	6/57	91/112	49/55	42/57	31/112	19/55	12/57
*TLR4*_rs11536871	g.9039A>C	0.2130	0.6278	0.1730	0.5211	0.4444	0.7467	0.4556	0.7323	0.3690
		15/114	9/55	6/59	92/114	48/55	44/59	30/114	18/55	12/59
*TLR4*_rs11536887	g.16215A>G	0.6928	0.6517	NA^§^	0.6194	0.6969	NA	0.5435	0.4754	NA^§^
		15/112	9/54	6/58	90/112	47/54	43/58	30/112	18/54	12/58
*TLR4*_rs11536889	g.16672G>C	0.5181	0.7263	0.1447	0.6640	0.8560	0.4153	0.6681	0.4579	0.1477
		15/115	9/56	6/59	93/115	49/56	44/59	31/115	19/56	12/59
*TLR4*_rs11536891	g.17878T>C	0.3996	0.1329	0.6632	0.7048	0.2297	0.6611	0.1233	0.1413	0.5120
		15/113	9/56	6/57	91/113	49/56	42/57	31/113	19/56	12/57
*TLR4*_rs11536892	g.18023G>A	0.6973	NA^§^	0.7343	0.6252	NA^§^	0.5559	0.5418	NA^§^	0.6103
		15/115	9/56	6/59	93/115	49/56	44/59	31/115	19/56	12/59
*IFN-γ*_rs2069707	g.4234C>G	0.9427	0.1765	0.0862	0.1987	0.1336	**0.0401**^ǁ^	0.4485	1.0000	0.1149
		15/114	9/55	6/59	92/114	48/55	44/59	31/114	19/55	12/59
*IFN-γ*_rs2069723	g.9928A>G	NA^§^	NA^§^	NA^§^	NA^§^	NA^§^	NA^§^	NA^§^	NA^§^	NA^§^
		14/114	8/55	6/59	92/114	48/55	44/59	31/114	19/55	12/59
*IFN-γ*_rs2430561	g.6000A>T	0.6879	0.8932	0.5563	0.4006	0.1729	0.8859	0.3261	0.6566	0.0764
		15/114	9/57	6/57	93/114	49/57	44/57	32/114	20/57	12/57
*IL10*_rs1800871	g.4206T>C	0.0767	0.1002	0.4236	0.3383	0.3043	0.5898	0.3098	0.5911	0.3769
		15/114	9/55	6/59	93/114	49/55	44/59	31/114	19/55	12/59
*IL10*_rs1800872	g.4433A>C	0.0207**	**0.0037**^††^	0.5812	0.5464	0.9521	0.3547	0.1966	0.2831	0.5966
		15/115	9/56	6/59	93/115	49/56	44/59	31/115	19/56	12/59
*IL10*_rs1800894	g.4174G>A	0.6273	0.3638	0.1359	0.5100	0.4328	0.8595	0.4999	0.6955	0.1707
		15/115	9/56	6/59	93/115	49/56	44/59	31/115	19/56	12/59
*IL10*_rs3024489	g.4596G>T	NA^§^	NA^§^	NA^§^	NA^§^	NA^§^	NA^§^	NA^§^	NA^§^	NA^§^
		15/113	9/54	6/59	91/113	47/54	44/59	30/113	18/54	12/59
*IL10*_rs3024496	g.8976T>C	0.3191	0.2406	0.9144	0.9153	0.3403	0.4156	0.1309	0.1489	0.4811
		15/112	9/53	6/59	90/112	46/53	44/59	28/112	16/53	12/59
*IL10*_rs3024498	g.9311A>G	0.1424	0.1244	0.6925	0.6220	0.3501	0.9062	0.9558	0.5622	0.5521
		15/112	9/54	6/58	91/112	48/54	43/58	29/112	17/54	12/58
*IL37*_rs2708943	g.9162C>G	0.4101	0.6278	0.2350	0.5603	0.4277	0.5214	0.6515	0.0613	0.7474
		15/114	9/55	6/59	92/114	48/55	44/59	30/114	18/55	12/59
*IL37*_rs2708947	g.10672T>C	0.4316	0.8021	0.2430	0.4262	0.3758	0.2454	0.5506	0.1970	0.9767
		15/115	9/56	6/59	93/115	49/56	44/59	31/115	19/56	12/59
*IL37*_rs2723171	g.3256G>C	0.3845	0.8021	0.2160	0.5200	0.3758	0.3556	0.6766	0.1970	0.8438
		15/115	9/56	6/59	93/115	49/56	44/59	31/115	19/56	12/59
*IL37*_rs2723183	g.9174A>G	0.4180	0.8177	0.2377	0.3875	0.3705	0.1976	0.5228	0.1728	0.9528
		15/113	9/55	6/58	93/113	49/55	44/58	30/113	18/55	12/58
*IL37*_rs2723186	g.9533A>G	0.4525	0.4538	0.7343	0.3450	0.5180	0.5559	0.5586	0.5537	0.6103
		15/113	9/54	6/59	91/113	47/54	44/59	30/113	18/54	12/59
*IL37*_rs2723187	g.9722C>T	0.3672	0.8177	0.2350	0.2830	0.3705	0.1880	0.8345	0.2093	0.7474
		15/114	9/55	6/59	93/114	49/55	44/59	31/114	19/55	12/59
*IL37*_rs2723192	g.10834G>A	0.4248	0.8177	0.2430	0.4378	0.3705	0.2454	0.5078	0.1728	0.9767
		15/114	9/55	6/59	93/114	49/55	44/59	30/114	18/55	12/59
*IL37*_rs3811046	g.5831G>T	0.2698	0.9411	0.0920	0.6565	0.5657	0.9233	0.3573	0.7309	0.3060
		15/114	9/56	6/58	92/114	49/56	43/58	31/114	19/56	12/58
*IL37*_rs3811047	g.5863A>G	0.1517	0.6574	0.0920	0.9456	0.8885	0.9233	0.4533	0.9559	0.3060
		15/112	9/54	6/58	91/112	48/54	43/58	29/112	17/54	12/58
*TNF-α*_rs1800629	g.4682G>A	0.2792	0.1927	0.9536	0.5649	0.3068	0.9196	0.3672	0.8167	0.2299
		15/115	9/56	6/59	93/115	49/56	44/59	31/115	19/56	12/59
*TNF-α*_rs3093665	g.7042A>C	0.2378	0.4085	0.4220	0.6799	0.5145	0.4669	0.9505	0.2274	0.2766
		15/108	9/51	6/57	87/108	45/51	42/57	28/108	16/51	12/57

*Nucleotide change refers to the coding reference sequence.

^†^Number of patients with an event and total number of patients included for the statistical analysis.

^‡^Due to the low numbers of CMV disease events in the prophylaxis group (only two events) Bonferroni correction was not performed.

^§^NA since all patients of this study-group carrying the same genotype.

^¶^OR Inf, 95% CI 0-Inf.

^ǁ^OR 0.201, 95% CI 0.039–1.035.

**OR 3.043, 95% CI 1.076–8.61.

^††^OR 5.342, 95% CI 1.331–21.45.

### Association of genetic variants with the occurrence of events in the prophylaxis versus preemptive treatment group

Associations of SNPs with the occurrence of CMV infection, CMV disease, graft loss or death, rejection, infections, and leukopenia were also investigated in the prophylaxis and preemptive treatment group. In the prophylaxis group, *TLR4* rs7869402 and *IL10* rs1800872 were associated with the occurrence of graft loss or death (p = 0.0255, OR Inf., 95% CI 0-Inf. and p = 0.0037, OR 5.34, 95% CI 1.33–21.45), *and IL10_rs30*24496 was associated with infections (p = 0.0111, OR 6.73, 95% CI 1.61–28.2) (**Tables 2 and 3**). Additionally, in the prophylaxis group, associations with CMV disease were observed for nearly all analyzed *IL37* SNPs. However, only two CMV disease events were observed in this subgroup. In the preemptive group, we found associations with infections for *TLR4* rs11536889 (p = 0.0082, OR 0.194, 95% CI 0.055–0.693) and with rejection for *IFN-γ* rs2069707 (p = 0.201, 95% OR 0.039–1.035). Again, all significant results did not hold true after Bonferroni correction. For all other investigated SNPs and events, p-values were >0.05.

### Association of haplotypes with the occurrence of events in all patients and the prophylaxis versus preemptive treatment group

In all patients, nominal significant differences between the most frequent haplotype and any of the other non-rare haplotypes (haplotype frequency ≥5%) identified were revealed for endpoint rejection and *IFN-γ* (p = 0.023) as well as infections and *IL10* (p = 0.017). Moreover, the association between infections and *TLR4* showed a borderline effect in all patients (p = 0.051) and was nominally significant in the preemptive treatment group (p = 0.038). However, in all three cases, the differing haplotype was determined by just one variant, which we already identified in our previous per-variant analysis (Tables 2 and 3, *IFN-γ* rs2069707, *IL10* rs3024496, and *TLR4* rs11536889, respectively). In addition, in the preemptive treatment group, a nominally significant association was identified between leukopenia and *IFN-γ* (p = 0.049). Here, the differing haplotype was solely determined by *IFN-γ* rs2430561. In the corresponding per-variant analysis, the association between *IFN-γ* rs2430561 and leukopenia showed a borderline effect (p = 0.0764, Table 3). Our haplotype analyses did not reveal any nominal significant associations for CMV infection, CMV disease as well as graft loss or death. All haplotype analyses results are not statistically significant after adjustment for multiple testing.

## Discussion

This is the first association study of common genetic variants in patients after renal transplantation in a prospective randomized clinical trial.

The characteristics of the 116 patients, from whom blood samples were analyzed, were comparable to the overall population of the VIPP study, which comprised 299 patients [10]. The proportion of patients with CMV infection and CMV disease was higher in the preemptive treatment group than in the valganciclovir prophylaxis group (35.6% vs. 14.0% and 7.8% vs 1.7%). The percentages of CMV infection events in this analysis were comparable to the results seen in the overall population of the VIPP study after 84 months (incidence of CMV infection: 39.7% in the preemptive group vs. 11.5% in the prophylaxis group; p<0.0001) [10]. The percentages of CMV disease events were lower in this substudy than in the overall population (incidence of CMV disease in VIPP study after 84 months: 15.9% in the preemptive group vs. 4.7% in the prophylaxis group; p<0.01). Due to the low numbers of CMV disease events, analyses of CMV disease in the prophylaxis group (two events) were excluded from the Bonferroni correction.

The selection of the SNPs which were tested in this substudy was based on various criteria. One criterium considered the allele frequencies of variants >1%. Population frequency data were obtained from the Genome Aggregation Database (gnomAD v2.1) which comprises data from more than 140,000 individuals including their ethnic background. Of note total allele frequency data of the selected variants corresponds very well with allele frequency data of European (non-Finnish) individuals (> 60,000).

For CMV infection, no association with the investigated SNPs in the genes *TLR4*, *IL10*, *IFN-γ*, *IL37* and *TNF-α* was observed in this largely Caucasian population. Regarding *IFN-γ*, our results are in line with data by Aguado et al. [22], demonstrating no association between the +874 T/A (rs2430561) variant and CMV replication in R+ transplant patients. However, other studies demonstrated an association between SNPs in these genes and infection or CMV disease [15–18]. This can be partially explained by differences in the study populations, conditions and/or specific locations of investigated SNPs. Vu et al. observed an association of the *IFN-γ* polymorphism +874 A/T (rs2430561) with an increased risk of CMV infection in transplant recipients in a Hispanic population [18]. Mitsani et al. [23] also found a correlation of the *IFN-γ* +874 T/T genotype and CMV disease after lung transplantation, which was most striking among R+ patients. In another study, associations were found between other *IL10* SNPs (rs1800896, rs3024492 and rs1878672) and the development of CMV disease [16].

For the further endpoints of interest, associations were observed in all patients for *IL37* rs2723186 with CMV disease, for *IL10* rs1800872 with the occurrence of graft loss or death, and for *IL10* rs3024496 with the occurrence of infections. These results were confirmed in the prophylaxis group, however did not hold true after Bonferroni correction.

IL-37 is capable of reducing the activity of both innate and specific immune responses [24, 25]. *IL37* rs2723186 is located in the intronic region of the gene. Previously, it was shown that the rs2723186 allele was significantly associated with a decreased risk of Graves’disease (an autoimmune thyroid disease) in female patients [26]. In addition, the AG genotype of rs2723186, compared to the GG genotype exhibited a decreased risk of hepatitis B virus infection [27]. In our analysis, ten patients with the GG genotype and one patient with the AA genotype developed CMV disease.

*IL10* rs1800872 (-592C>A) is located in the promotor region of the gene and part of the A-T-A (-1082/-819/-592) haplotype, whose impact on the production of IL10 is discussed controversially [28–30]. In our analysis, patients with the GG genotype experienced fewer events of graft loss or death. *IL10* rs3024496 is located in the 3’ untranslated region of the gene and it was previously shown to influence CD4^+^ T cell responses to antiretroviral therapy in patients with human immunodeficiency virus 1 infection [31].

The herein described study has the following limitations: The number of analyzed blood samples was smaller than the planned number of 200 patients, and no sample size calculation was performed in advance. The study population cannot be extended by additional patient samples since the VIPP study was a prospective randomised clinical trial. Furthermore, the study was not designed for the large number of statistical tests. Additionally, the number of events was low, particularly for CMV disease, and graft loss or death. Conversely, intensive immunosuppression may have hidden possible associations.

The results of this association analysis may guide future research efforts. Based on the complexity of the immune response, research on the combined use of biomarkers and gene profiles represents a better approach than the focus on single markers. Immunity against CMV is usually defined by serology. However, a cellular immune response with potent CMV-specific T cells seems to help prevent CMV disease in CMV R+ recipients. CMV-specific T-cell responses are not measured routinely in clinics, but their potential diagnostic value has been demonstrated [32–34]. T-cell response monitoring may be useful to guide prophylaxis and preemptive treatment. Thus, additional data regarding the use of biomarkers for risk stratification of renal transplant recipients should be explored [1].

## Supporting information

S1 Checklist VIPP CONSORT 2010 checklist. (DOC)

S1 Table Overview about total allele frequencies for the selected variants. (DOCX)

S2 Table Genetic variants and allele and genotype frequencies of all patients and separated study groups. (DOCX)

S1 File Protocol amended VAC-CTA-ML19313-amd11-20100924. (PDF)

## Abbreviations

D/R: donor/recipient
CMV: cytomegalovirus
IFN: interferon
IL: interleukin
SNP: single nucleotide polymorphism
TLR: toll like receptor
TNF: tumor necrosis factor
