# Supplementary material for: No association of genetic variants in TLR4, TNF-α, IL10, IFN-γ, and IL37 in cytomegalovirus-positive renal allograft recipients with active CMV infection—Subanalysis of the prospective randomised VIPP study
Source: PLoS One. 2021 Apr 16;16(4):e0246118. doi: 10.1371/journal.pone.0246118 (PMC8051780; doi:10.1371/journal.pone.0246118)
Supplement: S1 Table — (DOCX) [file pone.0246118.s002.docx]

| **Genetic variant** | **Function/location** | **Population allele frequencies** | | **PubMed records** | **Clinically relevant** |
| --- | --- | --- | --- | --- | --- |
|  |  | **European (non-Finnish)** | **Total** |  |  |
| *TLR4*_rs4986790 | missense | 0.05575 | 0.06121 | + |  |
| *TLR4*_rs4986791 | missense | 0.05863 | 0.05603 | + |  |
| *TLR4*_rs5030710 | synonymous | 0.0007151 | 0.01509 | + |  |
| *TLR4*_rs7869402 | 3’UTR | 0.02522 | 0.09311 | + |  |
| *TLR4*_rs7873784 | 3’UTR | 0.1502 | 0.1579 | + |  |
| *TLR4*_rs11536871 | intronic | 0.03242 | 0.02214 | + |  |
| *TLR4*_rs11536887 | 3’UTR | 0.0001944 | 0.02656 |  |  |
| *TLR4*_rs11536889 | 3’UTR | 0.1399 | 0.1082 | + |  |
| *TLR4*_rs11536891 | 3’UTR | 0.1501 | 0.1587 | + |  |
| *TLR4*_rs11536892 | 3’UTR | 0.0005189 | 0.009528 |  |  |
| *IFN-γ*_rs2069707 | 5’near gene | 0.05916 | 0.03889 | + | + |
| *IFN-γ*_rs2069723 | 3’UTR | 0 | 0.01048 |  |  |
| *IFN-γ*_rs2430561 | intronic | 0.4511 | 0.3578 | + |  |
| *IL10*_rs1800871 | 5’near gene | 0.764 | 0.6872 | + |  |
| *IL10*_rs1800872 | 5’near gene | 0.7646 | 0.6869 | + | + |
| *IL10*_rs1800894 | 5’near gene | 0.03138 | 0.0214 | + |  |
| *IL10*_rs3024489 | 5’near gene | 0.00006485 | 0.01377 | + |  |
| *IL10*_rs3024496 | 3’UTR | 0.4733 | 0.4211 | + |  |
| *IL10*_rs3024498 | 3’UTR | 0.264 | 0.2013 | + |  |
| *IL37*_rs2708943 | missense | 0.08177 | 0.07179 |  |  |
| *IL37*_rs2708947 | missense | 0.08184 | 0.07181 |  |  |
| *IL37*_rs2723171 | 5’near gene | 0.08034 | 0.09916 |  |  |
| *IL37*_rs2723183 | missense | 0.0818 | 0.07179 |  |  |
| *IL37*_rs2723186 | intronic | 0.998 | 0.9102 | + |  |
| *IL37*_rs2723187 | missense | 0.08167 | 0.0718 |  |  |
| *IL37*_rs2723192 | missense | 0.08177 | 0.07167 |  |  |
| *IL37*_rs3811046 | missense | 0.6999 | 0.6941 | + |  |
| *IL37*_rs3811047 | missense | 0.6999 | 0.6993 | + |  |
| *TNF-α*_rs1800629 | 5’near gene | 0.1649 | 0.1216 | + | + |
| *TNF-α*_rs3093665 | 3’UTR | 0.02043 | 0.01941 | + |  |

# S1 Table.

# Overview about total allele frequencies for the selected variants based on the [Genome Aggregation Database](https://gnomad.broadinstitute.org/about) (gnomAD v2.1, <https://gnomad.broadinstitute.org/about>) comprising more than 140,000 individuals. Total allele frequencies were compared to allele frequencies in a European (non-Finnish) population (>60,000 individuals).
