# Supplementary material for: No association of genetic variants in TLR4, TNF-α, IL10, IFN-γ, and IL37 in cytomegalovirus-positive renal allograft recipients with active CMV infection—Subanalysis of the prospective randomised VIPP study
Source: PLoS One. 2021 Apr 16;16(4):e0246118. doi: 10.1371/journal.pone.0246118 (PMC8051780; doi:10.1371/journal.pone.0246118)
Supplement: S2 Table — (DOCX) [file pone.0246118.s003.docx]

**S2 Table.**

**Genetic variants and allele and genotype frequencies of all patients and separated for the CMV prophylaxis group and the preemptive treatment group**

| **Genetic variant** | **Nucleotide change** | **Function/**  **location*** | **Total study population**  (n=116) | | | | **CMV** | |
| --- | --- | --- | --- | --- | --- | --- | --- | --- |
|  |  |  |  |  |  |  | **prophylaxis group**  (n=57) | **Preemptive group**  (n=59) |
|  |  |  | **allele** | **N (%)** | **geno-type** | **N (%)** | **N (%)** | **N (%)** |
| *TLR4*  rs4986790 | g.13843A>G | missense | A | 213 (93.4) | AA | 101 (87.1) | 49 (86.0) | 52 (88.1) |
|  |  |  | G | 15 (6.6) | AG | 11 (9.5) | 5 (8.8) | 6 (10.2) |
|  |  |  |  | | GG | 2 (1.7) | 1 (1.8) | 1 (1.7) |
|  |  |  |  |  | missing | 2 (1.7) | 2 (3.5) | 0 (0.0) |
| *TLR4*  rs4986791 | g.14143C>T | missense | C | 215 (94.3) | CC | 102 (87.9) | 49 (86.0) | 53 (89.8) |
|  |  |  | T | 13 (5.7) | CT | 11 (9.5) | 5 (8.8) | 6 (10.2) |
|  |  |  |  | | TT | 1 (0.9) | 1 (1.8) | 0 (0.0) |
|  |  |  |  |  | missing | 2 (1.7) | 2 (3.5) | 0 (0.0) |
| *TLR4*  rs5030710 | g.13262T>C | synonymous | T | 227 (98.7) | TT | 112 (96.6) | 56( 98.2) | 56 (94.9) |
|  |  |  | C | 3 (1.3) | CT | 3 (2.6) | 0 (0.0) | 3 (5.1) |
|  |  |  |  | | missing | 1 (0.9) | 1 (1.8) | 0 (0.0) |
| *TLR4*  rs7869402 | g.16573C>T | 3’UTR | C | 222 (98.2) | CC | 109 (94.0) | 54(94.7) | 55 (93.2) |
|  |  |  | T | 4 (1.8) | CT | 4 (3.4) | 1 (1.8) | 3 (5.1) |
|  |  |  |  | | missing | 3 (2.6) | 2 (3.5) | 1 (1.7) |
| *TLR4*  rs7873784 | g.17477G>C | 3’UTR | G | 198 (88.4) | GG | 88 (75.9) | 44( 77.2) | 44 (74.6) |
|  |  |  | C | 26 (11.6) | CG | 22 (19.0) | 11( 19.3) | 11 (18.6) |
|  |  |  |  | | CC | 2 (1.7) | 0 (0.0) | 2 (3.4) |
|  |  |  |  |  | missing | 4 (3.4) | 2 (3.5) | 2 (3.4) |
| *TLR4*  rs11536871 | g.9039A>C | intronic | A | 221 (96.9) | AA | 107 (92.2) | 51 (89.5) | 56 (94.9) |
|  |  |  | C | 7 (3.1) | AC | 7 (6.0) | 4 (7.0) | 3 (5.1) |
|  |  |  |  | | missing | 2 (1.7) | 2 (3.5) | 0 (0.0) |
| *TLR4*  rs11536887 | g.16215A>G | 3’UTR | A | 223 (99.6) | AA | 111 (95.7) | 53 (93.0) | 58 (98.3) |
|  |  |  | G | 1 (0.4) | AG | 1 (0.9) | 1 (1.8) | 0 (0.0) |
|  |  |  |  | | missing | 4 (3.4) | 3 (5.3) | 1 (1.7) |
| *TLR4*  rs11536889 | g.16672G>C | 3’UTR | G | 193 (83.9) | GG | 80 (69.0) | 36(63.2) | 44 (74.6) |
|  |  |  | C | 37 (16.1) | CG | 33 (28.4) | 18 (31.6) | 15 (25.4) |
|  |  |  |  | | CC | 2 (1.7) | 2 (1.8) | 0 (0.0) |
|  |  |  |  |  | missing | 1 (0.9) | 1 (3.5) | 0 (0.0) |
| *TLR4*  rs11536891 | g.17878T>C | 3’UTR | T | 199 (88.1) | TT | 88 (75.9) | 44 (77.2) | 44 (74.6) |
|  |  |  | C | 27 (11.9) | CT | 23 (19.8) | 11 (19.3) | 12 (20.3) |
|  |  |  |  | | CC | 2 (1.7) | 1 (1.8) | 1 (1.7) |
|  |  |  |  |  | missing | 3 (2.6) | 1 (1.8) | 2 (3.4) |
| *TLR4*  rs11536892 | g.18023G>A | 3’UTR | G | 115 (99.1) | GG | 114 (98.3) | 56 (98.2) | 58 (98.3) |
|  |  |  | A | 1 (0.9) | AG | 1 (0.9) | 0 (0.0) | 1 (1.7) |
|  |  |  |  | | missing | 1 (0.9) | 1 (1.8) | 0 (0.0) |
| *IFN-γ*  rs2069707 | g.4234C>G | 5’near gene | G | 215 (94.3) | GG | 103 (88.8) | 51(89.5) | 52 (88.1) |
|  |  |  | C | 13 (5.7) | GC | 9 (7.8) | 2 (3.5) | 7 (11.9) |
|  |  |  |  | | CC | 2 (1.7) | 2 (3.5) | 0 (0.0) |
|  |  |  |  |  | missing | 2 (1.7) | 2 (3.5) | 0 (0.0) |
| *IFN-γ*  rs2069723 | g.9928A>G | 3’UTR | T | 228 (100.0) | TT | 114 (98.3) | 55 (96.5) | 59 (100.0) |
|  |  |  |  | | missing | 2 (1.7) | 2 (3.5) | 0 (0.0) |
| *IFN-γ*_rs2430561 | g.6000A>T | intronic | T | 144 (63.2) | TT | 49 (42.2) | 26 (45.6) | 28 (47.5) |
|  |  |  | A | 84 (36.8) | AT | 46 (39.7) | 21 (36.8) | 20 (33.9) |
|  |  |  |  | | AA | 19 (16.4) | 10 (17.5) | 9 (15.3) |
|  |  |  |  |  | missing | 2 (1.7) | 0 (0.0) | 2 (3.4) |
| *IL10*  rs1800871 | g.4206T>C | 5’near gene | G | 176 (77.2) | GG | 72 (62.1) | 37 (64.9) | 35 (59.3) |
|  |  |  | A | 52 (22.8) | AG | 32 (27.6) | 12 (21.1) | 20 (33.9) |
|  |  |  |  | | AA | 10 (8.6) | 6 (10.5) | 4 (6.8) |
|  |  |  |  |  | missing | 2 (1.7) | 2 (3.5) | 0 (0.0) |
| *IL10*  rs1800872 | g.4433A>C | 5’near gene | G | 175 (76.1) | GG | 70 (60.3) | 36 (63.2) | 34 (57.6) |
|  |  |  | T | 55 (23.9) | TG | 35 (30.2) | 13 (22.8) | 22 (37.3) |
|  |  |  |  | | TT | 10 (8.6) | 7 (12.3) | 3 (5.1) |
|  |  |  |  |  | missing | 1 (0.9) | 1 (1.8) | 0 (0.0) |
| *IL10*  rs1800894 | g.4174G>A | 5’near gene | C | 219 (95.2) | CC | 105 (90.5) | 52 (91.2) | 53 (89.8) |
|  |  |  | T | 11 (4.8) | CT | 9 (7.8) | 4 (7.0) | 5 (8.5) |
|  |  |  |  | | TT | 1 (0.9) | 0 (0.0) | 1 (1.7) |
|  |  |  |  |  | missing | 1 (0.9) | 1 (1.8) | 0 (0.0) |
| *IL10*  rs3024489 | g.4596G>T | 5’near gene | C | 226 (100.0) | CC | 113 (97.4) | 54 (94.7) | 59 (100.0) |
|  |  |  |  | | missing | 3 (2.6) | 3 (5.3) | 0 (0.0) |
| *IL10*  rs3024496 | g.8976T>C | 3’UTR | A | 132 (58.9) | AG | 45 (38.8) | 21 (36.8) | 25 (42.4) |
|  |  |  | G | 92 (41.1) | AA | 42 (36.2) | 20 (35.1) | 21 (35.6) |
|  |  |  |  | | GG | 25 (21.6) | 12 (21.1) | 13 (22.0) |
|  |  |  |  |  | missing | 4 (3.4) | 4 (7.0) | 0 (0.0) |
| *IL10*  rs3024498 | g.9311A>G | 3’UTR | T | 181 (80.8) | TT | 71 (61.2) | 36 (63.2) | 35 (59.3) |
|  |  |  | C | 43 (19.2) | CT | 39 (33.6) | 17 (29.8) | 22 (37.3) |
|  |  |  |  | | CC | 2 (1.7) | 1 (1.8) | 1 (1.7) |
|  |  |  |  |  | missing | 4 (3.4) | 3 (5.3) | 1 (1.7) |
| *IL37*  rs2708943 | g.9162C>G | missense | C | 212 (93.0) | CC | 99 (85.3) | 51 (89.5) | 48 (81.4) |
|  |  |  | G | 16 (7.0) | CG | 14 (12.1) | 4 (7.0) | 10 (16.9) |
|  |  |  |  | | GG | 1 (0.9) | 0 (0.0) | 1 (1.7) |
|  |  |  |  |  | missing | 2 (1.7) | 2 (3.5) | 0 (0.0) |
| IL37  rs2708947 | g.10672T>C | missense | T | 215 (93.5) | TT | 100 (86.2) | 51 (89.5) | 49 (83.1) |
|  |  |  | C | 15 (6.5) | CT | 15 (12.9) | 5 (8.8) | 10 (16.9) |
|  |  |  |  | | missing | 1 (0.9) | 1 (1.8) | 0 (0.0) |
| *IL37*  rs2723171 | g.3256G>C | 5’near gene | G | 214 (93.0) | GG | 99 (85.3) | 51 (89.5) | 48 (81.4) |
|  |  |  | C | 16 (7.0) | CG | 16 (13.8) | 5 (8.8) | 11 (18.6) |
|  |  |  |  | | missing | 1 (0.9) | 1 (1.8) | 0 (0.0) |
| *IL37*  rs2723183 | g.9174A>G | missense | A | 211 (93.4) | AA | 98 (84.5) | 50 (87.7) | 48 (81.4) |
|  |  |  | G | 15 (6.6) | AG | 15 (12.9) | 5 (8.8) | 10 (16.9) |
|  |  |  |  | | missing | 3 (2.6) | 2 (3.5) | 1 (1.7) |
| *IL37*  rs2723186 | g.9533A>G | intronic | G | 221 (97.8) | GG | 109 (94.0) | 51 (89.5) | 58 (98.3) |
|  |  |  | A | 5 (2.2) | AG | 3 (2.6) | 2 (3.5) | 1 (1.7) |
|  |  |  |  | | AA | 1 (0.9) | 1 (1.8) | 0 (0.0) |
|  |  |  |  |  | missing | 3 (2.6) | 3 (5.3) | 0 (0.0) |
| *IL37*  rs2723187 | g.9722C>T | missense | C | 211 (92.5) | CC | 98 (84.5) | 50 (87.7) | 48 (81.4) |
|  |  |  | T | 17 (7.5) | CT | 15 (12.9) | 5 (8.8) | 10 (16.9) |
|  |  |  |  | | TT | 1 (0.9) | 0 (0.0) | 1 (1.7) |
|  |  |  |  |  | missing | 2 (1.7) | 2 (3.5) | 0 (0.0) |
| *IL37*  rs2723192 | g.10834G>A | missense | G | 213 (93.4) | GG | 99 (85.3) | 50 (87.7) | 49 (83.1) |
|  |  |  | A | 15 (6.6) | AG | 15 (12.9) | 5 (8.8) | 10 (16.9) |
|  |  |  |  | | missing | 2 (1.7) | 2 (3.5) | 0 (0.0) |
| *IL37*  rs3811046 | g.5831G>T | missense | T | 162 (71.1) | TT | 60 (51.7) | 31 (54.4) | 29 (49.2) |
|  |  |  | G | 66 (28.9) | GT | 42 (36.2) | 18 (31.6) | 24 (40.7) |
|  |  |  |  | | GG | 12 (10.3) | 7 (12.3) | 5 (8.5) |
|  |  |  |  |  | missing | 2 (1.7) | 1 (1.8) | 1 (1.7) |
| *IL37*  rs3811047 | g.5863A>G | missense | G | 161 (71.9) | GG | 60 (51.7) | 31 (54.4) | 29 (49.2) |
|  |  |  | A | 63 (28.1) | AG | 41 (35.3) | 17 (29.8) | 24 (40.7) |
|  |  |  |  | | AA | 11 (9.5) | 6 (10.5) | 5 (8.5) |
|  |  |  |  |  | missing | 4 (3.4) | 3 (5.3) | 1 (1.7) |
| *TNF-α*  rs1800629 | g.4682G>A | 5’near gene | G | 192 (83.5) | GG | 80 (69.0) | 39 (68.4) | 41 (69.5) |
|  |  |  | A | 38 (16.5) | AG | 32 (27.6) | 15 (26.3) | 17 (28.8) |
|  |  |  |  | | AA | 3 (2.6) | 2 (3.5) | 1 (1.7) |
|  |  |  |  |  | missing | 1 (0.9) | 1 (1.8) | 0 (0.0) |
| *TNF-α*  rs3093665 | g.7042A>C | 3’UTR | A | 208 (96.3) | AA | 100 (86.2) | 48 (84.2) | 52 (88.1) |
|  |  |  | C | 8 (3.7) | AC | 8 (6.9) | 3 (5.3) | 5 (8.5) |
|  |  |  |  | | missing | 8 (6.9) | 6 (10.5) | 2 (3.4) |

^*^Function/location based on transcript variants NM_138554 (TLR4), NM_000619 (IFN-γ), NM_000572 (IL10), NM_014439 (IL37) and NM_000594 (TNF-α). A, alanine; C, cytosine; G, guanosine; T, thymidine; UTR, untranslated region.
