# Supplementary material for: No association of genetic variants in TLR4, TNF-α, IL10, IFN-γ, and IL37 in cytomegalovirus-positive renal allograft recipients with active CMV infection—Subanalysis of the prospective randomised VIPP study
Source: PLoS One. 2021 Apr 16;16(4):e0246118. doi: 10.1371/journal.pone.0246118 (PMC8051780; doi:10.1371/journal.pone.0246118)
Supplement: S1 File — (PDF) [file pone.0246118.s004.pdf]

AMENDMENT TO CLINICAL STUDY PROTOCOL

STUDY ID: ML 19313

EudraCT-No.: 2005-004695-20

Randomized multicenter trial comparing Valganciclovir CMV prophylaxis versus pre-emptive therapy after renal transplantation using proteomics for monitoring of graft alteration

Roche

Roche Pharma AG

Amendment No.3, **Version** *1.1* **dated** *09.08.2010*

to the Study Protocol, **Version** *4.2* **dated** *11.04.2008*

CONFIDENTIALITY STATEMENT

The information contained in this document is the property of Roche Pharma AG and therefore is provided to you in confidence for review by you, your staff, an applicable Ethics Committee/Institutional Review and regulatory authorities. It is understood that the information will not be disclosed to others without prior written approval from Roche Pharma AG, except to the extent necessary to obtain informed consent from those persons to whom the medication may be administered.

Table of Content

1 Rationale for Amendment 2
2 Amended Sections 2
2.1 All Sections 2
2.2 Responsibilities 2
2.3 Flow Chart and Glossary of Abbreviations 2
2.4 Synopsis 3
2.5 Study Protocol Section 3.5 Secondary Basic Research Projects (SBRPs) 7
2.6 Study Protocol Section 5.2.2.4 Urine sample for proteomics (S2) 7
2.7 Study Protocol Section 5.2.7.3 Year 2 to 7 Post Transplant 7
2.8 Study Protocol Section 8.4.1 For Subjects 8
2.9 Study Protocol Section 19.5 Appendix 5 Secondary Basic Research Project 1 (SBRP 1) 8

1 Rationale for Amendment

The following summarizes the rationale behind this amendment and the changes to the study protocol that are required to be made as a result:

- *Changes in study procedures*
  - *For dropped out patients, survival of the transplant and the patient can be followed up by telephone contact, if patients give consent according to german data protection law. This will avoid censored data in the graft survival analysis and thus enhance the quality of the analysis.*
  - *Sample for Proteomics will be taken only until month 24*
- *Removal of one Secondary Basic Research Project*
- *Correction of spelling and formatting mistakes*
- *Personnel Changes*

2 Amended Sections

The following sections will be amended

2.1 All Sections

Minor spelling and formatting mistakes were corrected.

2.2 Responsibilities

Prof. Stephan Korom and Matthias Wahl have taken over responsibility as medical manager and Dr. Dorothea Thurn has taken over responsibility as drug safety specialist.

The CRO IFE GmbH has been taken over by Pierrel Research and thus the name changed, however responsibilities remain unchanged.

2.3 Flow Chart and Glossary of Abbreviations

Due to the necessary changes the Flow Chart and the Glossary of Abbreviations had to be adapted as well. As these changes are described in the respective section of this amendment, no details are provided for the changes in the Flow Chart and the Glossary of Abbreviations.

2.4 Synopsis

Original text:

IN CASE OF EMERGENCY

In case of emergency one of the following persons must be contacted immediately. In the case of a serious adverse event (SAE) or pregnancy the CRO/Monitor must be contacted within one working day by fax as indicated below:

**CRO/Monitor**	Dr. Andree Beckerling
	IFE Europe GmbH
Address:	Z. Katharina 6
	45703 Essen
Phone:	+49-(0)201-8990-0
Fax:	+49-(0)201-8990-201
**Medical Manager Roche Grenzach**	Dr. Steffen Pelzl
Address:	Roche Pharma AG
	D-79630 Grenzach-Wyhlen
Phone:	+49 (0)7624 143287
Fax:	+49 (0)7624 142353

If required drug safety should be informed.

**Drug safety specialist**	Dr. Hans-Peter Stobernack
Address:	Roche Pharma AG
	D-79630 Grenzach-Wyhlen
Phone:	+49 (0)7624 142423
Fax:	+49 (0)7624 143183

Amended text:

Note: *Since no patient in the VIPP study receives IMP any more and all patients are in the follow-up phase of the study, the drug labels will not be adapted to reflect the name change of the CRO.*

IN CASE OF EMERGENCY

In case of emergency one of the following persons must be contacted immediately. In the case of a serious adverse event (SAE) or pregnancy the CRO/Monitor must be contacted within one working day by fax as indicated below:

**CRO/Monitor**	Dr. Andree Beckerling
	Pierrel Research Europe GmbH
Address:	Z. Katharina 6
	45703 Essen
Phone:	+49-(0)201-8990-0
Fax:	+49-(0)201-8990-201
**Medical Manager Roche Grenzach**	Prof. Stephan Korom
Address:	Roche Pharma AG
	D-79630 Grenzach-Wyhlen
Phone:	+49 (0)7624 143668
Fax:	+49 (0)7624 143143
**Medical Manager Roche Grenzach**	Matthias Wahl
Address:	Roche Pharma AG
	D-79630 Grenzach-Wyhlen
Phone:	+49 (0)7624 142142
Fax:	+49 (0)7624 143143

If required drug safety should be informed.

**Drug safety specialist**	Dr. Dorothea Thurn
Address:	Roche Pharma AG
	D-79630 Grenzach-Wyhlen
Phone:	+49 (0)7624 143707
Fax:	+49 (0)7624 143183

Original text:

RESPONSIBILITIES

**Sponsor**	Roche Pharma AG
Contact Person:	Dr. Steffen Pelzl
Address:	Emil-Barell Str. 1
	D-79639 Grenzach-Wyhlen
	Germany
Phone:	+49 (0)7624 143287
Fax:	+49 (0)7624 142353
E-mail:	steffen.pelzl@roche.com

**Coordinating Investigator**	
**(Leiter der klinischen Prüfung)**	Privatdozent Dr. med. Volker Kliem
Address:	Nephrologisches Zentrum Niedersachsen
	Am Vogelsang 105
	D-34346 Hann. Münden
	Germany
Phone:	+49 (0)5541 996-328
Fax:	+49 (0) 5541 996-391
E-mail:	v.kliem@nzn.de

**CRO**	IFE Europe GmbH
Contact Person:	Dr. Andree Beckerling
Address:	Z. Katharina 6
	D-45703 Essen
	Germany
Phone:	+49-(0)201-8990-0
Fax:	+49-(0)201-8990-101
E-mail:	office@ife-europe.com

Amended text:

RESPONSIBILITIES

**Sponsor**	Roche Pharma AG
Contact Person:	Matthias Wahl
Address:	Emil-Barell Str. 1
	D-79639 Grenzach-Wyhlen
	Germany
Phone:	+49 (0)7624 142142
Fax:	+49 (0)7624 143143
E-mail:	Matthias.wahl@roche.com

**Coordinating Investigator**	
**(Leiter der klinischen Prüfung)**	Prof. Dr. med. Volker Kliem
Address:	Nephrologisches Zentrum Niedersachsen
	Am Vogelsang 105
	D-34346 Hann. Münden
	Germany
Phone:	+49 (0)5541 996-328
Fax:	+49 (0) 5541 996-391
E-mail:	v.kliem@nzn.de

**CRO**	Pierrel Research
Contact Person:	Dr. Andree Beckerling
Address:	Z. Katharina 6
	D-45703 Essen
	Germany
Phone:	+49-(0)201-8990-0
Fax:	+49-(0)201-8990-101
E-mail:	office@pierrel-research.com

2.5 Study Protocol Section 3.5 Secondary Basic Research Projects (SBRPs)

Original text:

Two secondary basic research projects (SBRP 1 and SBRP 2) will be conducted in conjunction with the main protocol for study ML 19313. All patients enrolled for the main protocol ML19313 are eligible for enrolment in the SBRPs. Participation in the SBRPs is entirely voluntary. A detailed study protocol for SBRP 1 is given in Appendix 5 and for SBRP 2 in Appendix 6.

Amended text:

One secondary basic research projects (SBRP 2) will be conducted in conjunction with the main protocol for study ML 19313. All patients enrolled for the main protocol ML19313 are eligible for enrolment in the SBRP. Participation in the SBRP is entirely voluntary. A detailed study protocol for the SBRP is given in Appendix 5.

2.6 Study Protocol Section 5.2.2.4 Urine sample for proteomics (S2)

Original text:

2 ml urine sample for proteomics will be collected and stored at -20°C at each visit. .

Amended text:

2 ml urine sample for proteomics will be collected and stored at -20°C at each visit. The last sample taken for proteomics will be at month 24. After that visit no further samples for proteomics will be taken.

2.7 Study Protocol Section 5.2.7.3 Year 2 to 7 Post Transplant

Original text:

During year 2 to 7 post transplant full assessments of the patient at the transplant center are required as follows in six monthly intervals (Visits 16-27):

- Graft assessment (R2)
- Assessments of CMV Disease (S2)
- Vital signs (R2) / Actual body weight (R2)
- Adverse events (R2, only if study drug related)
- Concomitant medication (R2)
- Serum creatinine / Creatinine clearance (calculated) (R[2])
- Urine sample for proteomics (S[2])

- Blood sample for Cobas® Amplicor® CMV MONITOR (quantitative PCR) (S^2)
- Drug monitoring immunosuppressive drugs (R^2)
- Urinary Status

Amended text:

During year 2 to 7 post transplant full assessments of the patient at the transplant center are required as follows in six monthly intervals (Visits 16-27):

- Graft assessment (R^2)
- Assessments of CMV Disease (S^2)
- Vital signs (R^2) / Actual body weight (R^2)
- Adverse events (R^2, only if study drug related)
- Concomitant medication (R^2)
- Serum creatinine / Creatinine clearance (calculated) (R^2)
- Urine sample for proteomics (S^2) until Visit 24
- Blood sample for Cobas® Amplicor® CMV MONITOR (quantitative PCR) (S^2)
- Drug monitoring immunosuppressive drugs (R^2)
- Urinary Status

2.8 Study Protocol Section 8.4.1 For Subjects

Original text:

No subject prematurely discontinued from the study for any reason will be replaced..

Amended text:

No subject prematurely discontinued from the study for any reason will be replaced, however subjects who dropped out of the study will be asked to provide information about their survival, their graft survival and their immunsupressive medication in a non-interventional fashion by telephone to avoid a large amount of censored data in the survival analysis.
Patients will have to give a separate consent according to german data protection law (Bundesdatenschutzgesetz) to provide this information to Roche Pharma AG.

2.9 Study Protocol Section 19.5 Appendix 5 Secondary Basic Research Project 1 (SBRP 1)

Original text:

1. Objectives and Background of the Research Project

The primary objective of this research project is to obtain 3 blood samples (20 milliliter each) from patients enrolled in the associated protocol ML 19313 for the purpose of investigating CMV-IE-1 T cell memory immune responses by measuring the proportion of interferon γ-producing T cells following ex vivo activation with pools of overlapping peptides representing the immediate early (IE)-1 proteins as

described by Bunde et al. in renal allograft recipients IgG seropositive for CMV at risk for active CMV infection and/or CMV disease during the first year after transplantation.
Cytomegalovirus (CMV) remains the most important serious infection complicating solid organ transplantation (SOT). CMV infection can result in CMV disease, which, in severe cases, can lead to hospitalization, morbidity and in some cases, death. Valganciclovir a valyl ester prodrug of ganciclovir, is currently licensed for the induction and maintenance therapy of CMV retinitis in Acquired Immune Deficiency Syndrome (AIDS) patients and for the prevention of CMV disease in high risk (donor CMV positive / recipient CMV negative) patients in SOT.
T cell immunity is essential to control latent CMV infection [1,2]. In BALB/c mice the immediate early (IE) protein pp89 was found to be an important target protein for cytotoxic T cells (CTL). CTL responses against pp89 were shown to mediate protection against CMV [3,4]. In humans, IE-1-specific CD8+ T cells were detected at frequencies comparable to those of pp65 specific CD8+ T cells [5]. To date both pp65 and IE-1 are considered to represent dominant T cell targets [5]. Measured by ex vivo stimulation with peptide pools representing all possible CD4 and CD8 T cell epitopes for pp65 and IE-1 a recent study in 27 heart and lung transplant recipients evaluated the role of Interferon-γ (IFN- γ) producing T cell frequencies in preventing active CMV infection and disease [6]. Remarkably, the authors could demonstrate that dominance and magnitude of the IE-1 but not the pp65 specific CD8+ T cell response correlated with protection from CMV disease [6]. Another recent study by the same group in 64 renal allograft recipients showed a correlation of the IE-1 specific IFN- γ producing T cell response monitored during 6 months post transplant with improved renal function as well as an inverse correlation between IE-1 specific and alloreactive IFN- γ producing T cells at certain time points after transplantation [7]. These data suggest that IE-1 specific T cell frequencies may be crucial for anti CMV immunity as well as renal allograft function possibly by preventing the CMV induced generation of alloreactive T cells. The immunological data gathered through the analysis of samples in the SBRP 1 is hoped to improve graft and patient survival in renal allograft recipients IgG seropositive for CMV at risk for active CMV infection and/or CMV disease by:

- Indicating the kinetics of CMV-IE-1 T memory cells during the first year after renal transplantation.
- Indicating the impact of the two CMV prevention concepts (valganciclovir prophylaxis vs. preemptive therapy with valganciclovir) on the development of CMV-IE-1 memory T cells during the first year after renal transplantation.
- Indicating the impact of CMV-IE-1 memory T cells on the occurence of active CMV infections and/or CMV disease in renal allograft recipients during the first year after renal transplantation.
- Indicating the impact of CMV-IE-1 memory T cells kinetics on longterm renal allograft survival.

The SBRP 1 will be conducted in compliance with the protocol, Good Clinical Practice (ICH GCP) and any applicable regulatory requirements.

2. Design

This SBRP 1 protocol may be conducted in conjunction with the main protocol for study ML 19313.

ML 19313 is a randomized multicenter trial comparing Valganciclovir CMV prophylaxis versus pre-emptive therapy after renal transplantation using proteomics for monitoring of graft alteration. The primary objective of this trial is to compare in CMV positive renal allograft recipients the efficacy of valganciclovir (900 mg once daily) prophylaxis applied until Day 100 post transplant with no prophylaxis under the condition of pre-emptive therapy of active CMV infection. The secondary objective of this trial is to investigate the influence of both CMV prevention concepts on the occurrence of direct and indirect effects of active CMV infections: CMV disease (direct); renal function, rejections, opportunistic infections, graft- and patient survival, diabetes (indirect effects). In addition the safety of both concepts will be investigated by evaluating side effects, leucopenia/neutropenia, and infections.

3. Population

3.1 Target Population

All patients enrolled in the main protocol ML 19313 are eligible for enrolment in the SBRP 1. It is expected that approximately 100 patients may participate.
Participation in this SBRP 1 protocol is entirely voluntary.

3.2 Inclusion Criteria

For inclusion into the SBRP 1, a patient must satisfy the following criteria:

- Written informed consent previously obtained for the main protocol ML 19313
- Enrolment in main protocol ML 19313
- Written informed consent for the SBRP 1

3.3 Exclusion Criteria

There are no exclusion criteria for this research project.

4. Schedule of Assessments and Procedures

4.1 Procedures / Assessments

After written informed consent for associated clinical study ML 19313 has been obtained from a patient, the SBRP 1 and blood sampling procedure will be explained. The patient will be asked if he/she wishes to participate and written informed consent may be obtained specifically for the SBRP 1.

The SBRP 1 blood samples may be obtained at the same time as blood sampling in the clinical protocol ML 19313 to avoid repeated venepuncture.

The SBRP 1 samples should be collected as shown in the Table below.

Sample	Visit of study ML19313	Days post transplantation
1	9	100
2	13	196
3	15	364

Dates of consent and blood sample collection should be recorded on the associated SBRP 1 page of the eCRF.

4.2 Sampling Procedures

Study sites will be supplied with kits containing six plastic 10 ml S-Monovette Citrate tubes for sample collection, referring needles and adapters as well as patient specific labels containing the same individual patient number as used in the associated clinical study ML 19313. Once informed consent is obtained, 20 ml of blood should be collected by venepuncture into two 10 ml Citrate blood collection tubes at every visit as indicated in the Table shown in section 4.1. The blood samples should be carefully mixed with the anticoagulant in the tubes by inverting the tubes several times. The patient individual label as mentioned above should be placed on the 10 ml tubes. At the study sites peripheral mononuclear cells (PBMC) may be separated from the obtained blood samples by use of a Ficoll-Paque density barrier. Isolated PBMC will be resolved in RPMI containing antibiotics with additional L-glutamine and fetal bovine serum and the absolute amount of viable cells may be determined by trypan blue staining. The concentration of the final cell suspension should be adjusted to $1x10^7$ cells per milliliter. Subsequently, the isolated PBMC will be transferred by carrier from the study sites to the Sample Repository 1 (SR 1) located at Medizinische Klinik mit Schwerpunkt Nephrologie und Intern. Intensivmedizin; Charité - Universitätsmedizin Berlin; Campus Virchow-Klinikum; Augustenburger Platz 1; 13353 Berlin. At the site the cell suspension will be transferred to Cryotubes labeled with the same individual patient number as used for the patient in the associated study ML 19313 and finally forwarded to the SR 1 for storage and cellular analysis. Cellular samples as well as the obtained immunological information will be captured on the corresponding SBRP 1 page in the eCRF of the associated study ML 19313. At the end of the associated clinical study ML 19313, clinical data from ML 19313 will be linked to immunological information and analyzed as indicated in chapter 5. No more than 15 years after the end of the associated study (database closure), all blood and cellular samples will be destroyed.

5. Statistical Analysis

In order to compare T cell frequencies in the relevant patient groups a Mann-Whitney U test will be done. Bivariate Pearson correlation of CMV-specific T cells with renal function parameters will be used in the relevant subgroups. To evaluate the influence of multiple parameters (IE-1-specific T cells, CMV viral load, CMV infection, CMV serostatus, CMV Disease, acute rejection, delayed graft function, donor age, recipient age) on renal allograft function at 6 and 12 months post transplant a Multivariate linear regression model will be performed.

6. Withdrawal of Patients from the SBRP 1

Patients have the right to withdraw their informed consent for the SBRP 1 as well as their samples from the Sample Repository 1 (SR 1) located at Medizinische Klinik mit Schwerpunkt Nephrologie und Intern. Intensivmedizin; Charité - Universitätsmedizin Berlin; Campus Virchow-Klinikum; Augustenburger Platz 1; 13353 Berlin, at any time for any reason during their participation in the clinical study to which this SBRP 1 is associated (ML 19313). Withdrawal of informed consent for SBRP 1 will not affect the participation of the patient in the associated study ML 19313.
If a patient wishes to withdraw his/her samples, the investigator must inform the Roche monitor and enter the date of withdrawal in the patient´s Case Report Form (CRF). Within Roche, the request for sample withdrawal will be forwarded to the SBRP 1 project leader. If the samples are already at the Sample Repository 1 in Berlin, the SBRP 1 project leader will issue confirmation of the withdrawal, which will be forwarded to the investigator. If the samples are still at the investigator site at the time a patient wishes to withdraw his/her samples, the investigator must inform the Roche monitor as before, destroy the samples and sign the SBRP 1 Patient Withdrawal Form to confirm that this has been done. The Roche monitor will forward confirmation of destruction, recorded on the Patient Withdrawal Form, to the SBRP 1 project leader. Data obtained before withdrawal of participation will be included in the final statistical analysis of the SBRP 1.

7. Access to Source Documents

Roche monitors and auditors will have direct access to appropriate parts of records relating to patients participating in this study for the purposes of verifying the data provided to Roche. The sites will permit monitoring, audits, IRB/IEC review, and regulatory inspections by providing direct access to source data and documents related to the SBRP 1.

8. Ethical Aspects

Please refer to Section 12. “Ethical Aspects” in the protocol for study ML 19313.

9. Study Documentation, CRF and Record Keeping

Please refer to Section 15. “Study Documentation, CRFs and record keeping” in the protocol for study ML 19313.

10. Confidentiality of Study Documents and Patient Records

Delivery, storage, processing and measurement of the SBRP 1 samples as well as the obtained immunological data will be documented in a defined SBRP 1 chapter of the eCRF of the associated study ML 19313. The SBRP 1 chapter will not be linked with other chapters of the eCRF of ML 19313 to guarantee that only at the end of the associated clinical study ML 19313 clinical data from ML 19313 can be linked with immunological data obtained in the SBRP 1. The documentation of data for the SBRP 1 will be done by a study nurse employed at Medizinische Klinik mit Schwerpunkt Nephrologie und Intern. Intensivmedizin; Charité - Universitätsmedizin Berlin; Campus Virchow-Klinikum; Augustenburger Platz 1; 13353 Berlin who will only have access to the SBRP 1 chapter of the eCRF and who will not be involved in the documentation of patient specific data for the associated clinical study ML 19313. Investigators as well as study nurses of the associated clinical study ML 19313 will not have access to the SBRP 1 chapter of the eCRF. Additionally investigators as well as patients participating in the associated clinical trial ML 19313 will not be provided with data generated in the SBRP 1.

For further details please refer to Section 17. “Confidentiality of trial documents and subject records” in the protocol for study ML 19313.

11. Publication of Data

Please refer to Section 18. “Publication of Data” in the protocol for study ML 19313.

12. References

1. Fishman JA, Rubin RH. Infection in organ-transplant recipients. N Engl J Med 1998; 338:1741-1751

2. Quinnan GV Jr, Kirmani N, Rook AH, Manischewitz JF, Jackson L, Moreschi G, Santos GW, Saral R, Burns WH. Cytotoxic t cells in cytomegalovirus infection: HLA-restricted T-lymphocyte and non-T-lymphocyte cytotoxic responses correlate with recovery from cytomegalovirus infection in bone-marrow-transplant recipients. N Engl J Med 1982; 307:7-13

3. Jonjić S, del Val M, Keil GM, Reddehase MJ, Koszinowski UH. A nonstructural viral protein expressed by a recombinant vaccinia virus protects against lethal cytomegalovirus infection. J Virol 1988; 62:1653-1658

4. Reddehase MJ. Antigens and immunoevasins: opponents in cytomegalovirus immune surveillance. Nat Rev Immunol 2002; 2:831-844

5. Kern F, Surel IP, Faulhaber N, Frömmel C, Schneider-Mergener J, Schönemann C, Reinke P, Volk HD. Target structures of the CD8(+)-T-cell response to human cytomegalovirus: the 72-kilodalton major immediate-early protein revisited. J Virol 1999; 73:8179-8184

6. Bunde T, Kirchner A, Hoffmeister B, Habedank D, Hetzer R, Cherepnev G, Proesch S, Reinke P, Volk HD, Lehmkuhl H, Kern F. Protection from cytomegalovirus after transplantation is correlated with immediate early 1-specific CD8 T cells. J Exp Med 2005; 201:1031-1036

7. Nickel P, Bold G, Presber F. Frequencies of CMV-IE specific memory T cells are inversely correlated with alloimmune memory and serum creatinine in kidney transplant patients. Manuscript in preparation

13. SBRP 1 Project Leader

Prof. Dr. med. Petra Reinke
Medizinische Klinik mit Schwerpunkt
Nephrologie und Intern. Intensivmedizin
Charité - Universitätsmedizin Berlin
Campus Virchow-Klinikum
Augustenburger Platz 1
13353 Berlin

Telephone Number: 0049 30 553 232
Fax Number: 0049 30 553 916
Email: petra.reinke@charite.de

Amended text:

Note: No data has been collected in this project

This section Appendix 5 (SBRP1) is deleted.

PROTOCOL SIGNATURE PAGE

Protocol No.: ML19313

Randomized multicenter trial comparing Valganciclovir CMV prophylaxis versus pre-emptive therapy after renal transplantation using proteomics for monitoring of graft alteration

Protocol Date: 09.08.2010

Protocol Version: 4.3

EudraCT-Nr.: 2005-004695-20

I have thoroughly read and reviewed the above mentioned study protocol. Having read and understood the requirements and conditions of the study protocol, I agree to perform the clinical study according to the international good clinical practice principles, the applicable regulations and laws and regulatory authority requirements.

	Date	Signature
Medical Manager, Roche Pharma AG<br>Matthias Wahl	11-AUG-10	Matthias Wahl
Statistician, Pierrel Research GmbH<br>Waldemar Braun	13-AUG-2010	W. Braun
CRO Project Manager, Pierrel Research GmbH<br>Dr. Andree Beckerling	23. Aug. 2010	[illegible]
Coordinating Investigator<br>(Leiter der klinischen Prüfung, LKP)<br>Prof. Dr. med. Volker Kliem	04/09/2010	Kliem

CENTRE SPECIFIC PROTOCOL SIGNATURE PAGE

col No.:	ML19313
	Valcyte infection prophylaxis and proteomics
col Date:	09.08.2010
col Version:	4.3
aCT-Nr.:	2005-004695-20

N-Nr.: 67448

e of Responsible Trial Site Investigator: Professor Dr. med. Volker Kliem

tigational Site (Name of Institute, Address): NEPHROLOGISCHES ZENTRUM NIEDERSACHSEN VOGELSANG 105 34346 HANN. MUENDEN

e thoroughly read and reviewed the above mentioned study protocol. Having read and understood the rements and conditions of the study protocol, I agree to perform the clinical study according to the national good clinical practice principles, the applicable regulations and laws and regulatory authority rements.

Name	Function *(Principal Investigator, Co-Investigator, Study Nurse, Study Coordinator)*	Date	Signature
Kliem	LKP	04/09/2010	[signature]

CLINICAL STUDY PROTOCOL

Protocol No.: ML 19313

Randomized multicenter trial comparing Valganciclovir CMV prophylaxis versus pre-emptive therapy after renal transplantation using proteomics for monitoring of graft alteration

Roche Pharma AG

Date of the Protocol: 09.08.2010 Protocol Version: 4.3

EudraCT No.: 2005-004695-20

CONFIDENTIALITY STATEMENT

The information contained in this document is the property of Roche Pharma AG and therefore is provided to you in confidence for review by you, your staff, an applicable Ethics Committee/Institutional Review and regulatory authorities. It is understood that the information will not be disclosed to others without prior written approval from Roche Pharma AG, except to the extent necessary to obtain informed consent from those persons to whom the medication may be administered.

SYNOPSIS

Protocol No.	ML 19313
Protocol Version/Date	4.3/09.08.2010
Title	Randomized multicenter trial comparing Valganciclovir CMV prophylaxis versus pre-emptive therapy after renal transplantation using proteomics for monitoring of graft alteration
EudraCT No.	2005-004695-20
Sponsor	Roche Pharma AG
Project phase	III
Indication	Prevention of CMV disease in CMV seropositive kidney transplant recipients
Objectives	
Primary objective	The primary objective of this trial is to compare in CMV positive renal allograft recipients the efficacy of valganciclovir (900 mg once daily) prophylaxis applied until Day 100 post transplant with no prophylaxis under the condition of pre-emptive therapy of active CMV infection.<br>For the corresponding primary endpoints please refer to section main parameters in this synopsis.
Secondary objectives	The secondary objective of this trial is to investigate the influence of both CMV prevention concepts on the occurrence of direct and indirect effects of active CMV infections: CMV disease (direct); renal function, rejections, opportunistic infections, graft- and patient survival, diabetes (indirect effects). In addition the safety of both concepts will be investigated by evaluating side effects, leucopenia/neutropenia, and infections.<br>For description of the corresponding secondary endpoints please refer to section main parameters in this synopsis.
Trial design	Open label randomized comparative study with two parallel groups
Target population	Kidney transplant recipients

2001-06-MD2.2_01

Planned sample size	300 patients
Inclusion/Exclusion criteria	*Inclusion criteria*

1. Patient has received within the preceding 14 days a primary or secondary renal allograft from a living or cadaveric donor
2. Patient is IgG seropositive for CMV and has received an allograft from a CMV IgG seropositive or seronegative donor
3. Patient receives immunosuppression including a CNI (CsA or tacrolimus) and MMF (mycophenolate mofetil)
4. Patient is 18 years of age or older
5. Patient is willing to give written informed consent, written consent for data protection and willingness to participate and to comply with the study
6. Laboratory parameters: Patient has adequate hematological and renal function defined as:
   a) Leucocyte count >3,500 cells/µL
   b) Platelet count >100,000 cells/µL
   c) Hemoglobin >8.0 g/dL
   d) Estimated creatinine clearance (calculated by the Cockcroft-Gault formula, see Section 6.1.2) of >10 ml/min with evidence of improving renal function
7. Patient agrees to utilize contraceptive methods throughout the study period and for 90 days following discontinuation of the Study Drug. Male patients must agree to use condoms throughout the study period and for 90 days following discontinuation of the Study Drug.
8. Females of childbearing potential will have a negative pregnancy test at screening
9. Patient is able to tolerate oral medication within 14 days post transplantation. The day of completion of transplant surgery is defined as Day 0 post transplantation.

Exclusion criteria (orientating)

1. Patient has active CMV infection (CMV PCR $\geq$ 400 copies/ml)
2. Severe uncontrolled diarrhea or evidence of malabsorbtion
3. Patients with malignancies or history of malignancy except non metastatic basal or squamous cell carcinoma of the skin that has been treated successfully
4. Acute steroid resistant rejection episode since transplantation

2001-06-MD2.2_01

Total number of centers<br>Sample size per center	Approximately 25 centers in Germany and Austria.<br>At least 10 patients per site.
Length of study	First patient in: Q2 2006<br>Last patient in: Q4 2008<br>First patient out: Q2 2013<br>Last patient out Q4 2015<br>Individual patient study duration:<br>12 months study phase, 72 months follow up
Investigational medicinal product(s)<br>(Dose/Route/Regimen)	Valganciclovir 450 mg tablet, 1 x 2 tablets (900mg) per day adjusted to renal function applied until Day 100 post transplant<br>In case of active CMV infection Valganciclovir 2 x 2 tablets (1800mg) per day adjusted to renal function followed by secondary prophylaxis with 1 x 2 tablets (900mg) per day adjusted to renal function for 28 days.
Comparator drug(s)<br>(Dose/Route/Regimen)	Not applicable
Background medication<br>(Dose/Route/Regimen)	Immunosuppression with a calcineurin inhibitor (cyclosporine A or tacrolimus), mycophenolate mofetil and steroids, dosing according to center practice
Main parameter(s) of<br>Efficacy	The primary objective will be evaluated by assessment of the following parameters:<br>• Proportion of patients with active CMV infection within 12 months (plasma PCR ≥ 400 copies/ml).<br>• Proportion of patients with CMV disease within 12 months including CMV syndrome and tissue invasive disease.<br>• Urine proteomic pattern at month 12 on a scale between –1 = no graft alteration and +1 = graft alteration.<br>• Time to graft loss.<br>*Definitions*:<br><u>*Active CMV infection*</u> is defined as viremia according plasma PCR ≥ 400 copies/ml.<br><u>*CMV disease*</u> includes CMV syndrome and CMV tissue invasive disease defined as follows:

2001-06-MD2.2_01

CMV Syndrome is defined as viremia according plasma PCR ≥ 400 copies/ml and at least one of the following signs: Fever of ≥38°C; new or increased malaise (malaise defined as normal activity reduced >50%; cannot work [Grade 3 Toxicity] or unable to care for self [Grade 4 Toxicity]); leucopenia on 2 successive measurements separated by at least 24 hours (defined as (1) a WBC of <3,500/µL or (2) a WBC decrease of >20% if the WBC prior to development of viremia is <4,000/µL); atypical lymphocytosis of ≥5%; thrombocytopenia (defined as (1) a platelet count of <100,000/µL or (2) a decrease of >20% if the platelet count prior to development of viremia is <115,000/µL); elevation of hepatic transaminases (ALT or AST) to at least 2xULN.

CMV tissue invasive disease defined as viremia according plasma PCR ≥ 400 copies/ml and clinical evidence of localized CMV infection (CMV inclusion cells or in situ detection of CMV antigen or DNA by immunostain or hybridization, respectively) in a biopsy or other appropriate specimen (e.g., Bronchoalveolar Lavage (BAL), Cerebal Spinal Fluid (CSF)) and / or relevant symptoms or signs of organ dysfunction.

Secondary parameters:

1.a Proportion of patients with CMV syndrome within 12 months
1.b Proportion of patients with CMV tissue invasive disease within 12 months
2. Time to occurrence of first viremia (plasma PCR ≥ 400 copies/ml)
3. Viral burden at viremia (AUC of plasma PCR)
4. Creatinine clearance at month 12 (estimated by Cockroft-Gault formula as well as by MDRD formula, see 6.1.2)
5. Proportion of patients with treated and biopsy proven acute rejection episodes within 12 months
6. Cost survey including medication, monitoring, hospitalization
7. Correlation of proteomics pattern with graft survival
8. Correlation of proteomics pattern with patient survival
9. Predictive value of proteomics pattern for graft survival
10. Predictive value of proteomics pattern for patient survival

2001-06-MD2.2_01

	11. Hematological parameters including incidence of leucopenia (WBC of <3,500/µL and <1,000/µL) and neutropenia (ANC <750/µL) within 12 months<br>12. Proportion of patients with opportunistic infections within 12 months (bacterial, fungal, non CMV viral)<br>13. Patient survival at month 12<br>14. Graft survival at month 12<br>15. Proportion of patients with post transplant diabetes mellitus according to fasting glucose level or OGTT month 6 and month 12<br>16. Incidence of active CMV infection not responding to valganciclovir or IV ganciclovir treatment
Follow up phase	17. Proportion of patients with CMV disease from baseline to month 18 and every 6 months up to months 84<br>18. Proportion of patients with CMV viremia from baseline to month 18 and every 6 months up to months 84<br>19. Proportion of patients with CMV syndrome from baseline to month 18 and every 6 months up to months 84<br>20. Proportion of patients with CMV tissue invasive disease from baseline to month 18 and every 6 months up to months 84<br>21. Proportion of patients who exhibit a specific urine proteomic pattern at month 18 and every 6 months up to months 84<br>22. Patient survival at month 18 and every 6 months up to months 84<br>23. Graft survival at month 18 and every 6 months up to months 84
Study procedure	Group A:<br>Oral CMV prophylaxis with 1x 900 mg valganciclovir per day adjusted to renal function starting within 14 days of transplantation until Day 100 post transplant. In case of active CMV infection pre-emptive therapy as described below.<br><br>Group B:<br>No oral primary prophylaxis, only pre-emptive therapy as described below:<br><br>All patients are closely monitored for CMV infection in plasma samples by quantitative PCR with Cobas® Amplicor® CMV Monitor. When plasma PCR ≥ 400 copies/ml, pre-emptive therapy is initiated with 2x 900 mg Valganciclovir per day adjusted to renal func-

	tion for at least 14 days until second negative PCR (below 400 copies/ml) followed by secondary prophylaxis for 28 days with 1x 900 mg valganciclovir adjusted to renal function. In case of CMV disease or no response to Valganciclovir treatment after 14 days (not falling viral load) IV ganciclovir or additional appropriate therapy can be administered according to the local site's standard instead of valganciclovir. Urine samples for proteomic analysis will be drawn at every visit.
Randomization procedure	Central randomization stratified by center and by induction immunosuppression with polyclonal antibodies, such as ATG, ALG or OKT® 3
Statistical considerations	
Sample size calculation	The third step of the hierarchic test procedure has lowest power in the hierarchy. Therefore, the following sample size calculation is based on the third step of the hierarchic test procedure within the first hypotheses system assuming a graft loss rate over a period of 48 months of 10% for patients with Valganciclovir CMV prophylaxis and 25% for patients with pre-emptive therapy:<br><br>A Fisher's exact test with a 0.040 two-sided significance level will have 90% power to detect the difference between a Group 1 proportion, π_1, of 0.100 and a Group 2 proportion, π_2, of 0.250 when the sample size in each group is 150.
Analysis plan	The primary objective will be evaluated by evaluating of the following parameters based on the ITT population:<br>• Proportion of patients with active CMV infection within 12 months (plasma PCR $\geq$ 400 copies/ml).<br>• Proportion of patients with CMV disease within 12 months including CMV syndrome and tissue invasive disease.<br>• Urine proteomic pattern at month 12 on a scale between -1 = no graft alteration and $+1$ = graft alteration.<br>• Time to graft loss.<br><br>The following two hypotheses systems will be tested simultaneously based on a global type I error of $\alpha = 5\%$ using a correction with type I errors of

α_1= 4% and α_2= 1%:

The first hypotheses system will be carried out in a hierarchic test procedure with an α_1-level of 4%. Therefore the following three hypotheses systems will be tested hierarchically:

H_{011}: There is no difference between the two treatment groups in the proportion of patients with active CMV infection within 12 months.

H_{111}: There is a difference between the two treatment groups in the proportion of patients with active CMV infection within 12 months.

In case of a non-significant result for the hypothesis H_{011}, the hierarchic test procedure terminates. In case of a significant result the hypothesis H_{012} can be tested.

H_{012}: There is no difference between the two treatment groups in the proportion of patients with CMV disease within 12 months including CMV syndrome and tissue invasive disease.

H_{112}: There is a difference between the two treatment groups in the proportion of patients with CMV disease within 12 months including CMV syndrome and tissue invasive disease.

In case of a non-significant result for the hypothesis H_{012}, the hierarchic test procedure terminates. In case of a significant result the hypothesis H_{013} can be tested.

H_{013}: There is no influence of treatment on the time to graft loss until month 48.

H_{113}: There is an influence of treatment on the time to graft loss until month 48.

The second hypotheses system will be tested on an α_2-level of 1%. Therefore the following hypotheses system will be tested:

H_{02}: There is no influence of treatment on the proteomics pattern.

H_{12}: There is an influence of treatment on the proteomics pattern.

IN CASE OF EMERGENCY

In case of emergency one of the following persons must be contacted immediately. In the case of a serious adverse event (SAE) or pregnancy the CRO/Monitor must be contacted within one working day by fax as indicated below:

CRO/Monitor Dr. Andree Beckerling
Pierrel Research Europe GmbH
Address: Z. Katharina 6
45703 Essen
Phone: +49-(0)201-8990-0
Fax: +49-(0)201-8990-201

Medical Manager Roche Grenzach Prof. Stephan Korom
Address: Roche Pharma AG
D-79630 Grenzach-Wyhlen
Phone: +49 (0)7624 143668
Fax: +49 (0)7624 143143

Medical Manager Roche Grenzach Matthias Wahl
Address: Roche Pharma AG
D-79630 Grenzach-Wyhlen
Phone: +49 (0)7624 142142
Fax: +49 (0)7624 143143

If required drug safety should be informed.

Drug safety specialist Dr. Dorothea Thurn
Address: Roche Pharma AG
D-79630 Grenzach-Wyhlen
Phone: +49 (0)7624 143707
Fax: +49 (0)7624 143183

RESPONSIBILITIES

**Sponsor**	Roche Pharma AG
Contact Person:	Matthias Wahl
Address:	Emil-Barell Str. 1<br>D-79639 Grenzach-Wyhlen<br>Germany
Phone:	+49 (0)7624 142142
Fax:	+49 (0)7624 143143
E-mail:	Matthias.wahl@roche.com

**Coordinating Investigator**	
**(Leiter der klinischen Prüfung)**	Prof. Dr. med. Volker Kliem
Address:	Nephrologisches Zentrum Niedersachsen<br>Am Vogelsang 105<br>D-34346 Hann. Münden<br>Germany
Phone:	+49 (0)5541 996-328
Fax:	+49 (0) 5541 996-391
E-mail:	v.kliem@nzn.de

**CRO**	Pierrel Research Europe GmbH
Contact Person:	Dr. Andree Beckerling
Address:	Z. Katharina 6<br>D-45703 Essen<br>Germany
Phone:	+49-(0)201-8990-0
Fax:	+49-(0)201-8990-101
E-mail:	office@pierrel-research.com

**Data Management**	clinIT AG
Contact Person:	Dr. Max Horneck
Address:	Hornusstrasse 16
	D-79108 Freiburg
	Germany
Phone:	+49 (0) 761 50318 200
E-mail:	max.horneck@clinit.net

Central Technical Facilities:

**Laboratory for Proteomics Analysis:**	Mosaiques Diagnostics and Therapeutics AG
Contact Person:	Joachim Conrads
Address:	Mellendorferstr. 7-9
	D-30625 Hannover
	Germany
Phone:	+49 (0) 511 5547 4425
E-mail:	conrads@mosaiques-diagnostics.com

**Laboratory for CMV-Virus Detection:**	Labor Limbach
Contact Person:	Dr. Jan Bartel
Address:	Im Breitspiel 15
	D-69126 Heidelberg
	Germany
Phone:	+49 (0) 6221 3432137
E-mail:	jbartel@labor-limbach.de

FLOW CHART

	Study Phase															F-U Phase	Unscheduled Visit
	Base-line	Weekly				3-weekly								3-monthly		(twice per year)	during CMV therapy
**Visit**	**1**	**2**	**3**	**4**	**5**	**6**	**7**	**8**	**9**	**10**	**11**	**12**	**13**	**14**	**15**	**16-27**	**n.a.**
**Day[1]**	**-**	**7**	**14**	**21**	**28**	**42**	**63**	**84**	**100**	**126**	**147**	**168**	**196**	**280**	**364**		
**Week[1]**	**-**	**1**	**2**	**3**	**4**	**6**	**9**	**12**	**-**	**18**	**21**	**24**	**28**	**40**	**52**		
**Month[1]**	**-**				**1**		**2**		**3**		**4**	**5**	**6**	**9**	**12**	**18-84**	
**Allowed deviation**	**n.a.**	**± 3 d**	**± 3 d**	**± 5 d**	**± 5 d**	**± 1 wk**	**± 1 wk**	**± 1 wk**	**± 1 wk**	**± 1 wk**	**± 1 wk**	**± 1 wk**	**± 2 wk**	**± 4 wks**	**± 4 wks**	**± 8 wks**	
**Routine or Study related visit**	**R**	**R**	**R**	**R**	**R**	**S**	**S**	**R**	**S**	**S**	**S**	**S**	**R**	**R**	**R**	**S**	**n.a.**
Informed consent (S[2])	X																
Central randomization (S[2])	X																
Patient demographics, medical history (R[2])	X																
Graft assessment (R[2])	X	X	X	X	X	X	X	X	X	X	X	X	X	X	X	X	X
Assessments of CMV Disease (S[2])	X	X	X	X	X	X	X	X	X	X	X	X	X	X	X	X	X
Inclusion- / Exclusion criteria (R[2])	X																
Clinical examination (R[2])	X																
Vital signs (R[2]) / Actual body weight[3] (R[2])	X	X	X	X	X	X	X	X	X	X	X	X	X	X	X	X	X
Adverse events (R[2])		X	X	X	X	X	X	X	X	X	X	X	X	X	X	X[6]	X
Concomitant medication (R[2])	X	X	X	X	X	X	X	X	X	X	X	X	X	X	X	X	X

	Study Phase															F-U Phase	Unscheduled Visit
	Base-line	Weekly				3-weekly								3-monthly		(twice per year)	during CMV therapy
**Visit**	**1**	**2**	**3**	**4**	**5**	**6**	**7**	**8**	**9**	**10**	**11**	**12**	**13**	**14**	**15**	**16-27**	**n.a.**
**Day[1]**	**-**	**7**	**14**	**21**	**28**	**42**	**63**	**84**	**100**	**126**	**147**	**168**	**196**	**280**	**364**		
Days of hospitalization: Tx Unit / ICU / Reha (R[2])		X	X	X	X	X	X	X	X	X	X	X	X	X	X		
Pregnancy test (R[2])	X																
Safety Laboratory incl. Hematology (differential counts) (S[2]) and serum chemistry (R[2])	X	X	X	X	X	X	X	X	X	X	X	X	X	X	X		X
Serum creatinine[3] / Creatinine clearance (calculated) (R[2])	X	X	X	X	X	X	X	X	X	X	X	X	X	X	X	X	X
Urine sample for proteomics (S[2])	X	X	X	X	X	X	X	X	X	X	X	X	X	X	X	X[10]	
Urinary Status (S[2])	X	X	X	X	X	X	X	X	X	X	X	X	X	X	X	X	X
Fasting glucose/OGTT[8] (S[2])													X		X		
Proteinuria (S[2])					X				X				X		X		
HbA1c (S[2])					X				X				X		X		
Cobas® Amplicor® CMV MONITOR (quantitative PCR) (S[2])	X	X	X	X	X	X	X	X	X	X	X	X	X	X	X	X	X
Start of prophylaxis[4] (S[2])	X																
Dose adjustment based upon creatinine clearance[5] (S[2])		X	X	X	X	X	X	X									X

	Study Phase															F-U Phase	Unscheduled Visit
	Base-line	Weekly				3-weekly								3-monthly		(twice per year)	during CMV therapy
**Visit**	**1**	**2**	**3**	**4**	**5**	**6**	**7**	**8**	**9**	**10**	**11**	**12**	**13**	**14**	**15**	**16-27**	**n.a.**
**Day[1]**	**-**	**7**	**14**	**21**	**28**	**42**	**63**	**84**	**100**	**126**	**147**	**168**	**196**	**280**	**364**		
Dispense of study drug[5] (S[2])	X	X	X	X	X	X	X	X	X[7]	X[7]	X[7]	X[7]	X[7]	X[7]			X
Retrieval of study drug[5] (S[2])		X	X	X	X	X	X	X	X	X[7]	X[7]	X[7]	X[7]	X[7]	X[7]		X
Compliance check[5] (S[2])		X	X	X	X	X	X	X	X	X[7]	X[7]	X[7]	X[7]	X[7]	X[7]		X
End of prophylaxis[4] (S[2])									X								
Drug monitoring immuno-suppressive drugs[9] (R[2])	X	X	X	X	X	X	X	X	X	X	X	X	X	X	X	X	

[1] All assessments and visits will be based upon the day of transplantation, which is defined as day 0.

[2] R: data from routine measures, S: data from study related measures

[3] necessary for calculation of renal function with Cockroft-Gault formula

[4] Group A only

[5] Group A or during CMV therapy

[6] if study drug related

[7] in case of CMV therapy

[8] except for patients with preexisting diabetes mellitus type 1 or 2

[9] Trough level assessments of Calcineurin Inhibitors (CNI) are mandatory at each visit

[10] Only until months 24, after month 24 no further Proteomics sample will be taken.

GLOSSARY OF ABBREVIATIONS

AIDS	Acquired Immune Deficiency Syndrome
AE	Adverse Event
ALT	Alanine Aminotransferase
ANC	Absolute Neutrophile Count
AST	Aspartate Aminotransferase
ATG	Anti-Thymocyte Globuline
BAL	Bronchoalveolar lavage
CMV	Cytomegalovirus
CNI	Calcineurin inhibitor
CrCl	Creatinine Clearance
CRF	Case Report Form
CRO	Clinical research organization
CsA	Cyclosporine A
CSF	Cerebal Spinal Fluid
CTL	Cytotoxic T cell
D+	Donor CMV seropositive
D-	Donor CMV seronegative
DGF	Delayed graft function
DNA	Desoxyribonucleic Acid
ddI	Didanosine
ECG/EKG	Electrocardiogram
EDTA	Ethylenediamine tetraacetic acid
G-CSF	Granulocyte Colony Stimulating Factor
GM-CSF	Granulocyte Macrophage Colony Stimulating Factor
GFR	Glomerular Filtration Rate
HBV	Hepatitis B virus
HCV	Hepatitis C virus
HIV	Human immunodeficiency virus
HLA	Human leukocyte antigen
ICH	International Conference on Harmonization of Technical

GLOSSARY OF ABBREVIATIONS

	Requirements for Registration of Pharmaceuticals for Human Use
ICU	Intensive care unit
IE-1	Immediate early protein 1
IEC	Independent Ethics Committee
IgG	Immunoglobulin G
IgM	Immunoglobulin M
ITT	Intent to Treat
IRB	Institutional Review Board
IV	Intravenous
kg	kilogram
mg	milligram
MMF	Mycophenolat mofetil
n.a.	Not applicable
OGTT	Oral glucose tolerance test
OI	Opportunistic Infection
PBMC	Peripheral mononuclear cells
PCR	Polymerase chain reaction
PK	Pharmacokinetic
PP	Per Protocol
PTDM	Post-transplant Diabetes Mellitus
R^2	Routine measures / visits
R+	Recipient CMV seropositive
R-	Recipient CMV seronegative
RNA	Ribonucleic Acid
S^2	Study related measures / visits
SAE	Serious Adverse Event
SBRP	Secondary Basic Research Project
SOT	Solid Organ Transplant
SR	Sample Repository

GLOSSARY OF ABBREVIATIONS

μL	Microliter
ULN	Upper Limit of Normal
WBC	White Blood Cell

TABLE OF CONTENTS

PART I – STUDY DESIGN AND CONDUCT 21

1 BACKGROUND AND RATIONALE 21
1.1 Background 21
1.1.1 Study Drug 21
1.1.2 Disease 21
1.2 Rationale 23
1.2.1 Rationale for the Study, Relevance of the Study and Study Design 23
1.2.2 Rationale for Dosage Selection 23

2 OBJECTIVES OF THE STUDY 24
2.1 Primary Objective 24
2.2 Secondary Objectives 24

3 STUDY DESIGN 25
3.1 Overview of Study Design and Dosing Regimen 25
3.2 Number of Subjects/Assignment to Treatment (Groups/Sequences) 26
3.3 Centers 26
3.4 Study Duration 26
3.5 Secondary Basic Research Projects (SBRPs) 27

4 STUDY POPULATION 27
4.1 Target Population 27
4.2 Inclusion Criteria 27
4.3 Exclusion Criteria 28
4.4 Concomitant Medication and Treatment 28

5 SCHEDULE OF ASSESSMENTS AND PROCEDURES 29
5.1 Screening Examination and Eligibility Screening Form 34
5.2 Study Assessments 34
5.2.1 Clinical Assessments 34
5.2.2 Laboratory Assessments 38
5.2.3 Pharmacokinetic Assessments 41
5.2.4 Pharmacodynamic Assessments 41
5.2.5 Quality of Life Assessments (QoL) 41
5.2.6 Pharmacoeconomic Assessments 41
5.2.7 Schedule of Assessments 41
5.3 Planned Treatment of the Patient after Study End 44

6 INVESTIGATIONAL PRODUCT 44
6.1 Dose and Schedule of Test "Drug" and Comparator(s) 44
6.1.1 Patients with normal renal function 44

2001-06-MD2.2_01

6.1.2 Patients with impaired renal function 45
6.2 Preparation and Administration of Test "Drug" and Comparator(s) 46
6.2.1 Drug Names, Formulation and Storage 46
6.2.2 Packaging and Labeling 46
6.2.3 Route of Administration 47
6.3 Blinding and Randomization 47
6.4 Compliance 47
6.5 Background medication 48

7 SAFETY ISSUES 49
7.1 Adverse Events and Laboratory Abnormalities 49
7.1.1 Clinical Adverse Events 49
7.1.2 Laboratory Test Abnormalities 50
7.1.3 Adverse Events of Special Interest 50
7.2 Handling of Safety Parameters 50
7.2.1 Serious Adverse Events (Immediately Reportable to Roche) 50
7.2.2 Treatment and Follow-up of Adverse Events 50
7.2.3 Follow-up of Abnormal Laboratory Test Values 50
7.2.4 Pregnancy 51
7.3 Dose Modifications for Toxicity 51
7.4 Criteria for Discontinuation or Termination of the Study 52
7.4.1 Criteria for Discontinuation or Premature Withdrawal of the Patient 52
7.4.2 Criteria for Discontinuation or Termination of the Study 53
7.5 Warnings and Precautions 53

8 STATISTICAL CONSIDERATIONS 54
8.1 Definition of Population for Analysis 54
8.1.1 Intent-to-Treat Population 54
8.1.2 Per Protocol Population 54
8.1.3 Safety Population 54
8.2 Statistical and Analytical Methods 54
8.2.1 Statistical Model 54
8.2.2 Hypothesis Testing 56
8.2.3 Types of Analyses 57
8.2.4 Safety Data Analysis 58
8.2.5 Analysis of the follow up phase 59
8.2.6 Descriptive Statistics 60
8.3 Sample Size 60
8.4 Replacement Policy (Ensuring Adequate Numbers of Evaluable Subjects) 61
8.4.1 For Subjects 61
8.4.2 For Centers 61

9 DATA QUALITY ASSURANCE 62

10 STUDY COMMITTEES 62

11 REFERENCES 63

PART II - ETHICS AND GENERAL STUDY ADMINISTRATION 65

12 ETHICAL ASPECTS 65

12.1 Declaration of Helsinki/Good Clinical Practice 65

12.2 Patient Information and Informed Consent 65

12.3 Independent Ethics Committees and Regulatory Authorities 65

12.3.1 Approval of the Study by the Federal Regulatory Authority and Independent Ethics Committees 65

12.3.2 Notification of the Study 66

12.3.3 Report and Documentation Obligation 66

13 CONDITIONS FOR MODIFYING THE PROTOCOL 66

14 DISCONTINUATION OR EARLY TERMINATION OF THE STUDY 66

15 STUDY DOCUMENTATION, CRFS AND RECORD KEEPING 67

15.1 Investigator's Files/Retention of Documents 67

15.2 Source Documents and Background Data 67

15.3 Audits and Inspections 67

15.4 Case Report Forms 68

16 MONITORING THE STUDY 68

17 CONFIDENTIALITY OF TRIAL DOCUMENTS AND SUBJECT RECORDS 68

18 PUBLICATION OF DATA 69

19 APPENDICES 70

19.1 Appendix 1 Adverse Events Categories for Determining Relationship to Test Drug 70

19.2 Appendix 2 Definitions according to AMG and GCP-V, ICH Guidelines for Clinical Safety Data Management, Definitions and Standards for Expedited Reporting, Topic E2 72

19.3 Appendix 3 Grading of Severity of Adverse Events and Laboratory Abnormalities 74

19.4 Appendix 4 Assessment of acute allograft rejection 79

19.5 Appendix 5 Secondary Basic Research Project 2 (SBRP 2) 81

PART I – STUDY DESIGN AND CONDUCT

1 BACKGROUND AND RATIONALE

1.1 Background

1.1.1 Study Drug

Valganciclovir (Ro 107-9070) is a valyl ester prodrug of ganciclovir that is rapidly hydrolyzed to ganciclovir following ingestion. Conversion to ganciclovir occurs primarily during pre-systemic absorption, with only 1-2% of absorbed valganciclovir appearing as valganciclovir in the plasma, the remainder being found as ganciclovir. In solid organ transplant (SOT) recipients, exposure to ganciclovir from valganciclovir (1x900mg per day) averages 1.65 fold greater than that from oral ganciclovir (3x1g per day). [1]. The systemic exposure to the prodrug itself is low [1,2], and therefore, both the safety and efficacy profiles parallel those of ganciclovir. In AIDS patients, the initial and maintenance treatment of CMV retinitis with valganciclovir is as effective as the initial treatment with ganciclovir IV followed by maintenance with oral ganciclovir [3]. In SOT patients with D+/R- seroconstellation the rate of viremia until day 100 was significant lower with valganciclovir prophylaxis compared to prophylaxis with oral ganciclovir. Incidence of disease, graft and patient survival at one year was equivalent [4]. Valganciclovir with its high bioavailability is often used for pre-emptive treatment in SOT patients, whereas in the era before valganciclovir, CMV infections had to be treated with intravenous Ganciclovir and the respective complications of twice daily IV application [5-8]. The efficacy of valganciclovir and ganciclovir IV in the treatment of CMV disease is currently compared in a multicenter trial (MV 17973, EudraCT-No.: 2004-001946-34).

Valganciclovir is licensed for the induction and maintenance treatment of cytomegalovirus (CMV) retinitis in Acquired Immune Deficiency Syndrome (AIDS) patients and for the primary prophylaxis of CMV disease in high risk (donor CMV positive/ recipient CMV negative) SOT recipients.

The overall clinical experience with valganciclovir to date is summarized in the current version of the Investigator’s Brochure.

1.1.2 Disease

CMV belongs to the herpes family and is an opportunistic pathogene causing symptomatic infections in immunocompromized hosts. In healthy subjects a primary infection proceeds asymptomatic and the viral DNA integrates in the human genome and remains lifelong in human body cells (latent infection). About 60% of the adult population harbor CMV as latent infection, and can be identified by serologic detection of anti CMV antibodies. Thus in latent infected hosts frequently occurring CMV reactivation immediately is suppressed by CMV specific immunity.

CMV remains the most important serious infection complicating solid organ transplantation (SOT) [9]. Solid organ transplant recipients are at high risk of active CMV infection espe-

cially during the first 3 to 6 months after transplantation, which is the phase of high initial immunosuppression. CMV infection (viremia) can result in symptomatic CMV disease, which, in severe cases, can result in hospitalization, severe morbidity and in some cases, death. Additionally, active CMV infection is recognized as a risk factor for other poor short-term outcomes including acute allograft rejection [10]. Recipients, who never had contact with CMV and lack CMV immunity (R-) and receive an organ from a CMV positive donor (D+) are at highest risk developing CMV disease through reactivation of the virus in the transplanted organ. CMV positive recipients receiving an organ from a CMV positive or negative donor are at moderate risk (D+/R+ or D-/R+) and D-/R- patients show the lowest incidence of CMV disease because de novo infection during the phase of high immunosuppression is seldom (ca. 2 %).

Prevention of CMV disease is accomplished through primary prophylaxis with antiviral medication during the first 100 days after transplantation or through pre-emptive therapy, which means treatment of each viremia before symptoms occur involving frequent monitoring of viral load.

A registry analysis including 36000 patients showed a significant higher graft survival three years after transplantation with prophylaxis in D+/R- patients and a positive trend for R+ patients who received prophylaxis compared to patients without anti CMV prophylaxis [11]. Recommendations and guidelines strongly suggest antiviral prophylaxis in all D+/R- patients [12-14].

A recent metaanalysis including randomized trials evaluating antiviral prophylaxis showed that CMV prophylaxis with ganciclovir reduced mortality and morbidity significantly at study end points (3 to 12 months post TX) [15]. This was true for D+/R- serostatus at transplantation as well as for patients with R+ serostatus. Antiviral therapy against active infection was initiated in case of symptomatic disease (deferred therapy), however asymptomatic viremia remained untreated in all trials included in this analysis. Some authors state, that pre-emptive CMV therapy - initiated when the first viral load is detected in blood samples during close monitoring within the first year after transplantation - protects against CMV disease and functions as well as universal prophylaxis. Hence only a fraction of the patients are exposed to antiviral medication and their possible danger of adverse reactions [16,17].

Preemtive therapy is facilitated since a quantitative CMV PCR assay is available. Immunocompromised patients can be monitored with a quick and reliable test procedure for CMV viremia [18]. Cobas Amplicor CMV Monitor identifies CMV viremia with 95% sensitivity. Thus, patients at high risk for developing CMV disease can be treated pre-emptively [19]. On the other hand, universal prophylaxis not only decreases the incidence of CMV-infection and disease, but also shifts the peak level of occurrence from 30 days to 130 days after transplantation, a time, when immunosuppression in most cases is lowered from the initial induction phase and the course of CMV disease is milder [20]. The trend of developing cellular CMV immunity might be stimulated with lower immunosuppression as well [21].

In a prospective survey of 471 patients with a median follow up of 66 months Sagedal et al. [22] detected reduced long term survival of kidney transplant patients, who experienced symptomatic or even asymptomatic CMV-infection during the first 100 days after TX. This effect was significant, independent of the serostatus. The relative risk for over all mortality beyond 100 days was 2.5 in the case of CMV disease and even 2.9 in the case of asymptomatic CMV infection. Since asymptomatic CMV-infections were not treated in the investigated patient cohort, the authors conclude the following: “whether CMV prophylaxis or pre-emptive CMV therapy given routinely after kidney transplantation would improve patient survival remains to be shown”.

A German study group conducted a randomized trial comparing 90 days oral ganciclovir prophylaxis versus pre-emptive therapy with IV ganciclovir. Patients receiving prophylaxis showed a lower rate of infection, disease and hospitalization during the first year after transplantation independent of their seroconstellation (unpublished data). Improved outcome in terms of renal function, graft survival or mortality could not be demonstrated. In urine samples gathered during this trial, alteration of a proteomic pattern was detected, which seems dependent from active CMV infection and remains stable for at least one to three years. These patterns indicate graft alterations, which might lead in turn to reduced renal function or half-life of the graft within further years [23].

1.2 Rationale

1.2.1 Rationale for the Study, Relevance of the Study and Study Design

To date CMV preventing strategies aim to minimize acute complications, i.e. symptomatic CMV disease. Several prospective randomized studies show, that in high risk patients (D+/R-) this is best achieved with primary prophylaxis. To a lesser extend this is also true for R+ patients. However in these studies CMV infections were treated with deferred therapy.

The recently analyzed trial of a German study group mentioned above, showed a significant higher rate of CMV disease even with pre-emptive therapy compared to primary prophylaxis. Subpopulation analysis gave a significant difference in the D+/R- group but only a trend in the R+ group. In all subpopulations the rate of active CMV infection during the first year was extensively diminished by primary prophylaxis. Within the last few years, evidence is growing, that not only CMV disease but also asymptomatic (subclinical) active CMV infection correlate in SOT patients with increased long term morbidity, graft loss [22], diabetes [24], atherosclerosis [25,26] and mortality [15, 22] . This study is designed to give answers to two highly relevant pending questions: whether in renal transplant recipients with a positive CMV serostatus, who are closely monitored and treated pre-emptively in case of active CMV infection, a higher rate of active CMV infection and disease occurs during the first year compared to recipients treated with primary prophylaxis, and whether this goes along with a higher rate of chronic graft alteration monitored with a specific urine proteomic pattern at month 12. An additional 6 year follow up period is planned to observe long term graft and patient survival.

1.2.2 Rationale for Dosage Selection

The dosage of valganciclovir for prophylaxis, 900 mg once daily adjusted in case of impaired renal function, corresponds to the SPC.

The dosage of valganciclovir for pre-emptive therapy, 900 mg twice daily adjusted in case of impaired renal function, corresponds to the dosage for initial therapy of CMV retinitis in AIDS patients mentioned in the SPC. It is also according to the dosage in the ongoing trial of Valcyte for therapy of CMV disease in SOT patients (MV 17973) and in different publications about that theme [5-8].

2 Objectives of the Study

2.1 Primary Objective

The primary objective of this trial is to compare in CMV positive renal allograft recipients the efficacy of valganciclovir (900 mg once daily) prophylaxis applied until Day 100 post transplant with no prophylaxis under the condition of pre-emptive therapy of active CMV infection.

The corresponding primary endpoints are:

- Proportion of patients with active CMV infection within 12 months (plasma PCR ≥ 400 copies/ml).
- Proportion of patients with CMV disease within 12 months including CMV syndrome and tissue invasive disease.
- Urine proteomic pattern at month 12 on a scale between –1 = no graft alteration and +1 = graft alteration.
- Time to graft loss.

CMV disease includes both CMV syndrome and tissue invasive CMV. Clinical and laboratory findings, which define CMV syndrome and CMV disease are described in 5.2.1.9. Therefore at every visit the CMV viral load will be controlled in a plasma sample with a standardized quantitative PCR method (Cobas® Amplicor® CMV monitor®). For the assessment of urine proteomic pattern at every visit a 2 ml urine probe will be sampled, stored at -20°C and transferred every 3 months to the central laboratory for analysis.

2.2 Secondary Objectives

The secondary objective of this trial is to investigate the influence of both CMV prevention concepts on the occurrence of direct and indirect effects of active CMV infections: CMV disease (direct); renal function, rejections, opportunistic infections, graft- and patient survival, diabetes (indirect effects). In addition the safety of both concepts will be investigated by evaluating side effects, leucopenia/neutropenia, and infections.

The following variables will be evaluated for the acute phase:

1.a Proportion of patients with CMV syndrome within 12 months

1.b Proportion of patients with CMV tissue invasive disease within 12 months

2. Time to occurrence of first viremia (plasma PCR ≥ 400 copies/ml)
3. Viral burden at viremia (AUC of plasma PCR)
4. Creatinine clearance at month 12 (estimated by Cockroft-Gault formula as well as by MDRD formula, see 6.1.2))
5. Proportion of patients with treated and biopsy proven acute rejection episodes within 12 months
6. Cost survey including medication, monitoring, hospitalization
7. Correlation of proteomics pattern with graft survival
8. Correlation of proteomics pattern with patient survival
9. Predictive value of proteomics pattern for graft survival

10. Predictive value of proteomics pattern for patient survival
11. Hematological parameters including incidence of leucopenia (WBC of <3,500/µL and <1,000/µL) and neutropenia (ANC <750/µL) within 12 months
12. Proportion of patients with opportunistic infections within 12 months (bacterial, fungal, non CMV viral)
13. Patient survival at month 12
14. Graft survival at month 12
15. Proportion of patients with post transplant diabetes mellitus according to fasting glucose level or OGTT at month 6 and month 12
16. Incidence of active CMV infection not responding to valganciclovir or IV ganciclovir treatment

The following variables will be analyzed for the follow up phase:

Long term follow up:

17. Proportion of patients with CMV disease from baseline to month 18 and every 6 months up to months 84
18. Proportion of patients with CMV viremia from baseline to month 18 and every 6 months up to months 84
19. Proportion of patients with CMV syndrome from baseline to month 18 and every 6 months up to months 84
20. Proportion of patients with CMV tissue invasive disease from baseline to month 18 and every 6 months up to months 84
21. Proportion of patients who exhibit a specific urine proteomic pattern at month 18 and every 6 months up to months 84
22. Patient survival at month 18 and every 6 months up to months 84
23. Graft survival at month 18 and every 6 months up to months 84

3 STUDY DESIGN

3.1 Overview of Study Design and Dosing Regimen

This is an open label randomized comparative study with two parallel groups.

Patients in group A receive oral primary CMV prophylaxis with 1x 900 mg valganciclovir per day adjusted to renal function starting within 14 days of transplantation until Day 100 post transplantation.

Patients in group B receive no oral primary prophylaxis, only pre-emptive therapy as prescribed:

Patients in both groups are closely monitored for active CMV infection in plasma samples by quantitative PCR with Cobas® Amplicor® CMV Monitor. When plasma PCR is ≥ 400 copies/ml, pre-emptive therapy is given for at least 14 days with 2x 900 mg valganciclovir per day until second negative PCR (below 400 copies/ml) followed by secondary prophylaxis for 28 days with 1x 900 mg valganciclovir. In case of CMV disease or no response to Valganci-

2001-06-MD2.2_01

clovir treatment after 14 days (not falling viral load) IV ganciclovir or additional appropriate therapy can be administered according to the local site's standard instead of valganciclovir. After the study phase patients will be treated according to the local site´s standard. During this time (follow up phase) application of Study Drug supplied by the sponsor is not permitted.

The study phase is 12 months after transplantation. The patients will be observed for an additional follow up period of 6 years. All assessments and visits will be based upon the day of transplantation, which is defined as day 0.

3.2 Number of Subjects/Assignment to Treatment (Groups/Sequences)

300 patients are to be enrolled in the study.

Patients who are eligible for entering the study will first be thoroughly informed about the aims and details of the study. Prior to Visit 1, each patient will receive the lowest available screening number in the respective center, which starts with the letter "S" and consists of the site number and a consecutively increasing number in this center. For example, the first patient screened in center 1 will receive the screening number S01-001. This number as well as date of informed consent, date of birth will be recorded in the paper Patient Screening Log which will be kept in the Investigator Site File.

If at Visit 1 all required criteria for randomizing a patient are fulfilled the patient will be randomly assigned (ratio 1:1) to one of the two treatment groups by a telephone randomization system generated by the CRO responsible for data management (Köhler GmbH, Freiburg). This system will allocate the patient to one of the treatment groups and assign a patient number to each individual patient. Analogous to the screening number, the patient number starts with the letter "P" and consists of the site number and a consecutively increasing number in this center. For example, the first patient in center 1 will receive the patient number P01-001.

The assigned patient number will be entered into each patient's Case Report Form as well as into the Patient Identification Log.

3.3 Centers

Approximately 25 centers from Germany and Austria will participate in the study, each recruiting at least 10 patients.

A list of all participating investigational sites including information regarding names of the principal investigators and contact details (address, phone, fax email) will be handled separately.

3.4 Study Duration

The study is planned to start in Q2 2006 with respect to first patient in (FPI) including a recruitment period of 30 months and study phase of 12 months. The study phase will last approximately until Q4 2009 (last patient 12 months). The additional follow up period of 6 years will end Q4 2015 (last patient out). The total study duration is 9.5 years.

3.5 Secondary Basic Research Projects (SBRPs)

One secondary basic research projects (SBRP 2) will be conducted in conjunction with the main protocol for study ML 19313. All patients enrolled for the main protocol ML19313 are eligible for enrolment in the SBRP. Participation in the SBRP is entirely voluntary. A detailed study protocol for the SBRP is given in Appendix 5.

4 STUDY POPULATION

4.1 Target Population

Eligible patients are adult recipients of a kidney graft during the first 14 days of their hospital stay after transplantation.

Under no circumstances are patients who enroll in this study and who have completed treatment as specified, permitted to be re-randomized to this study and enrolled for a second course of treatment.

4.2 Inclusion Criteria

To be eligible for this trial, patients must have the following documented:

1. Indication: Patient has received within the preceding 14 days a primary or secondary renal allograft from a living or cadaveric donor.
2. Patient is IgG seropositive for CMV and has received an allograft from a CMV IgG seropositive or seronegative donor.
3. Patient receives immunosuppression including a CNI (CsA or tacrolimus) and MMF (mycophenolate mofetil)
4. Age range: Patient is 18 years of age or older.
5. Patient is willing to give written informed consent, written consent for data protection (legal requirement in Germany: datenschutzrechtliche Einwilligung) and willingness to participate and to comply with the study.
6. Laboratory parameters: Patient has adequate hematological and renal function defined as:
   a) Leucocyte count >3,500 cells/µL
   b) Platelet count >100,000 cells/µL
   c) Hemoglobin >8.0 g/dL
   d) Estimated creatinine clearance (calculated by the Cockcroft-Gault formula, see Section 6.1.2) of >10 ml/min with evidence of improving renal function.
7. Patient agrees to utilize contraceptive methods throughout the study period and for 90 days following discontinuation of the Study Drug. Examples of adequate contraceptive methods are intra-uterine device, barrier method (condoms, diaphragm), also in conjunction with spermicidal jelly, total abstinence, oral, injectable, or implant hormonal contraceptives. Male patients must agree to use condoms throughout the study period and for 90 days following discontinuation of the Study Drug.
8. Females of childbearing potential will have a negative pregnancy test at screening.

9. Patient is able to tolerate oral medication within 14 days post transplantation. The day of completion of transplant surgery is defined as Day 0 post transplantation.

4.3 Exclusion Criteria

Patients with any of the following will not be eligible for participation:

1. Patient has active CMV infection (CMV PCR ≥ 400 copies/ml)
2. Patients who had received an investigational new drug within the last 30 days
3. Patient is simultaneously participating in another clinical trial
4. Patient has received anti-CMV therapy within the past 30 days. (Acyclovir, valacyclovir, or famciclovir may be used for up to 10 days, at the dose specified in the package insert, for treatment of acute herpes simplex or herpes zoster.)
5. Patients who have participated in this study before
6. Women lactating, pregnant or of childbearing potential not using a reliable contraceptive method. Examples of adequate contraceptive methods are intra-uterine device, barrier method (condoms, diaphragm), also in conjunction with spermicidal jelly, total abstinence, oral, injectable, or implant hormonal contraceptives
7. Patients who are underage or patients who are incapable to understand the aim, importance and consequences of the study and to give legal informed consent (according to § 40 Abs. 4 and § 41 Abs. 2 und Abs. 3 AMG)
8. Patients with a history of a psychiatric illness or condition such as to interfere with the patient's ability to understand the requirements of the study
9. Patients who possibly are dependent on the sponsor or investigator
10. Patient has severe, uncontrolled diarrhea (multiple watery stools) or evidence of malabsorption
11. Patient has exhibited in the past an allergic or other significant adverse reaction to acyclovir, valacyclovir, ganciclovir, or valganciclovir
12. Patient has liver function test results greater than 3 times the upper limit of normal (ULN)
13. Patient is positive for HIV, Hepatitis B or Hepatitis C
14. Patients with malignancies or history of malignancy except non metastatic basal or squamous cell carcinoma of the skin that has been treated successfully
15. Patient has experienced acute steroid resistant rejection prior to randomization
16. Patient has a current severe illness or any other condition(s) (e.g. psychiatric disorder) which would make the subject, in the opinion of the investigator, unsuitable for the study
17. Patient is unlikely to be available for follow-up for the full duration of the study (12 months)
18. Patient received prohibited medication as defined in section 4.4 post transplantation.

4.4 Concomitant Medication and Treatment

All concomitant medications must be reported in the case report form.

2001-06-MD2.2_01

The use of the following concomitant medications during the clinical trial is prohibited:

- Prophylactic oral or IV acyclovir*
- Valacyclovir*
- Famciclovir*
- Ganciclovir **
- Valgancilovir (commercial supply)
- Valganciclovir for other causes than CMV primary prophylaxis during the first 100 days in arm A and (pre-emptive) CMV therapy including secondary prophylaxis in arm A and B.
- Cidofovir
- CMV hyperimmune globulin***
- Foscarnet***
- Lobucavir
- Probenecid
- Investigational drugs

* Acyclovir, valacyclovir, or famciclovir may be used for up to 10 days, at the dose specified in the package insert, for treatment of acute herpes simplex or herpes zoster.

** IV ganciclovir is permitted for therapy of CMV disease. Secondary prophylaxis must be done with valganciclovir study medication.

*** CMV hyperimmune globulin and Foscarnet are permitted in case of unsufficient response to therapy.

The following concomitant medications should be used with extreme caution:

- Agents that interfere with kidney function
- Imipenem-cilastatin
- The concomitant use of ganciclovir and imipenem-cilastatin has been associated with seizures, and this medication should only be used if the potential benefits outweigh the potential risks.

Drug Interactions:

- Didanosine (ddI)

In PK studies, the concomitant use of ganciclovir and didanosine (ddI) has been associated with increases in ddI concentrations of greater than 100%. Accordingly, the possibility of increased ddI toxicity should be considered in patients receiving both medications.

5 SCHEDULE OF ASSESSMENTS AND PROCEDURES

A summary of the protocol required assessments and procedures is provided in Table 1 (Schedule of Assessments and Procedures) on the following pages. Dates of visits should be scheduled in advance (according to the date transplantation) and adhered to wherever possi-

ble. In the case of late or missed visits, the original schedule should be used for subsequent visits and visits should not be re-scheduled. All assessments and visits will be based upon the day of transplantation, which is defined as day 0.

Unless otherwise indicated procedures performed not specifically for the study prior to inclusion are accepted for the study, provided that they were done between 1 month pre-transplant and 14 days post-transplant. Study Drug must commence within 14 days post-transplant.

If visit 1 is performed within day 7 and 14 post-transplant, visits 2 and 3 will be skipped, respectively.

Table 1: Schedule of Assessment and Procedures

	**Study Phase**															**F-U Phase**	**Unscheduled Visit**
	**Base-line**	**Weekly**				**3-weekly**								**3-monthly**		**(twice per year)**	**during CMV therapy**
**Visit**	**1**	**2**	**3**	**4**	**5**	**6**	**7**	**8**	**9**	**10**	**11**	**12**	**13**	**14**	**15**	**16-27**	**n.a.**
**Day[1]**	**-**	**7**	**14**	**21**	**28**	**42**	**63**	**84**	**100**	**126**	**147**	**168**	**196**	**280**	**364**		
**Week[1]**	**-**	**1**	**2**	**3**	**4**	**6**	**9**	**12**	**-**	**18**	**21**	**24**	**28**	**40**	**52**		
**Month[1]**	**-**				**1**		**2**		**3**		**4**	**5**	**6**	**9**	**12**	**18-84**	
**Allowed deviation**	**n.a.**	**± 3 d**	**± 3 d**	**± 5 d**	**± 5 d**	**± 1 wk**	**± 1 wk**	**± 1 wk**	**± 1 wk**	**± 1 wk**	**± 1 wk**	**± 1 wk**	**± 2 wk**	**± 4 wks**	**± 4 wks**	**± 8 wks**	
**Routine or Study related visit**	**R**	**R**	**R**	**R**	**R**	**S**	**S**	**R**	**S**	**S**	**S**	**S**	**R**	**R**	**R**	**S**	**n.a.**
Informed consent (S[2])	X																
Central randomization (S[2])	X																
Patient demographics, medical history (R[2])	X																
Graft assessment (R[2])	X	X	X	X	X	X	X	X	X	X	X	X	X	X	X	X	X
Assessments of CMV Disease (S[2])	X	X	X	X	X	X	X	X	X	X	X	X	X	X	X	X	X
Inclusion- / Exclusion criteria (R[2])	X																
Clinical examination (R[2])	X																
Vital signs (R[2]) / Actual body weight[3] (R[2])	X	X	X	X	X	X	X	X	X	X	X	X	X	X	X	X	X
Adverse events (R[2])		X	X	X	X	X	X	X	X	X	X	X	X	X	X	X[6]	X
Concomitant medication (R[2])	X	X	X	X	X	X	X	X	X	X	X	X	X	X	X	X	X

	Study Phase															F-U Phase	Unscheduled Visit
	Base-line	Weekly				3-weekly								3-monthly		(twice per year)	during CMV therapy
**Visit**	**1**	**2**	**3**	**4**	**5**	**6**	**7**	**8**	**9**	**10**	**11**	**12**	**13**	**14**	**15**	**16-27**	**n.a.**
**Day[1]**	**-**	**7**	**14**	**21**	**28**	**42**	**63**	**84**	**100**	**126**	**147**	**168**	**196**	**280**	**364**		
Days of hospitalization: Tx Unit / ICU / Reha (R[2])		X	X	X	X	X	X	X	X	X	X	X	X	X	X		
Pregnancy test (R[2])	X																
Safety Laboratory incl. Hematology (differential counts) (S[2]) and serum chemistry (R[2])	X	X	X	X	X	X	X	X	X	X	X	X	X	X	X		X
Serum creatinine[3] / Creatinine clearance (calculated) (R[2])	X	X	X	X	X	X	X	X	X	X	X	X	X	X	X	X	X
Urine sample for proteomics (S[2])	X	X	X	X	X	X	X	X	X	X	X	X	X	X	X	X[10]	
Urinary Status (S[2])	X	X	X	X	X	X	X	X	X	X	X	X	X	X	X	X	X
Fastig glucose/OGTT[8] (S[2])													X		X		
Proteinuria (S[2])					X				X				X		X		
HbA1c (S[2])					X				X				X		X		
Cobas® Amplicor® CMV MONITOR (quantitative PCR) (S[2])	X	X	X	X	X	X	X	X	X	X	X	X	X	X	X	X	X
Start of prophylaxis[4] (S[2])	X																
Dose adjustment based upon creatinine clearance[5] (S[2])		X	X	X	X	X	X	X									X
Dispense of study drug[5]	X	X	X	X	X	X	X	X	X[7]	X[7]	X[7]	X[7]	X[7]	X[7]			X

	Study Phase															F-U Phase	Unscheduled Visit
	Base-line	Weekly				3-weekly								3-monthly		(twice per year)	during CMV therapy
**Visit**	**1**	**2**	**3**	**4**	**5**	**6**	**7**	**8**	**9**	**10**	**11**	**12**	**13**	**14**	**15**	**16-27**	**n.a.**
**Day[1]**	**-**	**7**	**14**	**21**	**28**	**42**	**63**	**84**	**100**	**126**	**147**	**168**	**196**	**280**	**364**		
(S[2])																	
Retrieval of study drug[5] (S[2])		X	X	X	X	X	X	X	X	X[7]	X[7]	X[7]	X[7]	X[7]	X[7]		X
Compliance check[5] (S[2])		X	X	X	X	X	X	X	X	X[7]	X[7]	X[7]	X[7]	X[7]	X[7]		X
End of prophylaxis[4] (S[2])									X								
Drug monitoring immuno-suppressive drugs[9] (R[2])	X	X	X	X	X	X	X	X	X	X	X	X	X	X	X	X	

[1] All assessments and visits will be based upon the day of transplantation, which is defined as day 0.

[2] R: data from routine measures, S: data from study related measures

[3] necessary for calculation of renal function with Cockroft-Gault formula

[4] Group A only

[5] Group A or during CMV therapy

[6] if study drug related

[7] in case of CMV therapy

[8] except for patients with preexisting diabetes mellitus type 1 or 2

[9] Trough level assessments of Calcineurin Inhibitors (CNI) are mandatory at each visit

[10] Only until month 24. After 24 months no further sample for Proteomics will be taken.

5.1 Screening Examination and Eligibility Screening Form

An eligibility screening form (ESF) documenting the subject's fulfillment of the entry criteria for all patients considered for the study and subsequently included or excluded, is to be completed by the investigator and forwarded to the CRO. All patients undergoing screening activities (documented by completion of an ESF for each patient) must be listed in the Patient Screening Log. For details see section 3.2.

5.2 Study Assessments

5.2.1 Clinical Assessments

"Invasive" measures are measures penetrating the body by injuring the integrity of the body. Measures are defined as non-invasive measures in case of intramuscular, intravenous or subcutaneous injections, infusions, application of cannulas or dermal tissue biopsies.

No invasive measures are part of the study procedures.

5.2.1.1 Informed consent (S^2)

As described in detail in section 12.2 written informed consent must be obtained before any study specific procedures are performed. The completed informed consent form must be available on the day of the baseline visit (Visit 1) at latest.

5.2.1.2 Patient demographics, medical history (R^2)

At the baseline visit (Visit 1) the demographic details such as date of birth, sex, height and ethnic group as well as a full interim medical history will be documented including:

- Previous transplants or major surgical operations
- Primary reason for transplant
- Rejection episodes
- Urinary tract infections
- Active medical conditions and treatments
- History of cancer
- History of blood disorders
- Opportunistic infections (bacterial, viral and fungal)
- Method of contraception
- Patient, donor and transplant information (CMV serostatus (IgG required), age, Human Leukocyte Antigen (HLA) typing information, panel reactive antibodies, cold ischemia time)
- Record whether randomization was delayed post-transplant due to DGF, inability to tolerate oral medication or other reason

5.2.1.3 Graft assessment (R^2)

The graft status has to be assessed at every visit.

Acute Allograft Rejection

In the absence of medical contraindications, all rejection episodes should be biopsy confirmed. Renal biopsies will be performed as medically indicated. Biopsies should be assessed histologically using the updated Banff criteria 1997 (see section 19.5). The results will be reported to the Sponsor on the appropriate Case Report Form (Rejection Episode).

All treatments for acute allograft rejection will be captured on the Rejection page (type [steroids or antibodies] and specification) as well as Concomitant Medication page (daily dose and duration) of the CRF.

Protocol Biopsy

According to center specific requirements it is permitted to perform protocol biopsies. Since the performance of a protocol biopsy is to be expected in this patient population it will be documented on the CRF but not reported as Serious Adverse Event (SAE). Protocol Biopsies should be assessed histologically using the updated Banff 1997. The results will be reported on the appropriate Case Report Form (Rejection Episode). Results or biopsy specific side effects meeting the SAE criteria have to be reported to Roche within one working day of knowledge.

Graft Loss

Graft loss is defined as the institution of chronic dialysis (at least 6 consecutive weeks), transplant nephrectomy, or retransplantation. Reasons for graft loss must be recorded on the CRF.

5.2.1.4 Inclusion- / Exclusion criteria (R^2)

The inclusion- and exclusion criteria as described in detail in section 4.2 and 4.3 will be assessed at the baseline visit (Visit 1).

5.2.1.5 Clinical examination (R^2)

A general physical examination at the baseline visit (Visit 1) should include the examinations of the following body systems:

- *Eyesight*
- *Ear, nose and throat diseases*
- *Cardiac diseases*
- *Cardiovascular diseases*
- *Pulmonary disease*
- *Blood formation and coagulation*
- *Bone marrow*
- *Endocrine system*
- *Pancreas*
- *Gastrointestinal tract*
- *Hepatobiliary system*
- *Urogenital tract*
- *Renal function*
- *Skin*
- *Musculoskeletal system*
- *Peripheral lymph nodes*
- *Neurology*
- *Malignancies*
- *Pain*
- *Opportunistic bacterial infections*
- *Opportunistic viral infections*
- *Opportunistic fungal infections*
- *Other infections*
- *Other*

All items included into the physical examination form have to be examined in such detail as relevant to the current study.

5.2.1.6 Vital signs (R^2) / Actual body weight[1] (R^2)

At every visit vital signs (temperature, blood pressure, heart rate) and weight will be assessed and documented in the CRF.

5.2.1.7 Adverse events (R^2)

Adverse events (serious and non-serious) encountered during treatment and up to month 12 will be reported on the adverse event page of the CRF. Graft rejection, opportunistic infections, active CMV infection, CMV syndrome and CMV tissue invasive disease have to be reported on the corresponding pages of the CRF and not on the adverse event page. The patient should be questioned about any ongoing or any new AEs/OIs since last assessment at every visit. In addition, an AE that occurs after these times if considered related to Study Drug, should be reported.

For more detailed definition of adverse events and description of the documentation and reporting procedures see section 7.1.

5.2.1.8 Concomitant medication and diseases (R^2)

Any change in concomitant medication during the course of the study as well as onset of new medication in accordance with the exclusion criteria has to be recorded by the investigator in the relevant section of the CRF. Note: any newly occurred disease or conditions and/or a deterioration of a current disease or condition have to be documented as adverse events in section AE of the CRF.

Especially immunosuppressant and further concomitant medication (except those associated with surgery e.g. anesthetics and analgesics) since randomization of the patient has to be recorded e.g. treatment of acute rejections or opportunistic infections.

Opportunistic Infection

The following infections, which occur during the course of the study, will be recorded on the appropriate Case Report Form: Aspergillus, Candida, Pneumocystis, Cryptococcus, Listeria, Herpes zoster, and Herpes simplex. Treatments for OIs will be recorded on the CRF.

Recurrence of original disease in the transplanted kidney

It has to be assessed and documented by the investigator, if recurrence of the original disease occurred.

5.2.1.9 Assessments of active CMV Infection and CMV Disease

The following clinical and laboratory findings should be verified before an active CMV infection or CMV disease is documented.

Active CMV infection:

In case of active CMV infection (Viremia according plasma PCR ≥ 400 copies/ml - measured with the standardized quantitative PCR Roche Cobas Amplicor CMV Monitor), Valcyte prophylaxis has to be stopped and Valcyte therapy has to be started according to 6.1.1.2. The patient should be visited and documented once weekly until CMV-Monitor is below 400 copies/ml at two successive visits. After end of therapy and secondary prophylaxis, restart Valcyte prophylaxis in group A until Day 100.

CMV syndrome:

Viremia according plasma PCR ≥ 400 copies/ml (measured with the standardized quantitative PCR Roche Cobas Amplicor CMV Monitor) and at least one of the following signs:

- Fever of ≥ 38°C
- Severe malaise (toxicity grading ≥ 3 (see section 19.3)
- Leucopenia on 2 successive measurements separated by at least 24 hours (defined as (1) a white blood cell (WBC) count of <3,500/µL or (2) a WBC decrease of >20% if the WBC count prior to development of viremia is <4,000/µL.)
- Atypical lymphocytosis of ≥5%
- Thrombocytopenia (defined as (1) a platelet count of <100,000/µL or (2) a decrease of >20% if the platelet count prior to development of viremia is <115,000/µL.)
- Elevation of hepatic transaminases (alanine aminotransferase (ALT) or aspartate aminotransferase (AST) to at least 2X ULN).

Viremia will be measured in a plasma sample with the standardized quantitative PCR Roche Cobas Amplicor CMV Monitor at every visit. All signs will be documented at every visit.

CMV tissue invasive disease:

Viremia according plasma PCR ≥ 400 copies/ml and at least one of the following signs:

Clinical evidence of localized CMV infection (CMV inclusion cells or *in situ* detection of CMV antigen or DNA by immunostain or hybridization, respectively) in a biopsy or other appropriate specimen (e.g., Bronchoalveolar Lavage (BAL), Cerebal Spinal Fluid (CSF)) *and / or* relevant symptoms or signs of organ dysfunction.

All other possible causes of the observed symptoms (e.g. acute rejection if the organ is the allograft) must be excluded as a possible cause for the patient's clinical findings.

The diagnosis of organ-specific tissue-invasive CMV disease also requires the following documentation:

- CMV hepatitis: At least one liver function test value (AST, ALT, alkaline phosphatase, or bilirubin) will be at least 2X ULN. CMV hepatitis may be confirmed by liver biopsy. The biopsy will be characterized by the following:
  Presence of cells with positive immunostaining, immunofluorescence, *in situ* hybridization for CMV, CMV inclusions, or a positive viral culture in conjunction with histologic findings compatible with CMV hepatitis.

- CMV esophagitis, gastroenteritis, or colitis: Clinically, at least *one* of the following will be present: Signs and symptoms of upper gastrointestinal tract infection, including nausea, vomiting, anorexia, dysphagia, odynophagia, abdominal pain, or cramping. In addition, at least one of the following will be present: Signs and symptoms of colitis, including persistent diarrhea or abdominal pain.
  The diagnosis can be confirmed by biopsy. The biopsy will be characterized by the presence of cells with positive immunostaining, immunofluorescence, *in situ* hybridization for CMV, or CMV inclusions.
- CMV pneumonia. Clinically, at least *two* of the following are required: Cough, Dyspnea, Compatible infiltrates on chest-x-ray, Hypoxemia (PaO_2 <80 mmHg or O_2 saturation <90% on room air).
  The diagnosis can be confirmed by BAL or biopsy. The BAL or biopsy will be characterized by the presence of cells with positive immunostaining, immunofluorescence, *in situ* hybridization for CMV, CMV inclusions, or positive viral culture. In a case where the only evidence of CMV infection is by viral culture, the presence of other significant pathogens must be excluded.
- CMV retinitis. The diagnosis will be based upon a dilated fundus examination by an ophthalmologist experienced in the diagnosis of CMV retinitis.
- Other tissue invasive CMV disease where the presence of CMV will be confirmed by examination of a biologic specimen (e.g., tissue biopsy, CSF) and the patient exhibits signs or symptoms of relevant organ dysfunction most likely resulting from CMV. The demonstration of CMV antigen or CMV DNA in the CSF of a patient with the clinical picture of CMV encephalitis or polyradiculitis will suffice to confirm such a diagnosis.

The clinical assessment of a patient with suspected CMV syndrome will include, as a minimum, appropriate blood cultures to rule out bacterial, fungal and other OIs as a cause of the patient's symptoms. Other diagnostic laboratory procedures (e.g., urine cultures, chest x-ray) will be performed as warranted by the patient's clinical status and standard medical practices at the Study Center.

If the patient's symptoms meet the protocol definition of either (1) CMV syndrome or (2) tissue invasive CMV, the patient will be treated for CMV disease according 6.1.1.2.

Evaluation of Patient by Local Physician

Investigators should encourage patients to return to the study center if the patient suspects they may have CMV disease. If it is necessary for a local physician to evaluate patients for suspected CMV disease, the investigator must ensure that the local physician makes all the appropriate assessments and collects the appropriate samples as described above.

5.2.2 Laboratory Assessments

5.2.2.1 Pregnancy test (R^2)

A pregnancy test (ß-HCG in serum) will be performed for all female patients of childbearing potential at the baseline visit and at any time a secondary amenorrhea of > 1 week duration occurs during the first 52 weeks post transplant according to the routine procedures of each investigational center. The result of the test must be available prior to the administration of study drug.

5.2.2.2 Safety Laboratory incl. Hematology (differential counts) (S^2) and serum chemistry (R^2)

A safety laboratory will be conducted according to the routine procedures of each investigational center. The valid normal ranges including the methods for all parameters will be handed over to the CRO prior to the start of the study.

All laboratory values outside the normal range will be evaluated and commented upon by the investigator. The following parameters will be determined:

Hematology
Hematology tests including hemoglobin, hematocrit, WBC count with differential, absolute neutrophil count (ANC), and platelet count will be obtained at the following scheduled visits: During screening activities (post-transplant and within 48 hours of randomization), at baseline prior to first dosing on the day treatment is started and on every following visit.

Serum chemistry tests
Total bilirubin, liver function tests (AST, ALT, gGT, alkaline phosphatase), total protein, albumin, uric acid, glucose, electrolytes (Na, K), calcium, phosphate, serum creatinine and BUN/urea) CRP, will be obtained at baseline, prior to first dosing on the day that treatment is started and every following visit.

In centers in which serum Cystatin C is routinely measured for assessment of renal function, this parameter should be documented in the CRF.

5.2.2.3 Serum creatinine[1] (R^2) / Creatinine clearance (calculated)

The patient's estimated creatinine clearance, based on the Cockcroft-Gault formula (see Section 6.1.2) will be calculated at each protocol-defined clinical assessment that includes the measurement of serum creatinine. In addition, all patients with an estimated creatinine clearance of less than 60 ml/min will have a serum creatinine measurement at least twice weekly, and more frequently if warranted by changes in renal function, during their initial period of hospitalization following transplantation. Based on the estimated creatinine clearance, the doses of Study Drugs will be modified in accordance with Table 2, Section 6.1.

5.2.2.4 Urine sample for proteomics (S^2)

2 ml urine sample for proteomics will be collected and stored at -20°C at each visit. The last sample taken for proteomics will be at month 24. After that visit no further samples for proteomics will be taken.

5.2.2.5 Fasting Glucose - OGTT (S^2)

Except for patients with preexisting diabetes mellitus (type 1 or 2) fasting glucose will be measured at visits 13 and 15.

If the glucose concentration is above 109 mg/dl (6,0 mmol/l) the result must be confirmed with a second measurement and diabetes mellitus diagnosed. If the glucose concentration is between 90 and 109 mg/dl (5,0 – 6,0 mg/dl) an OGTT has to be performed according to the following procedure.

After a 12 h fasting period give the patient a drink with 75 g oligosaccharides. During the following 120 min the patient must sit.

Measure glucose level in capillary blood taken at before intake, 60 min and 120 min after intake of oligosaccharide drink.

If the 120 min glucose level is above 200 mg/dl (11,1 mmol/l), diabetes mellitus is diagnosed. If the 120 min glucose level is between 140 and 200 mg /dl (7,8 – 11,1 mmol/l) impaired glucose tolerance is diagnosed.

5.2.2.6 Proteinuria (S^2)

Proteinuria in 24 hrs collection urine will be obtained at visits 5, 9, 13, and 15.

5.2.2.7 HbA1c (S^2)

HbA1c in serum will be obtained at visits 5, 9, 13, and 15.

5.2.2.8 CMV viral load: Cobas® Amplicor® CMV MONITOR (quantitative PCR) (S2)

The Cobas® Amplicor® CMV Monitor® will be used by each Study Center to test for CMV viremia as part of the clinical assessment at scheduled (Visit 1 to Visit 27) as well as unscheduled Visits. Each study center will send 10 ml EDTA blood to the central laboratory immediately at the day of the visit. Assay results will be returned by Fax within 36 hours to allow effective management of patients with suspected CMV disease.

5.2.2.9 CMV IgM (R^2)

CMV IgM will not be part of the routine laboratory assessment. The Cobas® Amplicor® CMV Monitor® will be used to test for CMV viremia as part of the clinical assessment at every scheduled (Visit 1 to Visit 27) and unscheduled Visit.

5.2.2.10 Drug monitoring immunosuppressive drugs (R^2)

Trough levels of Calcineurin Inhibitors (CNI) will be part of the routine laboratory assessment at every visit.

5.2.2.11 Urinary Status (R^2)

The following measures will be taken at each Visit to assess the urinary status (possible outcomes in brackets):

- *Nitrit (negativ / positiv)*
- *Protein (negativ / + / ++ / +++)*
- *Hämoglobin, Blood (negativ / positiv)*
- *Bacteriuria (negativ / + / ++ / +++)*
- *Leucocytes (clin. significant / normal)*
- *Erythrocytes (clin. significant / normal)*

5.2.3 Pharmacokinetic Assessments

Not applicable

5.2.4 Pharmacodynamic Assessments

Not applicable

5.2.5 Quality of Life Assessments (QoL)

Not applicable

5.2.6 Pharmacoeconomic Assessments

Days of hospitalization: Tx Unit / ICU / Reha (R^2)

All hospital and ICU admissions/discharges have to be documented from visit 2 until visit 15.

5.2.7 Schedule of Assessments

5.2.7.1 Baseline visit (Visit 1)

Patients providing written informed consent will undergo further evaluations as listed in Table 1 (and summarized below) to determine if they meet all entry criteria.

Unless otherwise indicated procedures performed not specifically for the study prior to inclusion are accepted for the study, provided that they were done between 1 month pre-transplant and 14 days post-transplant.

However, pregnancy test, serum chemistries, hematology, CMV PCR and calculated creatinine clearance assessments must occur post-transplant and within 48 hours prior to randomization. The following examinations/procedures or determinations have to be performed and documented for each patient at baseline visit:

- Informed consent (S^2)
- Central randomization (S^2)
- Patient demographics, medical history (R^2)
- Graft assessment (R^2)
- Assessments of CMV Disease (S^2)
- Inclusion- / Exclusion criteria (R^2)
- Clinical examination (R^2)
- Vital signs (R*) / Actual body weight (R^2)
- Concomitant medication (R^2)
- Pregnancy test (R^2)
- Safety Laboratory incl. Hematology (differential counts) (S^2) and serum chemistry (R^2)
- Serum creatinine / Creatinine clearance (calculated) (R^2)

- Urine sample for proteomics (S^2)
- Blood sample for Cobas® Amplicor® CMV MONITOR (quantitative PCR) (S^2)
- Start of prophylaxis (S^2)
- Dispense of study drug (S^2)
- Drug monitoring immunosuppressive drugs (R^2)
- Urinary Status

5.2.7.2 Weeks 2 to 52 Post Transplant

During weeks 2 to 52 post transplant full assessments of the patient at the transplant center are required as follows:

- Visits 2-5: weekly up to week 4 (±3 days for V2 and V3 and ±5 days for V4 and V5)
- Visits 6-13: three weekly (±1 week except V13, V13 ±2 weeks) up to week 28
- Visits 14-15: three monthly (±4 weeks) up to week 52

The required assessments and procedures, which are summarized below (see also Table 1 and the separate samples procedures manual) have to be performed and documented for each patient:

- Graft assessment (R^2)
- Assessments of CMV Disease (S^2)
- Vital signs (R^2) / Actual body weight (R^2)
- Adverse events (R^2)
- Concomitant medication (R^2)
- Days of hospitalization: Tx Unit / ICU / Reha (R^2)
- Safety Laboratory incl. Hematology (differential counts) (S^2) and serum chemistry (R^2)
- Serum creatinine / Creatinine clearance (calculated) (R^2)
- Urine sample for proteomics (S^2)
- Blood sample for Cobas® Amplicor® CMV MONITOR (quantitative PCR) (S^2)
- Drug monitoring immunosuppressive drugs (R^2)
- Urinary Status

In addition, at visits 2 to 9 the following assessments and procedures have to be performed:

- Dispense of study drug (S^2, not at visit 9)
- Dose adjustment based upon creatinine clearance (S^2, not at visit 9)
- Retrieval of study drug (S^2)
- Compliance check (S^2 as described in section 6.4)

- End of prophylaxis (S^2, only visit 9)

In addition, at visits 5, 9, 13 and 15 the following assessments and procedures have to be performed:

- Fasting glucose, OGTT (S^2, visits 13 and 15 only); not applicable for patients with preexisting diabetes mellitus (type 1 or 2)
- HbA1c (S^2)
- Proteinuria

5.2.7.3 Year 2 to 7 Post Transplant

During year 2 to 7 post transplant full assessments of the patient at the transplant center are required as follows in six monthly intervals (Visits 16-27):

- Graft assessment (R^2)
- Assessments of CMV Disease (S^2)
- Vital signs (R^2) / Actual body weight (R^2)
- Adverse events (R^2, only if study drug related)
- Concomitant medication (R^2)
- Serum creatinine / Creatinine clearance (calculated) (R^2)
- Urine sample for proteomics (S^2) until Visit 24
- Blood sample for Cobas® Amplicor® CMV MONITOR (quantitative PCR) (S^2)
- Drug monitoring immunosuppressive drugs (R^2)
- Urinary Status

5.2.7.4 Unscheduled visits

In case of CMV therapy according to section 6.1.1.2 unscheduled visits must be documented weekly. Following assessments are required:

- Graft assessment (R^2)
- Assessments of CMV Disease (S^2)
- Vital signs (R^2) / Actual body weight (R^2)
- Adverse events (R^2)
- Concomitant medication (R^2)
- Safety Laboratory incl. Hematology (differential counts) (S^2) and serum chemistry (R^2)
- Serum creatinine / Creatinine clearance (calculated) (R^2)
- Blood sample for Cobas® Amplicor® CMV MONITOR (quantitative PCR) (S^2)
- Dispense of study drug (S^2)

- Dose adjustment based upon creatinine clearance (S^2)
- Retrieval of study drug (S^2)
- Compliance check (S^2 as described in section 6.4)
- Urinary Status

If a weekly visit during CMV therapy occurs on a scheduled visit day, please document as scheduled visit.

5.3 Planned Treatment of the Patient after Study End

After completion of the study the patient will be transferred to the routine therapy. The investigator takes responsibility for decision about the subsequent treatment.

6 Investigational Product

(a) Investigational Medicinal Product (IMP)

According to § 3 (3) GCP-V an investigational medicinal product is a pharmaceutical form of an active substance or placebo being tested or used as a reference in a clinical trial, including products already with a marketing authorization but used or assembled (formulated or packaged) in a way different from the authorized form, or when used for an unauthorized indication, or when used to gain further information about the authorized form.

- The IMP in this study is valganciclovir film-coated tablets containing 450 mg valganciclovir
- There is no comparator applied

(b) Background Medication see 6.5.

6.1 Dose and Schedule of Test "Drug" and Comparator(s)

6.1.1 Patients with normal renal function

6.1.1.1 Primary prophylaxis

Primary prophylaxis treatment with Study Drug, 900 mg valganciclovir once daily, will be started in group A as soon as the patient is able to take oral tablets following surgery, but in no case later than post transplant Day 14, and will continue until Day 100 post transplant. It is anticipated that in most instances the first doses of Study Drug will be taken with the morning meal. Patients, however, may receive their first doses of Study Drug with the mid-day or evening meal. Regardless of when the first dose of Study Drug was taken, all subsequent doses should be taken with the morning meal.

The comparator in group B is no treatment.

6.1.1.2 Treatment of active CMV infection

In case of active CMV infection (viral load ≥ 400 copies/ml serum) within the evaluation period of 12 months patients in group A and group B receive treatment with study drug, valganciclovir 2 x 2 tablets (1800mg) per day adjusted to renal function. In case of CMV disease or if the patient is unable to take oral medication IV ganciclovir 2 x 5mg/kg body weight per day will be permitted. The treatment will be continued for a minimum of 14 days until at least two subsequent PCR measurements below 400 copies/ml. Subsequently follows a secondary prophylaxis with valganciclovir 1 x 2 tablets (900mg) per day adjusted to renal function for 28 days. In case of CMV disease or no response to Valganciclovir treatment after 14 days (not falling viral load) IV ganciclovir or additional appropriate therapy can be administered according to the local site's standard instead of valganciclovir. After the study phase of 12 months patients will be treated according to the local site's standard. During this time (follow up phase) application of Study Drug supplied by the sponsor is not permitted.

6.1.2 Patients with impaired renal function

The dose of valganciclovir will be adjusted as needed for reduced renal function based on calculated creatinine clearance. Actual renal creatinine clearance will be estimated on every visit from serum creatinine, patient age and weight using the Cockcroft-Gault formula as follows:

$$\text{creatinine clearance (males)} = \frac{(140 - \text{age}\,[\text{years}]) \times (\text{body weight}\,[\text{kg}])}{(\text{serum creatinine}\,[\text{mg/dL}]) \times (72)}$$

or

$$\text{creatinine clearance (males)} = \frac{(140 - \text{age}\,[\text{years}]) \times (\text{body weight}\,[\text{kg}])}{(0.011 \times \text{serum creatinine}\,[\text{micromol/L}]) \times (72)}$$

$$\text{creatinine clearance (females)} = \text{male value} \times 0.85$$

Depending on the calculated creatinine clearance, dose will be modified in accordance with Table 2:

Table 2 Dose Adjustments for Creatinine Clearance

Creatinine clearance (mL/min)	Prophylaxis Valganciclovir (450 mg Tablets)	Pre-emptive therapy Valganciclovir (450 mg Tablets)
**≥ 60**	2 tablets (900 mg) QD	2 tablets (900 mg) twice daily
**40 - < 60**	1 tablet (450 mg) QD	1 tablet (450 mg) twice daily
**25 - < 40**	1 tablet (450 mg) every other day	1 tablet (450 mg) QD
**10 - < 25**	1 tablet (450 mg) twice weekly	1 tablet (450 mg) every other day
**< 10 or patient on dialysis)**	**Interrupt treatment with Study Drug.**	**Interrupt treatment with Study Drug.**

Whenever dosage has to be changed, a dosing card with the actual dosage marked should be handed over to the patient.

Treatment with Study Drug will be interrupted if a patient requires dialysis or if the patient's creatinine clearance decreases to <10 mL/min. If the patient's renal function has not increased to ≥10 mL/min within 7 days, a Roche Medical Science Representative will be contacted to discuss the patient.

If treatment for CMV syndrome is initiated in the absence of a viremia test result and the subsequent viremia result is negative, the patient is considered not to have CMV syndrome. In this situation, once a negative viremia test has been reported, treatment should be discontinued and the patient should resume study medication and assessments as stipulated in the protocol.

6.2 Preparation and Administration of Test "Drug" and Comparator(s)

6.2.1 Drug Names, Formulation and Storage

Study drug is valganciclovir 450 mg film-coated tablets (Ro 107-9070) and will be supplied by the Sponsor.

The Study Drug should be stored in safe place, accessible exclusively to individually authorized personnel. The Study Drug is to be kept away from direct sunlight in a dry, cool, place. Storage conditions for valganciclovir are below 35°C.

6.2.2 Packaging and Labeling

The investigational medicinal product valganciclovir will be labeled according to § 5 GCP-V and internal requirements. Valganciclovir will be supplied as 450-mg tablets in plastic bottles each containing 60 tablets within a white paper box.

Each bottle will bear a label containing the following statements in German language:

- ML19313, Nächste Haltbarkeitsprfg:________
- 60 Tabletten Valganciclovir 450 mg
- 107-9070/J06, Zum Einnehmen. Ch.-B.:________
- Gemäss Anweisung Ihres Arztes einnehmen.
- Vorsicht – Um eine Überdosierung zu vermeiden, müssen die Dosierempfehlungen strikt eingehalten werden.
- Arzneimittel für Kinder unzugänglich aufbewahren.
- Zur Klinischen Prüfung bestimmt. Abgabedatum:________
- Leere Packungen und nicht verwendete Arzneimittel zurückgeben.
- Sponsor: Roche Pharma AG, D-79630 Grenzach-Wyhlen
- CRO: IFE Europe GmbH, 45307 Essen

Each box will bear a label containing the following statements in German language:

- ML19313, Nächste Haltbarkeitsprfg:________
- 60 Tabletten Valganciclovir 450 mg
- 107-9070/J06, Zum Einnehmen. Ch.-B.:________
- Gemäss Anweisung Ihres Arztes einnehmen.

- Vorsicht – Um eine Überdosierung zu vermeiden, müssen die Dosierempfehlungen strikt eingehalten werden.
- Arzneimittel für Kinder unzugänglich aufbewahren.
- Zur Klinischen Prüfung bestimmt. Abgabedatum:________
- Leere Packungen und nicht verwendete Arzneimittel zurückgeben.
- Sponsor: Roche Pharma AG, D-79630 Grenzach-Wyhlen
- CRO: IFE Europe GmbH, 45307 Essen

6.2.3 Route of Administration

Valganciclovir is administered orally, and should be taken with food.

6.3 Blinding and Randomization

As this is an open trial, blinding is not applicable.

If all required criteria for randomizing a patient are fulfilled the patient will be randomly assigned (ratio 1:1) to one of the two treatment groups by a telephone randomization system generated by the CRO responsible for data management (Köhler GmbH, Freiburg).

At the time of assignment of the Patient Study Number, a qualified member of the medical staff at the Study Center will telephone the Central Randomization Center to obtain the patient's treatment assignment. The Randomization Center will be available 24 hours per day 7 days per week and can be reached by telephone. This system will allocate the patient to one of the treatment groups and assign a patient number to each individual patient. The assigned patient number will be entered into the each patient's Case Report Form as well as into the Patient Identification Log. For details please refer to section 3.2.

Randomization will be stratified by center and by induction immunosuppression with polyclonal antibodies, such as ATG, ALG or OKT® 3.

The randomization list will not be available at the study center, to the Roche monitors, project statisticians or to the project team at Roche.

A study patient must not be randomized to treatment until the Study Center is certain that all enrollment criteria have been met, including the likely ability to take oral medication.

6.4 Compliance

Start of prophylaxis (S^2) / Dispense of study drug (S^2)

Patients in group A will receive one bottle with 60 tablets of Study Drug for primary prophylaxis on an approximately monthly basis. In case of dose adjustment the time period elongates accordingly. At week 16 the patient returns the tablets residual after finishing the prophylaxis at Day 100 post transplant.

Patients in group B do not receive any primary prophylaxis.

In case of therapy of an active CMV infection (PCR ≥ 400 copies/ml) patients in both groups receive 1 bottle with 60 tablets of study drug. After 15 days at the latest, the next bottle has to be handed over to the patient until PCR is below 400 copies/ml. During the subsequent secondary prophylaxis the patient receives study drug for 28 days. In case of dose adjustment the time periods for re-supply of study drug elongate accordingly.

A preprinted drug dispensing log is integrated in the CRF. The amount of medication dispensed to or returned by the patient at each visit has to be documented in the CRF according to the requirements.

Based on potentially necessary dose adjustment the investigator needs to check at each visit during the treatment period, if the patients have an adequate amount of medication for the period to the next visit.

Retrieval of study drug (S^2)

All medication supplies (empty containers, as well as partly used and unused medication) must be available for inspection at every monitoring visit. All unused medication, partly-used and empty packages have to be shown by the patients to the investigator who counts the remaining tablets and re-dispense them to the patient. At the end of the treatment the investigator retains the unused medication as well as the empty bottles and returns them to the sponsor.

Safety precautions for handling of study drug

In animal studies, ganciclovir was found to be mutagenic, teratogenic, aspermatogenic and carcinogenic. Valganciclovir should therefore be considered a potential teratogen and carcinogen in humans with the potential to cause birth defects and cancers. Caution should be observed in handling valganciclovir. Tablets should not be broken or crushed, and direct contact of broken or crushed tablets, and of powder or reconstituted oral solution, with skin or mucous membranes should be avoided. If such contact occurs, wash thoroughly with soap and water, and rinse eyes thoroughly with plain water. Care should be taken not to inhale valganciclovir powder.

Compliance check (S^2)

The patient will be regarded as non-compliant, if he/she took less than 80% of the stipulated study medication they were assigned to take for primary prophylaxis.

6.5 Background medication

Immunosuppression will consist of a calcineurinhibitor (CsA or tacrolimus), mycophenolate mofetil and steroids. Initial immunosuppressive therapy with monoclonal antibodies (daclizumab or basiliximab) or polyclonal antibodies (ATG, ALG, thymoglobuline) is permitted. Induction therapy with polyclonal antibodies is parameter of stratification. Steroid dosage will be tapered according to center practice and discontinued as decided by the investigator. The CNI dosage will be tapered according to center practice but not discontinued. Mycophenolate mofetil will be applied with 2g per day, dose adaptation is permitted based on systematic drug monitoring or in case of incompatibility reaction. During active CMV infection, CNI or MMF may be reduced but not discontinued. After resolving of incompatibility reactions or active CMV infection, appropriate dosages of CNI and MMF should be restored.

Background medication will not be supplied or reimbursed by Roche.

7 SAFETY ISSUES

7.1 Adverse Events and Laboratory Abnormalities

It is the responsibility of the investigator(s) to report all adverse events in the case report form. Any serious adverse event (SAE) must be reported to Roche within one working day.

7.1.1 Clinical Adverse Events

An adverse event is any untoward medical occurrence in a patient or clinical investigation subject administered a pharmaceutical product and which does not necessarily have to have a causal relationship with this treatment. An adverse event can therefore be any unfavorable and unintended sign (including an abnormal laboratory finding, for example), symptom, or disease temporally associated with the use of a medicinal product, whether or not considered related to the medicinal product. Pre-existing conditions which worsen during a study are to be reported as Adverse Events. They can become Serious Adverse Events if they fulfill one of the seriousness criteria described in section 19.2.

All clinical adverse events (serious and non-serious) encountered during treatment and up to month 14 will be reported on the adverse event page of the CRF. Graft rejection, opportunistic infections, active CMV infection, CMV syndrome and CMV tissue invasive disease have to be reported on the corresponding pages of the CRF and not on the adverse event page. In addition, an adverse event that occurs after this time if considered related to Study Drug, should be reported. Intensity of adverse events will be graded on a four-point scale (mild, moderate, severe, life-threatening) and reported in detail as indicated on the CRF (see W.H.O. Handbook for Reporting Results of Cancer Treatment).

Mild:	Discomfort noticed but no disruption of normal daily activity.
Moderate:	Discomfort sufficient to reduce or affect daily activity.
Severe:	Inability to work or perform normal daily activity
Life-threatening:	Represents an immediate threat to life

Relationship of the adverse event to the treatment should also be assessed. Description of scales can be found in section 19.1.

The term “severe” is a measure of intensity; thus, a severe adverse event is not necessarily a serious adverse event in accordance with ICH guidelines (see Serious Adverse Events, Section 7.2.1). For example, nausea of several hours' duration may be rated as severe, but may not be clinically serious.

For all adverse events, the following information must be assessed and recorded on the Adverse Event page of the Case Report Form: Intensity, Investigator’s assessment of relationship to test medication, action taken, and outcome to date.

7.1.1.1 Expected adverse events

Adverse events resulting as a consequence of the transplantation itself (i.e. graft dysfunction, graft rejection, opportunistic infection, active CMV infection, CMV syndrome, CMV tissue invasive disease) will not be reported as SAEs in this study since such events are to be ex-

pected in this patient population and are documented on the corresponding pages (new rejection episode, new opportunistic infection, cmv infection and disease) in the CRF. The data will be analyzed with the results analysis at the end of the study. Additionally, with the exception of immunosuppressive medications, concomitant medications associated with the transplant surgery (e.g. anesthetics and analgesics) will not be recorded.

7.1.2 Laboratory Test Abnormalities

Laboratory test results will be recorded on the laboratory results pages of the Case Report Form, or appear on electronically produced laboratory reports submitted directly from the central laboratory, if applicable. Laboratory test value abnormalities as such should not be reported on the AE page of the CRF as adverse events, unless there is an associated clinical condition for which the patient is given treatment or concomitant treatment altered, it is considered to be a serious adverse event, if the patient is permanently discontinued from study drug because of the abnormal test value.

7.1.3 Adverse Events of Special Interest

Not applicable.

7.2 Handling of Safety Parameters

7.2.1 Serious Adverse Events (Immediately Reportable to Roche)

Any clinical adverse event or abnormal laboratory test value that is serious occurring during the course of the study up to month 14, irrespective of the treatment received by the patient, must be reported to Roche within one working day of knowledge (expedited reporting). In addition, an adverse event that occurs after this time if considered related to test "drug", should be reported.

Routine invasive procedures resulting as a consequence of the transplantation itself (i.e. Protocol Biopsy of the renal allograft, removal of Double-J-Catheter) will not to be reported as Serious Adverse Events since such procedures are part of standard care in this patient population. Adverse effects or obtained results of the described routine procedures meeting the SAE criteria have to be reported to Roche within one working day of knowledge.

The definition and reporting requirements according to German Drug Law, GCP-V and ICH Guideline for Clinical Safety Data Management, Definitions and Standards for Expedited Reporting, Topic E2 will be adhered (for details refer to section 19.2).

7.2.2 Treatment and Follow-up of Adverse Events

Adverse events, especially those for which the relationship to test "drug" is not "unrelated", should be followed up until they have returned to baseline status or stabilized. If a clear explanation is established it should be recorded on the CRF.

7.2.3 Follow-up of Abnormal Laboratory Test Values

In the event of unexplained abnormal laboratory test values, the tests should be repeated immediately and followed up until they have returned to the normal range and/or an adequate

explanation of the abnormality is found. If a clear explanation is established it should be recorded on the CRF.

7.2.4 Pregnancy

A female subject must be instructed to stop taking the study drug and immediately inform the investigator if she becomes pregnant during the study. Pregnancies occurring up to 90 days after the completion of the test "drug" must also be reported to the investigator. The investigator must report all pregnancies within one working day to the sponsor. The investigator should counsel the subject, discuss the risks of continuing with the pregnancy and the possible effects on the fetus. Monitoring of the patient should continue until conclusion of the pregnancy.

If consent is given, pregnancy occurring in the partner of a subject participating in the study should also be reported to the investigator and the sponsor. The partner should be counseled and followed as described above.

7.3 Dose Modifications for Toxicity

Hematology Abnormalities

The following are recommendations for dose modifications for patients with clinically significant changes in hematology parameters.

Neutropenia.

*For patients **not** receiving other concomitant medications that may cause neutropenia:*

If the absolute neutrophil count (ANC) falls below 750 cells/µL, treatment with granulocyte colony stimulating factor (G-CSF) or granulocyte macrophage colony stimulating factor (GM-CSF) may be given. Study Drug therapy need not be interrupted if G-CSF or GM-CSF therapy is initiated and the ANC is >750 cells/µL within 24 hours of initiation of G-CSF or GM-CSF therapy.

If the ANC falls below 750 cells/µL and therapy with G-CSF or GM-CSF is either not instituted or does not raise the ANC within 24 hours, the Study Drug should be withheld until the ANC recovers to ≥750 cells/µL. Patients may then resume therapy with Study Drug according to standard dosing instructions.

With the exception of dose modifications due to impaired renal function, under no circumstances should the study drug be dose reduced. Study drug should be interrupted completely or administered to the patient as per standard dosing instructions.

For patients receiving other concomitant medications that may cause neutropenia:

If the ANC falls below 750 cells/µL, treatment with G-CSF or GM-CSF may be given. Study Drug need not be interrupted if the G-CSF or GM-CSF therapy is initiated and the ANC is >750 cells/µL within 24 hours. If the ANC is not >750 cells/µL within 24 hours, either the Study Drug or the concomitant medication suspected to be related to the neutropenia should be interrupted or reduced (concomitant medications only, study drug should be interrupted and **not** dose reduced) until the ANC has returned to >750 cells/µL.

Whether the study drug or concomitant medication is interrupted/reduced is subject to the investigators medical judgment. A risk assessment to ascertain the likely clinical consequences

following dose interruption/reduction of each drug should be performed. The drug likely to be of least potential clinical consequence should be interrupted or reduced.

Thrombocytopenia. If the platelet count falls below 25,000/µL, Study Drug should be interrupted until the count has returned to ≥ 25,000/µL, at which time treatment may be resumed.

Anemia. In cases of severe anemia, defined as a hemoglobin level of less than 8.0 g/dL, transfusion or erythropoietin therapy may be instituted in lieu of dose modification. Severe anemia may require interruption of Study Drug or, if applicable, the concomitant medication suspected to be related to the anemia.

AEs which May Be Related to Treatment with Study Drug

Patients experiencing other AEs of Grade 3 or 4 toxicity that are thought to be related to treatment with Study Drug should have therapy withheld until the toxicity returns to ≤ Grade 2 or the patient's baseline value (see section 19.3 for grading the severity of adverse events and laboratory abnormalities). If severe toxicity (i.e., Grade 3 or 4) thought to be related to treatment with Study Drug recurs, therapy should again be withheld as described above. Further therapy should be given only after re-evaluation of the risks/benefits of further treatment with Study Drug and should be discussed with the Roche Medical Science Representative.

Valacyclovir, a chemically related valinated nucleoside analog, has been associated with a thrombotic microangiopathy (hemolytic-uremic syndrome, thrombotic thrombocytopenic purpura) (TTP) in immunocompromised patients when administered at a dose of 8 grams. TTP and/or microangiopathy should be adequately ruled out when investigating thrombocytopenia. If signs of such a disorder should occur in a patient receiving valganciclovir, the drug should be discontinued and Roche Medical Science Representative contacted immediately.

AEs not Believed to be Related to Treatment with Study Drug

Dosing for patients with impaired renal function will be reduced in accordance with the dosing schedule provided in Section 6, Table 2.

7.4 Criteria for Discontinuation or Termination of the Study

7.4.1 Criteria for Discontinuation or Premature Withdrawal of the Patient

Subjects have the right to withdraw from the study at any time for any reason. The investigator also has the right to withdraw subjects from the study in the event of intercurrent illness, adverse events and treatment failure after a prescribed procedure, protocol violations, cure, administrative reasons or other reasons. An excessive rate of withdrawals can render the study uninterpretable; therefore, unnecessary withdrawal of subjects should be avoided. Should a subject decide to withdraw, all efforts will be made to complete and report the observations as thoroughly as possible.

The investigator should contact the subject to determine as completely as possible the reason for the withdrawal. A complete final evaluation at the time of the patient's withdrawal should be made with an explanation of why the subject is withdrawing from the study. If the reason for removal of a subject from the study is an adverse event or an abnormal laboratory test result, the principal specific event or test will be recorded on the Case Report Form.

In case of premature withdrawal from the study this must be documented on the Study Completion Form in the CRF. All assessments according to visit 15 (Follow up visit 27) have to be performed, if possible.

Compliance/Study Drug Tolerability

Regardless of the reason, if valganciclovir therapy is withheld for either >14 consecutive days or >21 days in any 28 day period, the patient should be terminated from the treatment phase of the study but complete all remaining scheduled assessments through post transplant week 52 in accordance with the protocol.

7.4.2 Criteria for Discontinuation or Termination of the Study

Criteria which could lead to discontinuation or termination of the study are described in section 14.

7.5 Warnings and Precautions

No evidence available at the time of the approval of this study protocol indicated that special warnings or precautions were appropriate, other than those noted in the Investigators' Brochure. Since kidney transplant patients often show varying renal function, creatinine clearance has to be calculated on every visit and dosage has to be adjusted to actual renal function to protect the patient from overdosage.

8 STATISTICAL CONSIDERATIONS

8.1 Definition of Population for Analysis

8.1.1 Intent-to-Treat Population

Intent-to-treat population (ITT) is defined to include all patients randomized (if applicable) who received at least one dose of study medication and where the primary variable was measured at least once under study medication.

8.1.2 Per Protocol Population

Per protocol population (PP) is defined to include all patients of the intent-to-treat population excluding those patients with major protocol violations.

8.1.3 Safety Population

The safety population is defined to include all patients who received at least one dose of the trial medication and a safety follow-up, whether withdrawn prematurely or not, will be included in the safety analysis.

8.2 Statistical and Analytical Methods

8.2.1 Statistical Model

8.2.1.1 Primary Variables

The following efficacy variables will be analyzed based on the ITT population.

- Proportion of patients with active CMV infection within 12 months (plasma PCR ≥ 400 copies/ml).
- Proportion of patients with CMV disease within 12 months including CMV syndrome and tissue invasive disease.
  Syndrome defined as viremia according plasma PCR ≥ 400 copies/ml and at least one of the following signs:
  - Fever of ≥38°C
  - new or increased malaise
  - leucopenia on 2 successive measurements separated by at least 24 hours (defined as
    - a WBC of <3,500/µL or
    - a WBC decrease of >20% if the WBC prior to development of viremia is <4,000/µL);
  - atypical lymphocytosis of ≥5%
  - thrombocytopenia defined as
    - a platelet count of <100,000/µL or
    - a decrease of >20% if the platelet count prior to development of viremia is <115,000/µL)
  - elevation of hepatic transaminases (ALT or AST) to at least 2xULN.

Tissue invasive disease defined as viremia according plasma PCR ≥ 400 copies/ml and evidence of localized CMV infection (CMV inclusion cells or in situ detection of CMV antigen or DNA by immunostain or hybridization, respectively) in a biopsy or other appropriate specimen (e.g., Bronchoalveolar Lavage (BAL), Cerebal Spinal Fluid (CSF)) and/or relevant symptoms or signs of organ dysfunction.

- Urine proteomic pattern at month 12 on a scale between –1 = no graft alteration and +1 = graft alteration.
- Time to graft loss.

For patients who discontinue from treatment prematurely the time to CMV viremia, the time to CMV disease and the time to graft loss will be classified as censored if the respective event was not observed prior to the premature discontinuation.

8.2.1.2 Secondary Variables

The following efficacy variables will be analyzed:

- Proportion of patients with CMV syndrome within 12 months
- Proportion of patients with CMV tissue invasive disease within 12 months
- Time to occurrence of first viremia (plasma PCR ≥ 400 copies/ml)
- Viral burden at viremia (AUC of plasma PCR)
- Creatinine clearance at month 12 (estimated by Cockroft-Gault formula as well as by MDRD formula)
- Proportion of patients with treated and biopsy proven acute rejection episodes within 12 months
- Cost survey including medication, monitoring, hospitalization
- Correlation of proteomics pattern with graft survival
- Correlation of proteomics pattern with patient survival
- Predictive value of proteomics pattern for graft survival
- Predictive value of proteomics pattern for patient survival

The following safety variables will be analyzed:

- Hematological parameters including incidence of leucopenia (WBC of < 3,500/µL and < 1,000/µL) and neutropenia (ANC < 750/µL) within 12 months
- Proportion of patients with opportunistic infections within 12 months (bacterial, fungal, non CMV viral)
- Patient survival at month 12
- Graft survival at month 12
- Proportion of patients with post transplant diabetes mellitus according to fasting glucose, OGTT at month 6 and month 12
- Incidence of active CMV infection not responding to valganciclovir or IV ganciclovir treatment

The following variables will be analyzed for the follow up phase:

- Proportion of patients with CMV disease from baseline to month 18 and every 6 months up to months 84
- Proportion of patients with CMV viremia from baseline to month 18 and every 6 months up to months 84
- Proportion of patients with CMV syndrome from baseline to month 18 and every 6 months up to months 84
- Proportion of patients with CMV tissue invasive disease from baseline to month 18 and every 6 months up to months 84
- Proportion of patients who exhibit a specific urine proteomic pattern at month 18 and every 6 months up to months 84
- Patient survival at month 18 and every 6 months up to months 84
- Graft survival at month 18 and every 6 months up to months 84

8.2.2 Hypothesis Testing

The following two hypotheses systems will be tested simultaneously based on a global type I error of α = 5% using a correction with type I errors of α_1= 4% and α_2= 1%:

The first hypotheses system will be carried out in a hierarchic test procedure with an α_1-level of 4%. Therefore the following three hypotheses systems will be tested hierarchically:

> H_{011}: There is no difference between the two treatment groups in the proportion of patients with active CMV infection within 12 months.
>
> H_{111}: There is a difference between the two treatment groups in the proportion of patients with active CMV infection within 12 months.

The time to CMV viremia within 12 months (plasma PCR ≥ 400 copies/ml) will be analyzed for each treatment group using the method of Kaplan-Meier. The proportions of patients with CMV viremia at 12 months are then estimated from the analysis of survival times as 1 – survival rate at 12 months. The standard deviations are determined for both estimations. Subsequently, the difference of the proportion of patients with CMV viremia at 12 months is calculated and the 95% confidence interval is determined by means of its standard error (square root of the sum of the single squared standard errors). If the value ‘0‘ lies outside of this interval the result is interpreted as statistically significant. This procedure corresponds to a two-sided test on an α-level of 5%.

In case of a non-significant result for the hypothesis H_{011}, the hierarchic test procedure terminates. In case of a significant result the hypothesis H_{012} can be tested.

> H_{012}: There is no difference between the two treatment groups in the proportion of patients with CMV disease within 12 months including CMV syndrome and tissue invasive disease.
>
> H_{112}: There is a difference between the two treatment groups in the proportion of patients with CMV disease within 12 months including CMV syndrome and tissue invasive disease.

The time to CMV disease within 12 months including CMV syndrome and tissue invasive disease will be analyzed for each treatment group using the method of Kaplan-Meier. The proportions of patients with CMV disease at 12 months are then estimated from the analysis of survival times as 1 – survival rate at 12 months. The standard deviations are determined for both estimations. Subsequently, the difference of the proportion of patients with CMV disease at 12 months is calculated and the 95% confidence interval is determined by means of its standard error (square root of the sum of the single squared standard errors) If the value '0' lies outside of this interval the result is interpreted as statistically significant. This procedure corresponds to a two-sided test on an α-level of 5%.

In case of a non-significant result for the hypothesis H_{012}, the hierarchic test procedure terminates. In case of a significant result the hypothesis H_{013} can be tested.

H_{013}: There is no influence of treatment on the time to graft loss.

H_{113}: There is an influence of treatment on the time to graft loss.

The time to graft loss will be analyzed using a proportional hazard model with CMV viremia, treatment and the interaction of treatment and CMV viremia as the influencing factors.

The second hypotheses system will be tested on an α_2-level of 1%. Therefore the following hypotheses system will be tested:

H_{02}: There is no influence of treatment on the proteomics pattern.

H_{12}: There is an influence of treatment on the proteomics pattern.

The proteomics pattern will be analyzed using an ANOVA model with CMV viremia, treatment and the interaction of treatment and CMV viremia as the influencing factors.

8.2.3 Types of Analyses

8.2.3.1 Efficacy Analysis

The following efficacy variables will be analyzed using descriptive statistics (i.e. mean, standard deviation, minimum, lower quartile, median, upper quartile and maximum for continuous data and frequencies and percentages for categorical data) and the described methods:

- The proportion of patients with CMV syndrome within 12 months will be compared between the two treatment groups using Fisher's exact test.
- The proportion of patients with CMV tissue invasive disease within 12 months will be compared between the two treatment groups using Fisher's exact test.
- The time to occurrence of first viremia (plasma PCR $\geq$ 400 copies/ml) will be compared between the two treatment groups using survival methods.
- The viral burden at viremia (time weighted AUC of plasma PCR) will be compared between the two treatment groups using descriptive methods only.

- The creatinine clearance at month 12 (estimated by Cockroft-Gault formula as well as by MDRD formula) will be compared between the two treatment groups using descriptive methods only.
- The proportion of patients with treated and biopsy proven acute rejection episodes within 12 months will be compared between the two treatment groups using descriptive methods only.
- The cost survey including medication, monitoring, hospitalization will be compared between the two treatment groups using descriptive methods only.
- For the estimation of the correlation of proteomics pattern with graft survival descriptive statistics of the proteomics pattern will be calculated for patients with and without graft survival separately.
- For the estimation of the correlation of proteomics pattern with patient survival descriptive statistics of the proteomics pattern will be calculated for patients who survived or did not survive separately.
- To analyze the predictive value of the proteomics pattern for graft survival the cut-off value which result in a 90% prediction of graft survival and the cut-off value which result in a 90% prediction of graft loss will be calculated.
- To analyze the predictive value of the proteomics pattern for patient survival the cut-off value which result in a 90% prediction of patient survival and the cut-off value which result in a 90% prediction of patient death will be calculated.

8.2.3.2 Exclusion of Data from Analysis

The exclusion of data from analysis will be decided on in a Data Review Meeting.

8.2.3.3 Interim Analysis

For the first hypotheses system the analyses of the primary variables are based on the data after 12 months for the first two steps of the hypothesis system. For the third step of the first hypothesis system the analysis will be based on the data after 48 months. For the second hypotheses system the analyses of the primary variable are based on the data after 12 months. These evaluations will be carried out as these data are available. No adjustment of the α-level is necessary as no further confirmatory analyses will be carried out.

8.2.4 Safety Data Analysis

The following safety variables will be analyzed:

- The hematological parameters including incidence of leucopenia (WBC of < 3,500/µL and < 1,000/µL) and neutropenia (ANC < 750/µL) within 12 months will be compared between the two treatment groups using descriptive methods only.
- The proportion of patients with opportunistic infections within 12 months (bacterial, fungal, non CMV viral) will be compared between the two treatment groups using descriptive methods only.

- The patient survival rate at month 12 will be compared between the two treatment groups using descriptive methods only.
- The graft survival rate at month 12 will be compared between the two treatment groups using descriptive methods only.
- The proportion of patients with post transplant diabetes mellitus according to fasting glucose, OGTT at month 6 and month 12 will be compared between the two treatment groups using descriptive methods only.
- The incidence of active CMV infection not responding to valganciclovir or IV ganciclovir treatment will be compared between the two treatment groups using descriptive methods only.

8.2.5 Analysis of the follow up phase

The following variables will be analyzed for the follow up phase:

- The proportion of patients with CMV disease from baseline to month 18 and every 6 months up to months 84 will be compared between the two treatment groups using descriptive methods only.
- The proportion of patients with CMV viremia from baseline to month 18 and every 6 months up to months 84 will be compared between the two treatment groups using descriptive methods only.
- The proportion of patients with CMV syndrome from baseline to month 18 and every 6 months up to months 84 will be compared between the two treatment groups using descriptive methods only.
- The proportion of patients with CMV tissue invasive disease from baseline to month 18 and every 6 months up to months 84 will be compared between the two treatment groups using descriptive methods only.
- The proportion of patients who exhibit a specific urine proteomic pattern at month 18 and every 6 months up to months 84 will be compared between the two treatment groups using descriptive methods only.
- The patient survival rate at month 18 and every 6 months up to months 84 will be compared between the two treatment groups using descriptive methods only.
- The graft survival rate at month 18 and every 6 months up to months 84 will be compared between the two treatment groups using descriptive methods only.

8.2.5.1 Pharmacoeconomic Analysis

Analysis of pharmacoeconomic data and production of a final pharmacoeconomic report will be handled separately from the final clinical report of this study. Information obtained from the collection of medical care utilization data in this study may be combined with other data such as cost data or other clinical parameters in the production of a final PE report.

8.2.6 Descriptive Statistics

By the mid of 2008 descriptive statistics of the study population will be done on a quarterly basis and subsequently reviewed by a study committee according to section 10 of this protocol.

8.3 Sample Size

The third step of the hierarchic test procedure has lowest power in the hierarchy. Therefore, the following sample size calculation is based on the third step of the hierarchic test procedure within the first hypotheses system assuming a graft loss rate of 10% for patients with Valganciclovir CMV prophylaxis and 25% for patients with pre-emptive therapy:

A Fisher's exact test with a 0.040 two-sided significance level will have 90% power to detect the difference between a Group 1 proportion, π_1, of 0.100 and a Group 2 proportion, π_2, of 0.250 when the sample size in each group is 150.

For the first step of the hierarchic test procedure within the first hypotheses system the following power can be calculated for the obtained sample size of the above calculation:

Assuming a CMV viremia rate of 17.5% for patients with valganciclovir CMV prophylaxis and 46.5% for patients with pre-emptive therapy results in a power of >99% for the sample size of 150 patients in each group for a Fisher's exact test with a 0.040 two-sided significance level.

For the second step of the hierarchic test procedure within the first hypotheses system the following power can be calculated for the obtained sample size of the above calculation:

Assuming a CMV disease rate of 3% for patients with valganciclovir CMV prophylaxis and 14% for patients with pre-emptive therapy results in a power of 90% for the sample size of 150 patients in each group for a Fisher's exact test with a 0.040 two-sided significance level.

Therefore the overall power of the hierarchic test procedure within the first hypotheses system is >80%.

For the second hypotheses system the following power can be calculated for the obtained sample size of the above calculation:

Assuming a value of 0.2 for the proteomics pattern for patients with valganciclovir CMV prophylaxis and a value of -0.3 for the proteomics pattern for patients with pre-emptive therapy results in a power of 99% for the sample size of 150 patients in each group for a two group t-test with a 0.010 two-sided significance level.

The gender distribution in this population is ca. 35% female to 65% male [4, 11, 22]. Given the sample size of 300 patients, ca. 100 female patients will be enrolled. This number will be sufficient to detect possible gender specific differences regarding the efficacy and safety of the investigational medicinal product according to § 7 Abs. 2 Nr. 12 GCP-V.

8.4 Replacement Policy (Ensuring Adequate Numbers of Evaluable Subjects)

8.4.1 For Subjects

No subject prematurely discontinued from the study for any reason will be replaced, however subjects who dropped out of the study will be asked to provide information about their survival, their graft survival and their immunsupressive medication in a non-interventional fashion by telephone to avoid a large amount of censored data in the survival analysis.

Patients will have to give a separate consent according to german data protection law (Bundesdatenschutzgesetz) to provide this information to Roche Pharma AG.

8.4.2 For Centers

A center may be replaced for the following administrative reasons:

- Excessively slow recruitment
- Poor protocol adherence

9 DATA QUALITY ASSURANCE

The overall procedures for quality assurance of clinical study data are described in the Roche Standard Operational Procedures.

Accurate and reliable data collection will be assured by verification and cross-check of the CRFs against the investigator's records by the study monitor (source document verification), and the maintenance of a drug-dispensing log by the investigator.

The data collected will be entered into the study database from the working copy of the CRF faxed from the site.

A comprehensive validation check program will verify the data and discrepancy reports will be generated accordingly for resolution by the investigator.

As patients complete the study (or prematurely withdraw) and their signed CRFs become available, a second data entry will be performed from the original, signed CRF. A comparison check will be run to identify and resolve any discrepancies between the first and second data entry.

10 STUDY COMMITTEES

A Study Review Board will be established by the mid of 2008 to quarterly review descriptive statistics of the study population.

11 REFERENCES

1. Wiltshire H et al. Pharmacokinetic profile of ganciclovir after its oral administration and from its prodrug, valganciclovir, in solid organ transplant recipients. *Clin Pharmacokinet,* 2005; 44:495-507.

2. Pescovitz MD et al. Valganciclovir results in improved oral absorption of ganciclovir in liver transplant recipients. *Antimicrob Agents Chemother*. 2000; 44:2811-5.

3. Martin DF et al. A controlled trial of valganciclovir as induction therapy for cytomegalovirus retinitis. *N Engl J Med*. 2002, 11; 346:1119-26

4. Paya C et al. Efficacy and safety of valganciclovir vs. oral ganciclovir for prevention of cytomegalovirus disease in solid organ transplant recipients. *Am J Transplant*. 2004, 4:611-20.

5. Babel N et al, Treatment of cytomegalovirus disease with valganciclovir in renal transplant recipients: a single center experience. *Transplantation*. 2004, 78:283-5.

6. Devyatko et al. Pre-emptive treatment with oral valganciclovir in management of CMV infection after cardiac transplantation. *J Heart Lung Transplant.* 2004; 23:1277-82.

7. Fellay J et al. Treatment of cytomegalovirus infection or disease in solid organ transplant recipients with valganciclovir. *Am J Transplant*. 2005 ; 5:1781-2

8. Singh N. Late-onset cytomegalovirus disease as a significant complication in solid organ transplant recipients receiving antiviral prophylaxis: a call to heed the mounting evidence. Clin Infect Dis. 2005, 40:704-8.

9. Weir M.R. Medical Management of Kidney Transplantation. 2005 Chapter 28: Cytomegalovirus in Renal Transplantation. Lippincott Williams & Wilkins

10. Sagedal S, et al. The Impact of Cytomegalovirus Infection and Disease on Rejection Episodes in Renal Allograft Recipients. *American Journal of Transplantation*, 2002; 2: 850–856.

11. Opelz G et al. Cytomegalovirus prophylaxis and graft outcome in solid organ transplantation: a collaborative transplant study report. *Am J Transplant.* 2004; 4:928-36.

12. EBPG (European Expert Group on Renal Transplantation) et al. European Best Practice Guidelines for Renal Transplantation (part 1). *Nephrol Dial Transplant.* 2000;15 Suppl 7:1-85

13. Preiksaitis JK et al. Canadian society of transplantation consensus workshop on cytomegalovirus management in solid organ transplantation final report. *Am J Transplant*. 2005; 5:218-27

14. AST (American Society of Transplantation). Cytomegalovirus. *Am J Transplant*. 2004, 4 Suppl 10: 51-58

15. Hodson EM et al. Antiviral medications to prevent cytomegalovirus disease and early death in recipients of solid-organ transplants: a systematic review of randomised controlled trials. *Lancet*. 2005, 365:2105-15.

16. Singh N et al. Efficacy of valganciclovir administered as pre-emptive therapy for cytomegalovirus disease in liver transplant recipients: impact on viral load and late-onset cytomegalovirus disease. *Transplantation*. 2005; 79:85-90.

17. Heemann U, Wenzel RR: CMV prophylaxis: what is valid in 2002? *Nephrol Dial Transplant*. 2002, 4:556-9.

18. Goodrich J, Khardori N. Cytomegalovirus: the taming of the beast? *Lancet*. 1997, 350:1718-9.

19. Rollag H et al. Cytomegalovirus DNA concentration in plasma predicts development of cytomegalovirus disease in kidney transplant recipients. *Clin Microbiol Infect*. 2002; 8:431-4.

20. Kletzmayr J et al. Long-term oral ganciclovir prophylaxis for prevention of cytomegalovirus infection and disease in cytomegalovirus high-risk renal transplant recipients. *Transplantation*. 2000, 70:1174-80.

21. Sester U et al: Differences in CMV-specific T-cell levels and long-term susceptibility to CMV infection after kidney, heart and lung transplantation. *Am J Transplant*. 2005, 5: 1483-9.

22. Sagedal S et al: Impact of early cytomegalovirus infection and disease on long-term recipient and kidney graft survival. *Kidney Int*. 2004, 66:329-37.

23. Wittke S et al: Detection of acute tubulointerstitial rejection by proteomic analysis of urinary samples in renal transplant recipients. Am J Transplant. 2005 in press

24. Hjelmesaeth J et al. Asymptomatic cytomegalovirus infection is associated with increased risk of new-onset diabetes mellitus and impaired insulin release after renal transplantation. *Diabetologia*. 2004; 47:1550-6.

25. Sharples LD et al. Diagnostic accuracy of coronary angiography and risk factors for post-heart-transplant cardiac allograft vasculopathy. *Transplantation*. 2003; 76:679-82.

26. Fateh-Moghadam et al. Cytomegalovirus infection status predicts progression of heart-transplant vasculopathy. *Transplantation*. 2003; 76:1470-4.

Part II - ETHICS AND GENERAL STUDY ADMINISTRATION

12 ETHICAL ASPECTS

12.1 Declaration of Helsinki/Good Clinical Practice

The Declaration of Helsinki (revision of 1996) is the accepted basis for clinical study ethics, and must be fully followed and respected by all engaged in research on human beings. Any exceptions must be justified and stated in the protocol.

Additionally it is the responsibility of all engaged in research on human beings to ensure that the study is performed in accordance with the international Good Clinical Practice standards and according to all local laws and regulations concerning clinical studies.

12.2 Patient Information and Informed Consent

It is the responsibility of the investigator to obtain written informed consent from each subject participating in this study, after adequate explanation of aim, importance, anticipated benefits, and potential hazards and consequences of the study according to § 40 Abs 2 and § 40 Abs. 2a AMG. Written informed consent must be obtained before any study specific procedures are performed. For subjects not qualified or incapable of giving legal consent, written consent must be obtained from the respective legal representative. It must be also explained to the subject that they are completely free to refuse to enter the study or to withdraw from it at any time for any reason without incurring any penalty or withholding of treatment on the part of the investigator.

By signing the consent form, the subject/patient agrees with the "unwiderrufliche datenschutzrechtliche Einwilligung" according to § 40 Abs. 2a AMG. The subject/patient also agrees to allow the monitor/auditor/health authorities to verify the collected patient data against the subject's/patient's original medical records for the purpose of source data verification.

The informed consent form personally signed and dated by the patient must be kept on file by the investigator(s), and documented in the case report form and the subject's medical records. The investigator confirms obtaining the written informed consent to the sponsor.

If new safety information results in significant changes in the risk/benefit assessment, the consent form should be reviewed and updated if necessary. All subjects (including those already being treated) should be informed of the new information and must give their written informed consent to continue in the study.

If the family doctors are informed of their patients’ participation in the clinical study, this should be mentioned in the consent form.

12.3 Independent Ethics Committees and Regulatory Authorities

12.3.1 Approval of the Study by the Federal Regulatory Authority and Independent Ethics Committees

According to §§ 40-42 of the German drug law (AMG) it is the responsibility of Roche to obtain and maintain independent approval from the federal regulatory authority (BfArM/PEI) and a positive opinion from the competent ethics committees to conduct the study.

The insurance coverage (study subject insurance) laid down in § 40 AMG is in force. For each subject, Hoffmann-La Roche AG has taken out an insurance with Zürich Versicherung AG (Germany), Frankfurt am Main, contract number 801.380.010.073.

In case of an multicenter study the sponsor names the "Leiter der klinischen Prüfung" (LKP) who has to be a physician with at least 2 years experience in the conduct of clinical trials of drugs according to § 4 (25) and § 40 (1) No. 5 AMG. In case of single center studies the principal investigator has to have the before mentioned 2 years experience, too.

12.3.2 Notification of the Study

According to § 67 German drug law (AMG) Roche is responsible to notify competent regional authority and the federal regulatory authority (BfArM/PEI) about the study and all principal investigators of the participating investigational sites. If no other agreements are made, Roche will take over responsibility for investigator's obligation to report (§ 12 (3) GCP-V).

12.3.3 Report and Documentation Obligation

The sponsor is responsible to comply with the report and documentation obligation according to § 63b AMG and § 13 GCP-V.

The investigator is responsible to comply with the report and documentation obligation according to § 12 GCP-V.

13 Conditions for Modifying the Protocol

Protocol modifications to ongoing studies must be made via amendment. The sponsor is responsible to obtain independent approval for the amendment from the federal regulatory authority (BfArM/PEI) and a positive opinion from the competent ethics committees if required according to § 10 GCP-V.

According to § 67 AMG amendments competent regional authorities and the federal regulatory authority must be notified about the amendment, if they concern items according to § 12 Abs. 1 GCP-V.

14 Discontinuation or Early Termination of the Study

Both the sponsor and the investigator reserve the right to terminate the study at any time. Should this be necessary, both parties will arrange the procedures on an individual study basis after review and consultation. In terminating the study, Roche and the investigator will assure that adequate consideration is given to the protection of the patient's interests.

Following criteria could lead to a discontinuation or early termination of the study:

- Safety reason regarding patients' safety
- Negative benefit/risk assessment due to new information
- Business considerations

In case of premature termination of the study all collected data have to be analyzed and a report has to be written. The sponsor has to inform the federal regulatory authority, the ethics committees and other authorities of member states of the European Union where the study is conducted within 15 days, giving detailed reason for the premature termination.

15 STUDY DOCUMENTATION, CRFS AND RECORD KEEPING

15.1 Investigator's Files/Retention of Documents

The Investigator must maintain adequate and accurate records to enable the conduct of the study to be fully documented and the study data to be subsequently verified. These documents should be classified into two different separate categories Investigator's Study File, and subject/patient data.

The Investigator's Study File will contain all essential documents as the protocol/amendments, Case Report and Query Forms, patient information and informed consent form, Ethics Committee and federal regulatory authority approval, notification of the federal regulatory authority and competent regional authorities, drug records, staff curriculum vitae and authorization forms and other appropriate documents/correspondence etc.

Patient data include patient hospital/clinic records (medical reports, OP reports appointment book, medical records, pathology and laboratory reports, ECG, EEG, X-ray, etc.) and signed informed consent forms and subject screening and eligibility screening forms.

The investigator must keep these two categories of documents on file for at least 15 years (or more as legally required) after completion or discontinuation of the study. The documents must be archived in a secure place and treated as confidential material.

Should the investigator wish to assign the study records to another party or move them to another location, Roche must be notified in advance.

If the investigator can not guarantee this archiving requirement at the investigational site for any or all of the documents, special arrangements must be made between the investigator and Roche to store these in a sealed container(s) outside of the site so that they can be returned sealed to the Investigator in case of a regulatory audit. Where source documents are required for the continued care of the patient, appropriate copies should be made for storing outside of the site.

Roche must archive the protocol, documentation, approvals and all other essential documents related to the study, including certificates that satisfactory audit and inspection procedures have been carried out, as long as the test product(s) remains on the market.

All documents must be archived in a secure place and treated as confidential material.

15.2 Source Documents and Background Data

The investigator shall supply the sponsor on request with any required background data from the study documentation or clinic records. This is particularly important when Case Report Forms are illegible or when errors in data transcription are suspected. In case of special problems and/or governmental queries or requests for audit inspections, it is also necessary to have access to the complete study records, provided that patient confidentiality is protected. According to the standards of the data protection law, all data obtained in the course of a clinical study must be treated with discretion in order to guarantee the rights of the patient's privacy.

15.3 Audits and Inspections

This study may be audited by Roche, any person authorized by Roche or the competent health authority in order to determine the authenticity of the recorded data and compliance with the study protocol.

The investigator should understand that source documents for this trial should be made available to appropriately qualified personnel from Roche/monitors/auditor/health authority inspectors after appropriate notification needed for source data verification and proper review of the study progress. The verification of the Case Report Form data must be by direct inspection of source documents. The investigator agrees to comply with Roche and regulatory authority requirements regarding the auditing of the study.

All material used in clinical studies are subjected to quality control.

15.4 Case Report Forms

For each patient enrolled, a Case Report Form must be completed and signed by the principal investigator or authorized delegate from the study staff. This also applies to records for those patients who fail to complete the study (even during a pre-randomization screening period if a Case Report Form was initiated). If a patient withdraws from the study, the reason must be noted on the Case Report Form. If a patient is withdrawn from the study because of a treatment-limiting adverse event, thorough efforts should be made to clearly document the outcome.

For this trial a standard electronic data capture solution with electronic case report forms (eCRF) will be used. All data are collected and documented in the web-based application TRI@L-IT delivered by clinIT AG in Freiburg. Online edit checks will verify crucial data points for completeness and consistency. Additionally in depth data validation by offline checks will be performed. Results of the offline checks will be fed into the integrated query management system as new discrepancies. Discrepancies will be followed up by a central data monitor.

The TRI@L-IT software used is fully validated and compliant with regulatory requirements such as "Arzneimittelgesetz", CGP, FDA CFR 21 part 11 and the EU GCP directive. Compliance has been shown by clinIT AG in independent audits and inspections.

16 Monitoring the Study

The monitor has the responsibility to familiarize the investigator(s) and the entire center staff involved in the study with all study procedures including the administration of study drug.

Roche must provide a trained monitor to assist the investigator(s) in conducting the clinical study. The monitor must visit the clinical study center on a regular basis and at least before the first subject has been enrolled, once during the course of the study, and at study completion. The monitor has the responsibility of reviewing the ongoing study with the investigator(s) to verify adherence to the protocol and to deal with any problems that arise. At all times Roche must maintain the confidentiality of the study documents. It is the responsibility of the study monitor to verify the study documents against the subject's original medical records.

The investigator (or his/her deputy) agrees to cooperate with the monitor to ensure that any problems detected in the course of these monitoring visits are resolved.

17 Confidentiality of Trial Documents and Subject Records

The investigator and the sponsor (or designee) must assure that according to the standards of the data protection law, all data obtained in the course of a clinical study must be treated with discretion in order to guarantee the rights of the patient's privacy. CRFs or other documents

should be submitted to the sponsor in a pseudonym manner. The investigator should keep a patient identification log showing codes and names. The investigator should maintain documents not for submission to Roche, e.g., subjects' written consent forms, in strict confidence.

18 Publication of Data

The study will be published in the clinical trial protocol registry and the clinical results database.

The sponsor is responsible for the timely reporting of study data. An integrated clinical study report (CSR) has to be completed one year after end of the study (whether completed or prematurely terminated). The report has to be approved by the responsible specialist chosen by the sponsor, the project manager of the CRO, the statistician and the principal investigator/LKP (for multicenter studies) by provision of their signatures.

The results of this study may be published or presented at scientific meetings as soon as after completion of the study. If this is foreseen, the investigator agrees to submit all manuscripts or abstracts to Roche prior to submission.

In a multicenter study, it must be ensured that the data from one center is not published before the publication of the whole study. Roche reserves the right to review the manuscript(s) before their submission for publication or presentation. This is not intended to restrict or hinder publication or presentation, but is to allow the sponsor to protect proprietary information and to provide comments based on information from other studies that may not yet be available to the investigator(s).

In accord with standard editorial and ethical practice, Roche will generally support publication of multicenter trials only in their entirety and not as individual center data. In this case, a coordinating investigator will be designated by mutual agreement.

Any formal publication of the study in which input of Roche personnel exceeded that of conventional monitoring will be considered as a joint publication by the investigator and the appropriate Roche personnel. Authorship will be determined by mutual agreement.

19 APPENDICES

19.1 Appendix 1 Adverse Events Categories for Determining Relationship to Test Drug

(a) Probable (must have first three)

This category applies to those adverse events which are considered, with a high degree of certainty, to be related to the test drug. An adverse event may be considered probable, if:

1. It follows a reasonable temporal sequence from administration of the drug.
2. It cannot be reasonably explained by the known characteristics of the subject's clinical state, environmental or toxic factors, or other modes of therapy administered to the subject.
3. It disappears or decreases on cessation or reduction in dose. (There are important exceptions when an adverse event does not disappear upon discontinuation of the drug, yet drug-relatedness clearly exists: e. g. (1) bone marrow depression, (2) tardive dyskinesias.)
4. It follows a known pattern of response to the suspected drug.
5. It reappears upon rechallenge.

(b) Possible (must have first two)

This category applies to those adverse events in which the connection with the test drug administration appears unlikely but cannot be ruled out with certainty. An adverse event may be considered possible if, or when:

1. It follows a reasonable temporal sequence from administration of the drug.
2. It may have been produced by the subject's clinical state, environmental or toxic factors, or other modes of therapy administered to the subject.
3. It follows a known pattern of response to the suspected drug.

(c) Remote (must have first two)

In general, this category is applicable to an adverse event which meets the following criteria:

1. It does <u>not</u> follow a reasonable temporal sequence from administration of the drug.
2. It may readily have been produced by the subject's clinical state, environmental or toxic factors, or other modes of therapy administered to the subject.
3. It does not follow a known pattern of response to the suspected drug.
4. It does not reappear or worsen when the drug is readministered.

(d) Unrelated

This category is applicable to those adverse events which are judged to be clearly and incontrovertibly due only to extraneous causes (disease, environment, etc.) and do not meet the criteria for drug relationship listed under remote, possible, or probable.

	Probable	Possible	Remote	Unrelated
Clearly due to extraneous causes	–	–	–	+
Reasonable temporal association with drug administration	+	+	–	–
May be produced by subject clinical state, etc.	–	+	+	+
Known response pattern to suspected drug	+	+	–	–
Disappears or decreases on cessation or reduction in dose	+	–	–	–
Reappears on rechallenge	+	–	–	–

19.2 Appendix 2 Definitions according to AMG and GCP-V, ICH Guidelines for Clinical Safety Data Management, Definitions and Standards for Expedited Reporting, Topic E2

An adverse event is any untoward medical occurrence in a patient or clinical investigation subject, administered a pharmaceutical product and which does not necessarily have to have a causal relationship with this treatment.

Adverse reactions are all untoward and unintended responses to an investigational medicinal product related to any dose administered.

A serious adverse event or serious adverse reaction is any experience that suggests a significant hazard, contraindication, side effect or precaution. It is any Adverse Event that at any dose fulfills at least one of the following criteria:

- is fatal (results in death)
  (NOTE: death is an outcome, not an event)
- is life-threatening
  (NOTE: the term "life-threatening" refers to an event in which the patient was at immediate risk of death at the time of the event; it does not refer to an event which could hypothetically have caused a death had it been more severe.)
- required in-patient hospitalization or prolongation of existing hospitalization
- results in persistent or significant disability/incapacity
- is a congenital anomaly/birth defect
- is medically significant or requires intervention to prevent one or other of the outcomes listed above

Medical and scientific judgment should be exercised in deciding whether expedited reporting to the sponsor is appropriate in other situations, such as important medical events that may not be immediately life-threatening or result in death or hospitalization but may jeopardize the patient or may require intervention to prevent one of the outcomes listed in the definitions above. These situations should also usually be considered serious.

Examples of such events are intensive treatment in an emergency room or at home for allergic bronchospasm; blood dyscrasias or convulsions that do not result in hospitalization; or development of drug dependency or drug abuse.

An unexpected Adverse Event is one, the nature or severity of which is not consistent with the applicable product information.

Causality is initially assessed by the investigator. With respect to report and documentation obligation (regulatory authorities, ethics committees and other investigators) for Serious Adverse Events, causality can be one of 2 possibilities:

- No (unrelated; equals not drug related).
- Yes (remotely, possibly, probably or definitely drug related).

All adverse events not assessed as definitive "not drug related" by either the investigator or Roche will be considered as adverse drug reaction.

A suspected unexpected serious adverse reaction (SUSAR) is a serious adverse reaction, the nature, or severity of which is not consistent with the applicable product information.

It is important that the severity of an adverse event is not confounded with the seriousness of the event. For example, vomiting which persists for many hours may be severe, but is not necessarily a serious adverse event. On the other hand, stroke which results in only a limited degree of disability may be considered a mild stroke, but would be a serious adverse event.

A serious adverse event occurring during the study or which comes to the attention of the investigator within 15 days after stopping the treatment or during the protocol-defined follow-up period, if this is longer, whether considered treatment-related or not, must be reported. In addition, a serious adverse event that occurs after this time, if considered related to test "drug", should be reported.

Such preliminary reports will be followed by detailed descriptions later which will include copies of hospital case reports, autopsy reports and other documents when requested and applicable.

For serious adverse events, the following must be assessed and recorded on the adverse events page of the Case Report Form: intensity, relationship to test substance, action taken, and outcome to date.

Document and report obligation have to be adhered according to the national and international laws and regulations.

Contact details and Fax No. for SAE and pregnancy reporting refer to page 9.

19.3 Appendix 3 Grading of Severity of Adverse Events and Laboratory Abnormalities

ITEM	Grade 1 Toxicity	Grade 2 Toxicity	Grade 3 Toxicity	Grade 4 Toxicity
**HEMATOLOGY**				
Hemoglobin	9.5 - 10.5 g/dL	8.0 - 9.4 g/dL	6.5 - 7.9 g/dL	<6.5 g/dL
Absolute Neutrophil Count	1000 – 1500/mm^3	750 – 999/mm^3	500 – 749/mm^3	<500/mm^3
Platelets	75,000–99,000/mm^3	50,000–74,999/mm^3	20,000–49,999/mm^3	<20,000/mm^3
Prothrombin Time (PT)	1.01– 1.25 × ULN[ULN]	1.26 – 1.5 × ULN	1.51 – 3.0 × ULN	>3 × ULN
Partial Prothrombin Time (PTT)	1.01 –1.66 × ULN	1.67 – 2.33 × ULN	2.34 – 3.0 × ULN	>3 × ULN
Fibrinogen	0.75 – 0.99 × LLN	0.50 – 0.74 × LLN	0.25 –0.49 × LLN	<0.25 × LLN
Fibrin Split Product	20 – 40 µg/ml	41 – 50 µg/ml	51 – 60 µg/ml	>60 µg/ml
Methemoglobin	5 – 9.9%	10.0 – 14.9%	15.0 – 19.9%	>20%
**CHEMISTRIES**				
Hyponatremia	130 – 135 meq/dL	123 – 129 meq/L	116 – 122 meq/L	<116 meq/L
Hypernatremia	146 – 150 meq/L	151 – 157 meq/L	158 – 165 meq/L	>165 meq/L
Hypokalemia	3.0 – 3.4 meq/L	2.5 – 2.9 meq/L	2.0 – 2.4 meq/L	<2.0 meq/L
Hyperkalemia	5.6 – 6.0 meq/L	6.1 – 6.5 meq/L	6.6 – 7.0 meq/L	>7.0 meq/L
Hypoglycemia	55 – 64 mg/dL	40 – 54 mg/dL	30 – 39 mg/dL	<30 mg/dL
Hyperglycemia (not fasting and without prior hx diabetes)	116 –160 mg/dL	161 – 250 mg/dL	251 – 500 mg/dL	>500 mg/dL or ketoacidosis or seizures
Hypocalcemia corrected for albumin	7.8 –8.4 mg/dL	7.0 – 7.7 mg/dL	6.1 – 6.9 mg/dL	<6.1 mg/dL
Hypercalcemia corrected for albumin	10.6 –11.5 mg/dL	11.6 – 12.5 mg/dL	12.6 – 13.5 mg/dL	>13.5 mg/dL
Hypomagnesemia	1.2 – 1.4 meq/L	0.9 – 1.1 meq/L	0.6 – 0.8 meq/L	<0.6 meq/L
Hypophosphatemia	2.0 – 2.4 mg/dL	1.5 – 1.9 mg/dL	1.0 – 1.4 mg/dL	<1.0 mg/dL
Hyperbilirubinemia	1.1 – 1.5 × ULN	1.6 – 2.5 × ULN	2.6 – 5 × ULN	>5 × ULN
Blood urea nitrogen (BUN)	1.25 –2.5 × ULN	2.6 – 5 × ULN	5.1 – 10 × ULN	>10 × ULN
Creatinine	1.1 – 1.5 × ULN	1.6 – 3.0 × ULN	3.1 – 6 × ULN	>6 × ULN
**ENZYMES**				
AST (SGOT)	1.25 – 2.5 × ULN	2.6 – 5 × ULN	5.1 – 10 × ULN	>10 × ULN
ALT (SGPT)	1.25 – 2.5 × ULN	2.6 – 5 × ULN	5.1 – 10 × ULN	>10 × ULN
γ-GT	1.25 – 2.5 × ULN	2.6 – 5 × ULN	5.1 – 10 × ULN	>10 × ULN

ULN = Upper Limit Normal

LLN = Lower Limit Normal

ITEM	Grade 1 Toxicity	Grade 2 Toxicity	Grade 3 Toxicity	Grade 4 Toxicity
Alkaline Phosphatase	1.25 – 2.5 × ULN	2.6 – 5 × ULN	5.1 – 10 × ULN	>10 × ULN
Amylase	1.1 - 1.5 × ULN	1.6 – 2.0 × ULN	2.1 – 5.0 × ULN or clinical pancreatitis	>5.1 × ULN or clinical pancreatitis
Lipase	1.1 – 1.5 × ULN	1.6 – 2.0 × ULN	2.1 – 5.0 × ULN	>5.1 × ULN
Pancreatic Amylase	1.1 – 1.5 × ULN	1.6 – 2.0 × ULN	2.1 – 5.0 × ULN	>5.1 × ULN
CPK	1.1 – 2.0 × ULN	2.1 – 4.0 × ULN	4.1 – 6.0 × ULN	≥6.1 × ULN
**URINALYSIS**				
Proteinuria	1 + or <0.3% or <3 g/L or 200 mg - 1 g loss/day	2 - 3 + or 0.3% – 1.0% or 3 – 10 g/L or 1 – 2 g loss/day	4 + or >1.0% or >10 g/L or 2 – 3.5 g loss/day	nephrotic syndrome or >3.5 g loss/day
Hematuria	microscopic only	gross, no clots	gross plus clots	obstructive or required transfus.

19.3 Appendix 3 Grading of Severity of Adverse Events and Laboratory Abnormalities

ITEM	Grade 1 Toxicity	Grade 2 Toxicity	Grade 3 Toxicity	Grade 4 Toxicity
**CARDIAC**				
Cardiac rhythm		asymptomatic, transient dysrhythmia, no Rx required	recurrent/persistent dysrhythmia; symptomatic Rx required	unstable dysrhythmia requires hospitalization and Rx
Hypertension	Transient, increase >20 mm; no Rx	recurrent, chronic increase >20 mm/Hg, Rx required	required acute Rx; outpatient hospitalization possible	requires hospitalization
Hypotension	transient orthostatic hypotension; no Rx	symptoms correctable with oral fluid Rx	requires IV fluids; no hospitalization required	requires hospitalization
Pericarditis	minimal effusion	mild/mod asymptomatic effusion; no Rx	symptomatic effusion, pain, EKG changes	tamponade or pericardiocentesis or surgery required
Hemorrhage, blood loss		mildly symptomatic, no transfusion required	gross blood loss or 1 – 2 units transfused	massive blood loss or ≥3 units transfused
**RESPIRATORY**				
Bronchospasm, Acute	transient; no Rx; FEV_1[b] < 80% – 70% (or peak flow)	req. Rx, normalizes with bronchodilator; FEV_1 50% < 70% (or peak flow)	no normalization with bronchodilator; FEV_1 25% < 50% (or peak flow), retractions	cyanosis; FEV_1 <25% (or peak flow) or intubated
Dyspnea	Dyspnea on exertion	Dyspnea with normal activity	Dyspnea at rest	Dyspnea requiring O_2 therapy
**GASTROINTESTINAL**				
Stomatitis,<br>Oral discomfort; Dysphagia	mild discomfort; no difficulty swallowing	difficulty swallowing but able to eat and drink	unable to swallow solids	unable to drink<br>fluids; requires IV<br>fluids
Nausea	mild or transient; maintains reasonable intake	moderate discomfort; intake decreased for <3 days	severe discomfort; or minimal intake for ≥3 days	requires hospitalization
Vomiting	mild or transient; 2 – 3 episodes per day or mild vomiting lasting <1 week	moderate or persistent; 4 – 5 episodes per day; or vomiting lasting ≥1 week	severe vomiting of all food/fluids in 24 hrs or orthostatic hypotension or IV Rx required	hypotensive shock or hospitalization required for IV fluid therapy
Constipation	mild	moderate	severe	distention with vomiting

[b] FEV = Forced Expiratory Volume

19.3 Appendix 3 Grading of Severity of Adverse Events and Laboratory Abnormalities

ITEM	Grade 1 Toxicity	Grade 2 Toxicity	Grade 3 Toxicity	Grade 4 Toxicity
Diarrhea	Mild OR transient; 3 – 4 loose stools per day OR mild diarrhea lasting <1 week	Moderate OR persistent; 5 – 7 loose stools per day OR diarrhea lasting ≥1 week	Bloody diarrhea; OR orthostatic hypotension; OR >7 loose stools per day OR IV Rx required	Hypotensive shock OR hospitalization required
**NEURO/NEUROMUSCULAR**				
Neuro-cerebellar	slight incoordination dysdiadocho-kinesia	intention tremor OR dysmetria OR slurred speech OR nystagmus	ataxia requiring assistance to walk or arm incoordination interfering with ADL2[c]	unable to stand
Mood			severe mood changes requiring medical intervention	acute psychosis, requires hospitalization
Paresthesia (burning; tingling, etc.)	Mild discomfort; no Rx required	Moderate discomfort; non-narcotic analgesia required	Severe discomfort; OR narcotic analgesia required with symptomatic improvement	incapacitating OR not responsive to narcotic analgesia
Neuro-motor	mild weakness in muscle of feet but able to walk and/OR mild increase OR decrease in reflexes	moderate weakness in feet (unable to walk on heels and/or toes), mild weakness in hands, still able to do most hand tasks and/OR loss of previously present reflex OR development of hyperreflexia and/OR unable to do deep knee bends due to weakness	marked distal weakness (unable to dorsiflex toes or foot drop), and moderate proximal weakness e.g. in hands interfering with ADL[c] and/OR requiring assistance to walk and/OR unable to rise from chair unassisted	confined to bed OR wheel chair because of muscle weakness
Neuro-sensory	mild impairment (decreased sensation, e.g. vibratory, pinprick, hot/cold in great toes) in focal area or symmetrical distribution	moderate impairment (moderately decreased sensation, e.g. vibratory pinprick, hot/cold to ankles) and/or joint position or mild impairment that is not symmetrical	severe impairment (decreased or loss of sensation to knees or wrists) or loss of sensation of at least moderate degree in multiple different body areas (i.e. upper and lower extremities)	sensory loss involves limbs and trunk

[c] ADL = Activities of Daily Living

19.3 Appendix 3 Grading of Severity of Adverse Events and Laboratory Abnormalities

ITEM	Grade 1 Toxicity	Grade 2 Toxicity	Grade 3 Toxicity	Grade 4 Toxicity
**OTHER PARAMETERS**				
Fever; oral, >12 hours	37.7 – 38.5C or 100.0 – 101.5F	38.6– 39.5C or 101.6 – 102.9F	39.6 – 40.5C or<br>103 –105F	>40.5C or >105F
Headache	mild, no Rx required	moderate OR Rx required (non-narcotic	severe OR responds to initial narcotic Rx	intractable OR requiring repeated narcotic Rx
Fatigue / Malaise	normal activity reduced <25%	normal activity<br>reduced 25 – 50%	normal activity reduced >50%; can't work	unable to care for self
Allergic reaction	pruritus without rash	localized urticaria	generalized urticaria angioedema	anaphylaxis
Cutaneous/Rash/<br>Dermatitis	erythema, pruritus	diffuse maculopapular rash OR dry desquamation	vesiculation OR moist desquamation OR ulceration	any one: exfoliative dermatitis, mucous membrane involvement, suspected Stevens-Johnson (TEN), erythema multiforme, necrosis requiring surgery
Myositis	Mild symptoms with an increased CPK OR asymptomatic with increased CPK plus a positive EMG or biopsy	Positive EMG or muscle biopsy and either - mild symptoms with an increased CPK asymptomatic with an increased CPK >4 weeks OR moderate myalgias for <4 weeks requiring non-steroidal anti-inflammatory agents; OR difficulty with mobility	Positive EMG OR muscle biopsy and either moderate to severe myalgias for >4 weeks nonsteroidal anti-inflammatory agents; OR needs some assistance in walking	Positive EMG OR muscle biopsy and either severe muscle pain not related to exercise, requiring narcotics; OR muscle weakness with inability to walk requiring assistance OR acute rhabdomyolysis with muscle necrosis

19.4 Appendix 4 Assessment of acute allograft rejection

I. Clinical Assessment

A presumptive diagnosis of an acute episode of rejection will be based on one or more of the following findings:

- Temperature >100°F (37.5°C), orally
- Graft swelling
- Graft tenderness
- >0.3 mg/dl (or equivalent) rise in serum creatinine
- Rising blood pressure
- Oliguria
- Reduced flow in perfusion, extraction or excretion profile on renal scan
- Ultrasound findings consistent with rejection

19.4 Appendix 4 Assessment of acute allograft rejection (Cont.)

II. Histologic Assessment

The table attached is taken from the latest up-date to the Banff 97 Criteria in 2003.

Banff 97 Diagnostic Categories for Renal Allograft Biopsies

1. Normal - Normal, minor changes, or infiltrates without tubular invasion	
2. Antibody mediated rejection – Rejection due, at least in part, to documented anti-donor antibody	
Grade I	ATN-like – C4d+, minimal inflammation
Grade II	Capillary – margination and/or thromboses, C4d+
Grade III	Arterial – transmural arteritis/fibrinoid necrosis (v3), C4d+
3. Borderline changes - Very mild lymphocyte invasion of tubules (tubulitis)	
4. **Acute rejection** – T-cell mediated rejection	
Grade I **Mild**	(A) Significant interstitial infiltration (>25% of parenchyma affected) and foci of moderate tubulitis<br>(B) Significant interstitial infiltration (>25% of parenchyma affected) and foci of severe tubulitis
Grade II **Moderate**	(A) Mild to moderate intimal arteritis<br>(B) Severe intimal arteritis comprising >25% of the luminal area
Grade III **Severe**	"Transmural" arteritis and/or arterial fibrinoid change and necrosis of medial smooth muscle cells with accompanying lymphocytic inflammation
5. Chronic/Sclerosing allograft nephropathy - Interstitial fibrosis, tubular atrophy (new onset) arterial fibrous intimal thickening	
Grade I Mild	Mild interstitial fibrosis and tubular atrophy
Grade II Moderate	Moderate interstitial fibrosis and tubular atrophy
Grade III Severe	Severe interstitial fibrosis and tubular atrophy and tubular loss
6. Other	

Histological Assessment

Acute tubular necrosis - Tubular cell loss and necrosis, regenerative changes.
Cyclosporine toxicity - Hyaline arteriolar thickening (new onset, not present in implantation biopsy) and/or extensive isometric vacuolization of tubules, smooth muscle degeneration, thrombotic microangiopathy

19.5 Appendix 5 Secondary Basic Research Project 2 (SBRP 2)

1. Objectives and Background of the Research Project

The primary objective of this research project is to obtain a single blood sample from patients enrolled in the associated protocol ML 19313 for the purpose of Single Nucleotide Polymorphism (SNP) Allele analysis in renal allograft recipients IgG seropositive for CMV at risk for active CMV replication and/or CMV disease during the first year after transplantation.

Cytomegalovirus (CMV) remains the most important serious infection complicating solid organ transplantation (SOT). CMV infection can result in CMV disease, which, in severe cases, can lead to hospitalization, morbidity and in some cases, death. Valganciclovir a valyl ester prodrug of ganciclovir, is currently licensed for the induction and maintenance therapy of CMV retinitis in Acquired Immune Deficiency Syndrome (AIDS) patients and for the prevention of CMV disease in high risk (donor CMV positive / recipient CMV negative) patients in SOT.

Preliminary studies have suggested that genetic variation at a drug target or at other points of targeted biosynthetic pathways may influence drug response [1-4]. Polymorphisms in genes whose products are known to be implicated in modulating the human immune response, such as toll like receptor 4 and Interleukin-10 have been reported to be associated with more severe infectious episodes in renal allograft recipients and increased susceptibility to Herpesvirus infections respectively [5-6]. On the other hand a protective role against CMV infection was reported for Tumour Necrosis Factor-alpha and Interleukin-1 Receptor Antagonist polymorphisms which are known to be associated with a strong inflammatory response [7]. These observations raise the possibility that common variants in the human genome may influence the clinical utility of Valganciclovir. The coding genes for these moieties, together with functionally related elements, present themselves as strong candidates for genetic studies of Valganciclovir (see Table 1).

Table 1 Genes analyzed for Single Nucleotide Polymorphism (SNP) Alleles in SBRP 2

Gene	Gene Product
TLR4	Toll-like receptor 4
IL1RA	Interleukin 1 Receptor Antagonist
IL10	Interleukin 10
IL17	Interleukin 17
IL18	Interleukin 18
IL18BP	Interleukin 18 binding protein
IFNG	Interferon gamma
TNFalpha	Tumor Necrosis Factor alpha

ABCA1	ABC transporter A1
MDR1	Multidrug resistance gene 1
MRP2	Multidrug resistance protein 2
P2X7R	P2X7 receptor
CYP3A4	Cytochrom P450 A4
CYP3A5	Cytochrom P450 A5

Only genes listed in Table 1 will be analyzed in the SBRP 2 for Single Nucleotide Polymorphism Alleles. The identified SNP alleles of the genes shown in Table 1 gathered through the analysis of samples in the SBRP 2 is hoped to improve graft and patient survival in renal allograft recipients IgG seropositive for CMV at risk for active CMV infection and/or CMV disease by:

- predicting which renal allograft recipients are more likely to respond to Valganciclovir
- predicting which renal allograft recipients are likely to progress to more severe disease states
- predicting which renal allograft recipients are susceptible to developing adverse side effects

The SBRP 2 will be conducted in compliance with the protocol, Good Clinical Practice (ICH GCP) and any applicable regulatory requirements.

2. Design

This SBRP 2 protocol may be conducted in conjunction with the main protocol for study ML 19313.

ML 19313 is a randomized multicenter trial comparing Valganciclovir CMV prophylaxis versus pre-emptive therapy after renal transplantation using proteomics for monitoring of graft alteration. The primary objective of this trial is to compare in CMV positive renal allograft recipients the efficacy of valganciclovir (900 mg once daily) prophylaxis applied until Day 100 post transplant with no prophylaxis under the condition of pre-emptive therapy of active CMV infection. The secondary objective of this trial is to investigate the influence of both CMV prevention concepts on the occurrence of direct and indirect effects of active CMV infections: CMV disease (direct); renal function, rejections, opportunistic infections, graft- and patient survival, diabetes (indirect effects). In addition the safety of both concepts will be investigated by evaluating side effects, leucopenia/neutropenia, and infections.

3. Population

3.1 Target Population

All patients enrolled in the main protocol ML 19313 are eligible for enrolment in the SBRP 2. It is expected that approximately 200 patients may participate.

Participation in this SBRP 2 protocol is entirely voluntary.

3.2 Inclusion Criteria

For inclusion into the SBRP 2, a patient must satisfy the following criteria:

- Written informed consent previously obtained for the main protocol ML 19313
- Enrolment in main protocol ML 19313
- Written informed consent for the SBRP 2

3.3 Exclusion Criteria

There are no exclusion criteria for this research project.

4. Schedule of Assessments and Procedures

4.1 Procedures / Assessments

After written informed consent for associated clinical study ML 19313 has been obtained from a patient, the SBRP 2 and blood sampling procedure will be explained. The patient will be asked if he/she wishes to participate and written informed consent may be obtained specifically for the SBRP 2.

A single SBRP 2 blood sample may be obtained at the same time as blood sampling in the clinical protocol ML 19313 to avoid repeated venepuncture. The SBRP 2 sample can be collected at any time during the conduct of the associated clinical study (ML 19313). Dates of consent and blood sample collection should be recorded on the corresponding SBRP 2 page in the eCRF of associated study ML 19313.

4.2 Sampling Procedures

Study sites will be supplied with kits containing plastic 9 ml EDTA tubes for sample collection, 50 ml outer storage tubes for sample protection and patient specific labels containing the same individual patient number as used in the associated clinical study ML 19313. Once informed consent is obtained, 9 ml of blood should be collected by venepuncture into the 9 ml EDTA blood collection tube. The blood sample should be carefully mixed with the anticoagulant in the tube by inverting the tube several times. The label should be placed on the 9 ml tube and then this tube is placed in the 50 ml outer storage tube. No label is required on the

outer storage tube. The samples should then be frozen and stored at -20°C. Blood samples will be shipped on dry ice to the Sample Repository 2 (SR 2) located at Medizinische Klinik III - Nephrologie; Klinikum der J.W. Goethe Universität; Theodor Stern Kai 7; 60590 Frankfurt am Main by Globex24 Life Science Logistics. At the site DNA will be isolated out of each patient specific sample, transferred to a new tube and labeled with the same individual patient number as used for the patient in the associated study ML 19313. Finally each sample will be forwarded to the SR 2 for storage and genetic analysis. DNA samples as well as the obtained genetic information will be captured on the corresponding SBRP 2 page in the eCRF of the associated study ML 19313. At the end of the associated clinical study ML 19313, clinical data from ML 19313 will be linked to genotype information and analyzed as indicated in chapter 5. No more than 15 years after the end of the associated study (database closure), all blood and DNA samples will be destroyed.

5. Statistical Analysis

A descriptive analysis of differences between drug effects according to differing alleles, genotypes and/or haplotypes may be performed.

Summary statistics may be generated for the primary endpoint and major secondary endpoints as deemed appropriate.

Analysis of these same endpoints may be undertaken as set out in the main protocol which may additionally adjust for the effect of allele, genotype and/or haplotype.

The treatment group by allele, genotype and/or haplotype interaction may also be assessed and any significance may be explored further.

Descriptive demographic and key safety data may be presented by treatment group and allele, genotype and/or haplotype.

All summaries and analyses may be performed on the subset of the Intent to teat (ITT) population who additionally consented to this protocol.

6. Withdrawal of Patients from the SBRP 2

Patients have the right to withdraw their informed consent for the SBRP 2 as well as their sample from the Sample Repository 2 (SR 2) located at Medizinische Klinik III - Nephrologie; Klinikum der J.W. Goethe Universität; Theodor Stern Kai 7; 60590 Frankfurt am Main, at any time for any reason during their participation in the clinical study to which this SBRP 2 is associated (ML 19313). Withdrawal of informed consent for SBRP 2 will not affect the participation of the patient in the associated study ML 19313.

If a patient wishes to withdraw his/her sample, the investigator must inform the Roche monitor and enter the date of withdrawal in the patient´s Case Report Form (CRF). Within Roche, the request for sample withdrawal will be forwarded to the SBRP 2 project leader. If the sample is already at the Sample Repository 2 in Frankfurt, the SBRP 2 project leader will issue confirmation of the withdrawal, which will be forwarded to the investigator. If the sample is still at the investigator site at the time a patient wishes to withdraw his/her sample, the investigator must inform the Roche monitor as before, destroy the sample and sign the SBRP 2 Patient Withdrawal Form to confirm that this has been done. The Roche monitor will forward

confirmation of destruction, recorded on the Patient Withdrawal Form, to the SBRP 2 project leader. Genetic data obtained before withdrawal of participation will be included in the final statistical analysis of the SBRP 2.

7. Access to Source Documents

Roche monitors and auditors will have direct access to appropriate parts of records relating to patients participating in this research project and the associated study ML 19313 for the purposes of verifying the data provided to Roche. The sites will permit monitoring, audits, IRB/IEC review, and regulatory inspections by providing direct access to source data and documents related to the SBRP 2.

8. Ethical Aspects

Please refer to Section 12. “Ethical Aspects” in the protocol for study ML 19313.

9. Study Documentation, CRF and Record Keeping

Please refer to Section 15. “Study Documentation, CRFs and record keeping” in the protocol for study ML 19313.

10. Confidentiality of Study Documents and Patient Records

Delivery, storage, processing and measurement of the SBRP 2 sample as well as the obtained genetic data will be documented in a defined SBRP 2 chapter of the eCRF of the associated study ML 19313. The SBRP 2 chapter will not be linked with other chapters of the eCRF of ML 19313 to guarantee that only at the end of the associated clinical study ML 19313 clinical data from ML 19313 can be linked with genetic data obtained in the SBRP 2. The documentation of data for the SBRP 2 will be done by a study nurse employed at Medizinische Klinik III - Nephrologie; Klinikum der J.W. Goethe Universität; Theodor Stern Kai 7; 60590 Frankfurt am Main who will only have access to the SBRP 2 chapter of the eCRF and who will not be involved in the documentation of patient specific data for the associated clinical study ML 19313. Investigators as well as study nurses of the associated clinical study ML 19313 will not have access to the SBRP 2 chapter of the eCRF. Additionally investigators as well as patients participating in the associated clinical trial ML 19313 will not be provided with data generated in the SBRP 2.

For further details please refer to Section 17. “Confidentiality of trial documents and subject records” in the protocol for study ML 19313.

11. Publication of Data

Please refer to Section 18. “Publication of Data” in the protocol for study ML 19313.

12. References

1. Farlow MR, Lahiri DK, Poirier J, Davignon J, Hui S. Apolipoprotein E genotype and gender influence response to tacrine therapy. Ann N Y Acad Sci 1996;802:101-110

2. Poirier J, Delisle MC, Quirion R, Aubert I, Farlow M, Lahiri D, Hui S, Bertrand P, Nalbantoglu J, Gilfix BM, Gauthier S. Apolipoprotein E4 allele as a predictor of cholinergic deficits and treatment outcome in Alzheimer disease. Proc Natl Acad Sci U S A 1995; 92:12260-12264

3. Richard F, Helbecque N, Neuman E, Guez D, Levy R, Amouyel P. APOE genotyping and response to drug treatment in Alzheimer's disease. Lancet 1997; 349:539

4. Kuivenhoven JA, Jukema JW, Zwinderman AH, de Knijff P, McPherson R, Bruschke AV, Lie KI, Kastelein JJ The role of a common variant of the cholesteryl ester transfer protein gene in the progression of coronary atherosclerosis. The Regression Growth Evaluation Statin Study Group. N Engl J Med 1998; 338:86-93

5. Ducloux D, Deschamps M, Yannaraki M, Ferrand C, Bamoulid J, Saas P, Kazory A, Chalopin JM, Tiberghien P. Relevance of Toll-like receptor-4 polymorphisms in renal transplantation. Kidney Int 2005; 67:2454-2461

6. Hurme M, Haanpää M, Nurmikko T, Wang XY, Virta M, Pessi T, Kilpinen S, Hulkkonen J, Helminen M. IL-10 gene polymorphism and herpesvirus infections. J Med Virol 2003;70 Suppl 1:S48-50

7. Hurme M, Helminen M. Resistance to human cytomegalovirus infection may be influenced by genetic polymorphisms of the tumour necrosis factor-alpha and interleukin-1 receptor antagonist genes. Scand J Infect Dis 1998;30:447-449

13. SBRP 2 Project Leader

Prof. Dr. med. Ingeborg Hauser
Medizinische Klinik III - Nephrologie
Klinikum der J.W. Goethe Universität
Theodor Stern Kai 7
60590 Frankfurt am Main

Telephone Number: 0049 69 6301 6668
Fax Number: 0049 69 6301 5576
Email: i.hauser@em.uni-frankfurt.de
